# Isolation of singlet carbene derived 2-phospha-1,3-butadienes and their sequential one-electron oxidation to radical cations and dications†

**DOI:** 10.1039/c9sc05598c

**Published:** 2020-01-06

**Authors:** Mahendra K. Sharma, Sebastian Blomeyer, Timo Glodde, Beate Neumann, Hans-Georg Stammler, Alexander Hinz, Maurice van Gastel, Rajendra S. Ghadwal

**Affiliations:** Molecular Inorganic Chemistry and Catalysis, Inorganic and Structural Chemistry, Center for Molecular Materials, Faculty of Chemistry, Universität Bielefeld Universitätsstrasse 25 Bielefeld D-33615 Germany rghadwal@uni-bielefeld.de; Institute of Inorganic Chemistry, Karlsruhe Institute of Technology (KIT) Engesserstr. 15 D-76131 Karlsruhe Germany; Max-Planck-Institut für Kohlenforschung, Molecular Theory and Spectroscopy Kaiser-Wilhelm-Platz 1 Mülheim an der Ruhr D-45470 Germany

## Abstract

A synthetic strategy for the 2-phospha-1,3-butadiene derivatives [{(IPr)C(Ph)}P(cAAC^Me^)] (**3a**) and [{(IPr)C(Ph)}P(cAAC^Cy^)] (**3b**) (IPr = C{(NDipp)CH}_2_, Dipp = 2,6-iPr_2_C_6_H_3_; cAAC^Me^ = C{(NDipp)CMe_2_CH_2_CMe_2_}; cAAC^Cy^ = C{(NDipp)CMe_2_CH_2_C(Cy)}, Cy = cyclohexyl) containing a C=C–P=C framework has been established. Compounds **3a** and **3b** have a remarkably small HOMO–LUMO energy gap (**3a**: 5.09; **3b**: 5.05 eV) with a very high-lying HOMO (−4.95 eV for each). Consequently, **3a** and **3b** readily undergo one-electron oxidation with the mild oxidizing agent GaCl_3_ to afford radical cations [{(IPr)C(Ph)}P(cAAC^R^)]GaCl_4_ (R = Me **4a**, Cy **4b**) as crystalline solids. The main UV-vis absorption band for **4a** and **4b** is red-shifted with respect to that of **3a** and **3b**, which is associated with the SOMO related transitions. The EPR spectra of compounds **4a** and **4b** each exhibit a doublet due to coupling of the unpaired electron with the ^31^P nucleus. Further one-electron removal from the radical cations **4a** and **4b** is also feasible with GaCl_3_, affording the dications [{(IPr)C(Ph)}P(cAAC^R^)](GaCl_4_)_2_ (R = Me **5a**, Cy **5b**) as yellow crystals. The molecular structures of compounds **3–5** have been determined by X-ray diffraction and analyzed by DFT calculations.

## Abstract 2

The 2-phospha-1,3-butadiene derivatives **3** are readily accessible by reduction of **2** with Mg. Sequential one-electron oxidation of **3** with GaCl_3_ leads to the formation of radical cations **4** and dications **5** as crystalline solids.

## Graphical abstract

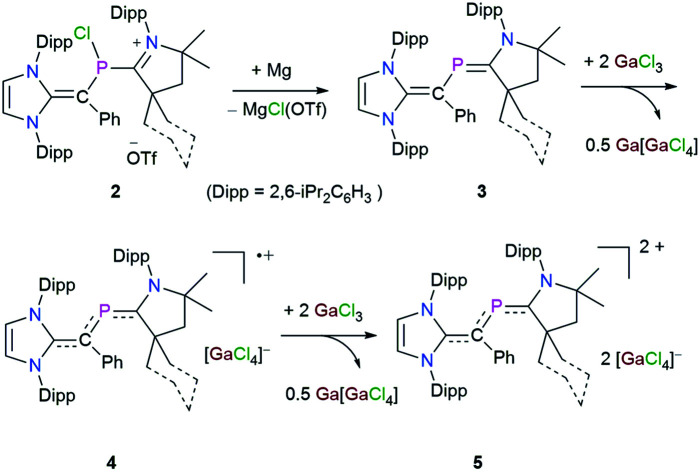

## Introduction

Organic π-conjugated molecules are currently of great academic and significant technological interest due to their intriguing optoelectronic properties.^1^ In this context, π-conjugated systems featuring heavier main-group elements^2^ and systems exhibiting a considerable open-shell (radical-type) character^3^ are particularly attractive as they display promising optical, electronic, and magnetic properties. Among heavier main-group elements, the choice to incorporate phosphorus into π-conjugated systems has been primarily driven by its semblance to the isoelectronic “CR” (R = H, alkyl or aryl group) unit, which is known as a diagonal relationship.^4^ Moreover, while the calculated P=C π-bond strength (43 kcal mol^−1^) is lower than the C=C π-bond of ethene (65 kcal mol^−1^)^5^ the conjugative properties of both P=C and C=C bonds are comparable.^6^

**Fig. 1:**
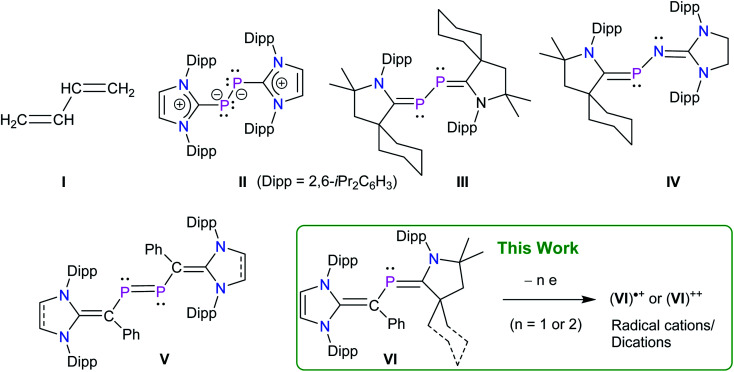
1,3-Butadiene **I**. Selected examples of phosphorus containing derivatives **II–IV** and divinyldiphosphene **V** with singlet carbene frameworks.

Among stable main-group radicals,^7^ various neutral,^8^ cationic,^9^ as well as anionic^10^ phosphorus radicals have been also isolated and structurally characterized, however, phosphorus radicals based on a π-conjugated framework remain scarce. 1,3-Butadiene **I** is the simplest molecule with conjugated π-bonds (Fig. 1) that has also been an important structural motif in phosphorus chemistry.^11^ Indeed, unsubstituted as well as alkyl substituted phosphorus containing 1,3-butadiene derivatives were already reported by Appel,^11*b*^ Regitz,^11*f*^ and Denis,^11*g*^ however, these compounds are unlikely to afford stable radical compounds on oxidation or reduction. In 2008, Robinson *et al.* reported a diphosphorus compound **II** containing a weak π-acceptor N-heterocyclic carbene (NHC).^12^ Structural and theoretical data suggest that **II** should be better described as a base-stabilized diphosphinidene with C_(NHC)_–P and P–P single bonds. Compound **III**, reported by Bertrand's group in 2010, features a strong π-acceptor cyclic alkyl amino carbene (cAAC^R^) and exhibits short C–P bond lengths, thus it may be regarded as a genuine 2,3-diphospha-1,3-butadiene.^13^ The same group also reported the 2-phospha-3-azabutadiene **IV** by an elegant choice of imine and cAAC precursors.^9*c*^ Remarkably, these electron-rich species readily undergo one-electron oxidation to afford the corresponding radical cations (**II**)˙^+^, (**III**)˙^+^, and (**IV**)˙^+^.^9*b*,9*c*^ We recently reported NHC-derived divinyldiphosphenes **V**^14^ and isolated the corresponding radicals cations (**V**)˙^+^ by one-electron oxidation of **V**.^15^ These and other early results^16^ prompted us to reason that stable 2-phospha-1,3-butadienes **VI** as well as the corresponding radical cations (**VI**)˙^+^ and dications (**VI**)^++^ should be synthetically accessible by a rational choice of substrates and reaction conditions.

Herein, we report the synthesis of 2-phospha-1,3-butadienes [{(IPr)C(Ph)}P(cAAC^Me^)] (**3a**) and [{(IPr)C(Ph)}P(cAAC^Cy^)] (**3b**) based on singlet carbene frameworks (IPr = C{(NDipp)CH}_2_, Dipp = 2,6-iPr_2_C_6_H_3_; cAAC^Me^ = C{(NDipp)CMe_2_CH_2_CMe_2_}; cAAC^Cy^ = C{(NDipp)CMe_2_CH_2_C(Cy)}, Cy = cyclohexyl) as crystalline solids. Sequential one-electron oxidation of **3a** and **3b** leads to the formation of corresponding radical cations [{(IPr)C(Ph)}P(cAAC^Me^)](GaCl_4_) (**4a**), [{(IPr)C(Ph)}P(cAAC^Cy^)](GaCl_4_) (**4b**) and dications [{(IPr)C(Ph)}P(cAAC^Me^)](GaCl_4_)_2_ (**5a**), [{(IPr)C(Ph)}P(cAAC^Cy^)] (GaCl_4_)_2_ (**5b**) as crystalline solids.

## Results and discussion

**Scheme 1:**
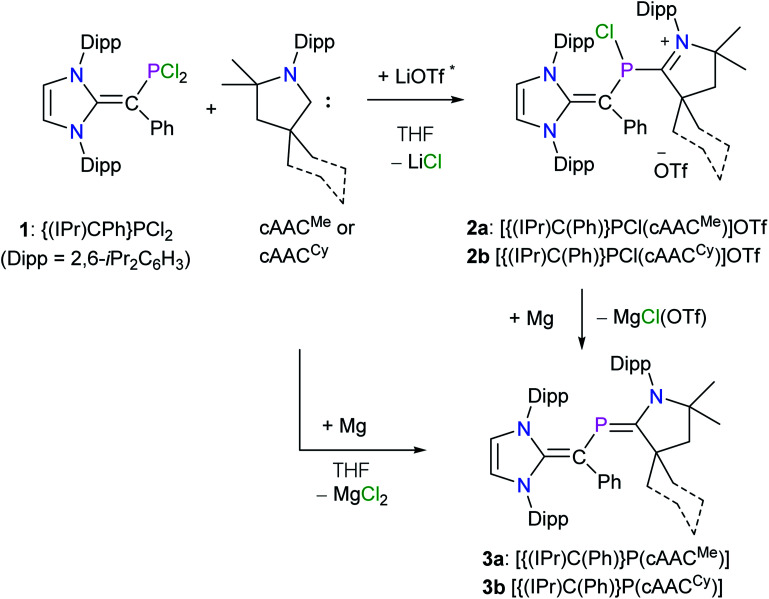
Synthesis of 2-phospha-1,3-butadiene derivatives **3a** and **3b**. * cAACs were prepared by the deprotonation of their triflate salts with LDA and the side-product LiOTf was not separated.

For the synthesis of desired 2-phospha-1,3-butadienes, N-heterocyclic vinyl (NHV)-substituted dichlorophosphine {(IPr)C(Ph)}PCl_2_ (**1**)^14^ and strong π-acceptor cAAC^R^ (ref. 17) were chosen as the appropriate precursors (Scheme 1).^18^ Treatment of a colorless THF solution of **1** with one equivalent of cAAC^Me^ or cAAC^Cy^ immediately resulted in the formation of dark blue solutions (Scheme 1). After workup, the ionic compounds [{(IPr)C(Ph)}P(Cl)(cAAC^R^)](OTf) (R = Me **2a**, Cy **2b**) were isolated as violet crystalline solids. Compounds **2a** and **2b** are highly air sensitive solids and have been characterized by elemental analysis and NMR spectroscopy. The solid state molecular structure of a typical compound **2a** (Fig. S31†) was determined by X-ray diffraction. Reduction of **2a** and **2b** with magnesium turnings afforded the target compound **3a** and **3b**, respectively, as orange solids. Interestingly, **3a** and **3b** are also accessible in a one-pot reaction of **1** and cAAC^R^ with magnesium. Both **3a** and **3b** are soluble in common organic solvents (*n*-hexane, Et_2_O, benzene, toluene, THF) and are stable under an inert gas atmosphere.

The ^1^H NMR spectra of compounds **2a** and **2b** as well as **3a** and **3b** show expected resonances for the NHV and cAAC^R^ moieties. The ^13^C{^1^H} NMR spectrum of **2a** and **2b** as well as **3a** and **3b** each is consistent with the ^1^H NMR resonances and exhibits expected doublets for the phosphorus bound carbon atoms (see the ESI†). The ^31^P{^1^H} NMR spectrum of **2a** (+100.9 ppm) and **2b** (+102.9 ppm) each shows a singlet, which is high-field shifted with respect to that of the **1** (+167 ppm). This is most likely due to the coordination of electron-rich cAAC^R^ to the phosphorus atoms in **2a** and **2b**. The ^31^P{^1^H} NMR signal for **3a** (+102.5 ppm) and **3b** (+108.6 ppm), respectively, appears at a higher field compared to that of **IV** (+134.0 ppm),^9*c*^ which is expected because of the electronegativity difference between carbon and nitrogen.

**Fig. 2:**
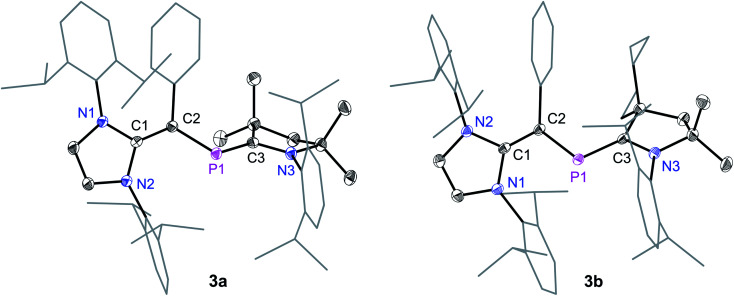
Solid-state molecular structures of 2-phospha-1,3-butadienes **3a** and **3b**. Hydrogen atoms have been omitted for clarity. Selected bond lengths and bond angles are given in Table 1.

The solid-state molecular structures of **3a** and **3b** (Fig. 2) adopt a *trans*-bent geometry along the C_NHV_–P bond with the C2–P1 bond length of 1.818(1) and 1.820(1) Å, respectively. The C2–P1 bond length is larger compared to that in **1** (1.728(2) Å)^14^ and **2a** (1.751(2) Å), but it is comparable with those of the diphosphenes **V** (1.785 to 1.797 Å).^15^ The P1–C3 (**3a**: 1.735(1); **3b** 1.735(1) Å) and C1–C2 (**3a**: 1.386(1); **3b**: 1.384(2) Å) bonds are shorter compared to the same bonds in **2a** (1.860(2) and 1.437(2) Å, respectively). The P1–C3 bond lengths of **3a** and **3b** are nonetheless in line with those of the P=C double bonds in **III** (1.719(7) Å)^13^ and **IV** (1.719(2) Å).^9*c*^ The C1=C2, P1=C3, and P1–C2 bond lengths of **3a** and **3b** are in line with those of the literature known neutral 2-phospha-1,3-butadiene (Me_3_SiO)*t*BuC=P–C(SiMe_3_)=C(OSiiPr_3_)*t*Bu (C=C: 1.356(4), P=C: 1.702(3), P–C: 1.846(3) Å).^19^

**Fig. 3:**
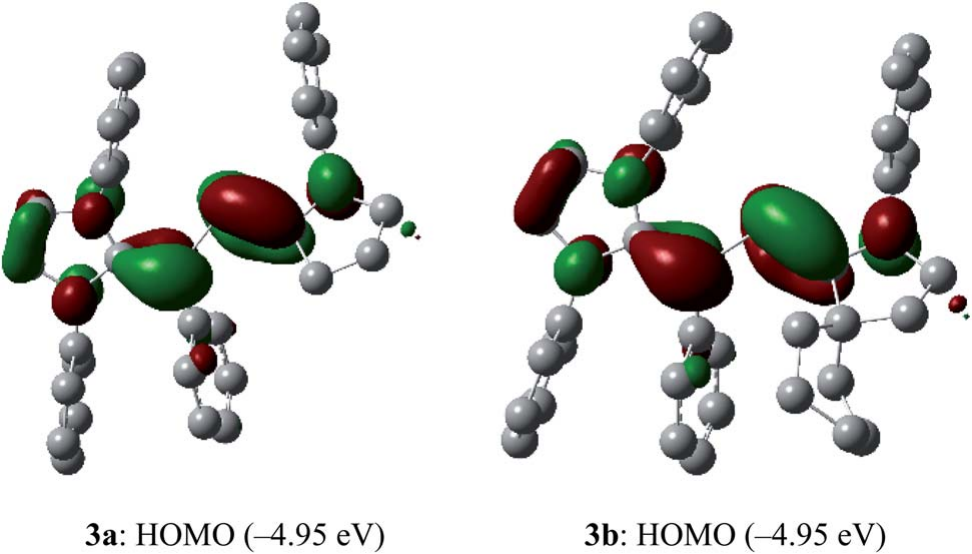
HOMOs (highest occupied molecular orbitals, isovalue 0.04) of **3a** and **3b** calculated at M06-2X/def2-TZVPP//def2-SVP level of theory. Hydrogen atoms, methyl as well as iso-propyl groups were omitted for clarity.

To shed light on the electronic structure of **3a** and **3b**, we performed DFT calculations at the M06-2X/def2-TZVPP//def2-SVP level of theory. The HOMO of **3a** and **3b** is a π-type orbital mainly located at the C_cAAC_=P and C_IPr_=C_Ph_ bonds (Fig. 3). Remarkably, the HOMO energy of **3a** and **3b** (−4.95 eV) is quite high and comparable with those of related divinyldiphosphenes **V** (−4.71 to −5.25 eV),^15^ indicating the possibility of facile oxidation.

**Scheme 2:**
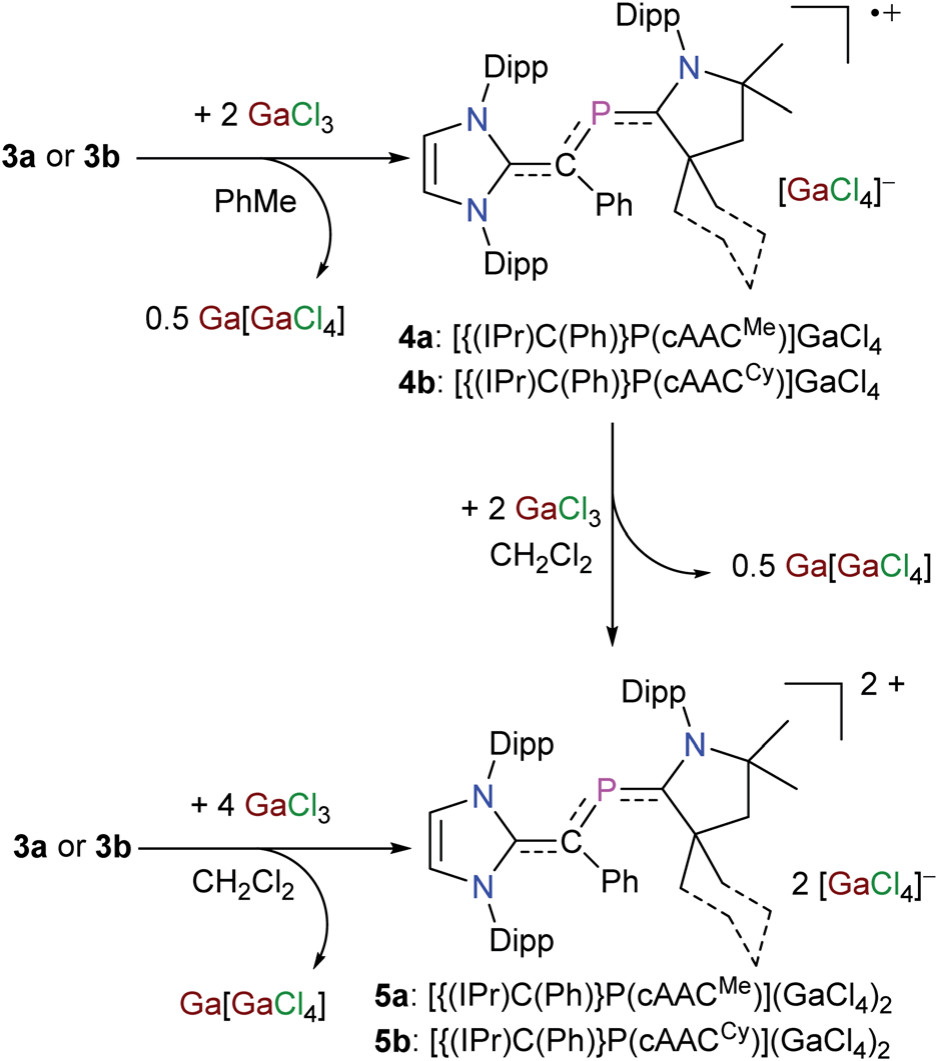
Sequential one-electron oxidation of 2-phospha-1,3-butadienes **3a** and **3b** with GaCl_3_ to the corresponding radical cations **4a** and **4b** and dications **5a** and **5b**.

These preliminarily theoretical findings encouraged us to analyze the redox properties of **3a** and **3b** by electrochemical studies to gain an initial insight into the viability and stability of derived radicals. The cyclic voltammograms (CVs) of **3a** (Fig. S19†) and **3b** (Fig. S20†) show two main redox events in the −2.0 to 1.5 V region. The first reversible wave at *E*_1/2_ = −1.06 V for **3a** and −1.08 V for **3b** may be assigned to the corresponding radical cation, whereas the second quasi-reversible wave at *E*_1/2_ = −0.28 V for **3a** and −0.24 V for **3b** may correspond to the dicationic species. Indeed, treatment of an orange toluene solution of **3a** or **3b** with GaCl_3_ immediately led to the precipitation of a violet solid. After workup, the radical cation salts [{(IPr)C(Ph)}P(cAAC^Me^)](GaCl_4_) (**4a**) and [{(IPr)C(Ph)}P(cAAC^Cy^)](GaCl_4_) (**4b**) were isolated as violet crystals (Scheme 2). GaCl_3_ acts as oxidizing agent and two molecules of GaCl_3_ are required for one-electron oxidation.^15^ Consistent with the CVs (Fig. S19 and S20†), the dication salts [{(IPr)C(Ph)}P(cAAC^Me^)](GaCl_4_)_2_ (**5a**) and [{(IPr)C(Ph)}P(cAAC^Cy^)](GaCl_4_)_2_ (**5b**) are selectively accessible on one-electron oxidation of **4a** and **4b** with GaCl_3_ (Scheme 2). Alternatively, **5a** and **5b** can also be prepared directly from **3a** and **3b** with four equivalent of GaCl_3_, respectively (Scheme 2). Compounds **4a**, **4b** and **5a**, **5b** are stable both in solutions as well as in the solid-state under an inert gas atmosphere, but decompose rapidly when exposed to air. The radicals **4a** and **4b** are NMR silent, while dicationic salts **5a** and **5b** are diamagnetic and exhibit well resolved ^1^H and ^13^C{^1^H} NMR signals for the NHV and cAAC^R^ units. The ^31^P{^1^H} NMR signal for the dication salts **5a** (+244 ppm) and **5b** (+236 ppm) is downfield-shifted with respect to that of the 2-phospha-1,3-butadienes **3a** (+102 ppm) and **3b** (+108 ppm) but it is in the range expected for phosphaalkenes (200–300 ppm).^20^

**Fig. 4:**
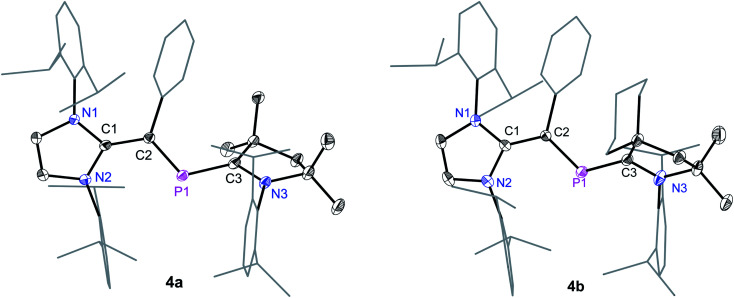
Solid-state molecular structures of radical cation **4a** and **4b**. Hydrogen atoms, solvent molecules in **4b**, and the counter anions GaCl_4_ have been omitted for clarity. Selected bond lengths and bond angles are given in Table 1.

**Fig. 5:**
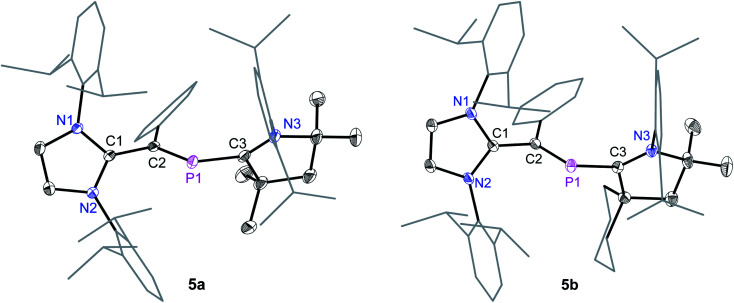
Solid-state molecular structures of **5a** and **5b**. Hydrogen atoms, solvent molecules in **5b**, and the counter anions GaCl_4_ have been omitted for clarity. Selected bond lengths and bond angles are given in Table 1.

Suitable single crystals for X-ray diffraction were obtained by a slow diffusion of *n*-hexane into a saturated THF or CH_2_Cl_2_ solution of each of radical cations **4a** and **4b** and dicationic salts **5a** and **5b**. The solid-state molecular structure of **4a** and **4b** (Fig. 4) as well as **5a** and **5b** (Fig. 5) each adopts a *trans*-bent geometry along the P–C_NHV_ bond and reveals an interesting bond length alteration trend with respect to the precursors **3a** and **3b** (Table 1). The C2–P1 bond length of **4a** (1.758(2) Å) and **4b** (1.761(2) Å) is smaller compared to that of the respective 2-phospha-1,3-butadienes **3a** (1.818(1) Å) and **3b** (1.820(1) Å) but longer with respect to that of the dications **5a** (1.692(3) Å) and **5b** (1.692(2) Å) Å. The P1–C3 bond, however, steadily stretches on going from neutral to radical cations and to dications: **3a**: 1.735(1), **3b**: 1.735(1); **4a**: 1.785(2), **4b**: 1.790(2); **5a**: 1.865(2), **5b**: 1.853(2) Å. A similar trend in the C1–C2 bond length stretching can also be seen in **3a**: 1.386(1), **3b**: 1.384(2); **4a**: 1.432(2), **4b**: 1.433(2); and **5a**: 1.479(3), **5b**: 1.478(2) Å. In conclusion, the formally C1=C2 and P1=C3 double bonds in **3a** and **3b** become comparable to C_sp^2^_–C_sp^2^_ (*ca.* 1.47 Å) and P–C_sp^2^_ (1.85 Å) single bond lengths^21^ in **5a** and **5b**, whereas the C2–P1 single bond in **3a** and **3b** adopts double bond lengths (1.60–1.70 Å) in **5a** and **5b** as expected for phosphaalkenes.^22^

**Table 1:** Selected bond lengths (Å) and angles (°) of 2-phospha-1,3-butadienes (**3a** and **3b**), radical cations (**4a** and **4b**) and dication salts (**5a** and **5b**)

	**3a**	**3b**	**4a**	**4b**	**5a**	**5b**
C1–C2	1.386(1)	1.384(2)	1.432(2)	1.433(2)	1.479(3)	1.478(2)
C2–P1	1.818(1)	1.820(1)	1.758(2)	1.761(2)	1.692(2)	1.692(2)
P1–C3	1.735(1)	1.735(1)	1.785(2)	1.790(2)	1.865(2)	1.853(2)
C1–N1	1.401(1)	1.403(1)	1.375(2)	1.375(2)	1.351(3)	1.344(2)
C1–N2	1.408(1)	1.412(1)	1.382(2)	1.385(2)	1.355(3)	1.356(2)
C3–N3	1.387(1)	1.388(1)	1.349(2)	1.349(2)	1.295(3)	1.295(3)
C1–C2–P1	117.7(1)	118.0(1)	117.3(1)	117.4(1)	114.9(1)	115.3(1)
C2–P1–C3	109.7(1)	110.9(1)	108.8(1)	110.3(1)	106.1(1)	105.2(1)
P1–C3–N3	117.6(1)	116.4(1)	116.0(1)	115.1(1)	118.1(2)	121.5(1)
N1–C1–N2	103.0(1)	103.3(1)	104.8(1)	104.9(1)	107.2(2)	107.3(2)

DFT calculated geometries of **3–5** are found to be fully in agreement with their solid-state molecular structures determined by X-ray diffraction (Table S5†). An increasing value of the WBIs (Wiberg Bond Indices) for the C_NHV_–P bond of **3a** (0.95), **3b** (0.95), **4a** (1.22), **4b** (1.22), **5a** (1.63), and **5b** (1.63) is consistent with the experimental C2–P1 bond lengths (Table 1). Similarly, the WBIs for the P–C_cAAC_ (**3a**: 1.57, **3b**: 1.56; **4a**: 1.19, **4b**: 1.18; **5a**: 0.92, **5b**: 0.91) as well as C–C_Ph_ (**3a**: 1.49, **3b**: 1.49; **4a**: 1.20, **4b**: 1.20; **5a**: 1.06, **5b**: 1.06) bonds also exhibit the expected trend. The NPA (Natural Population Analysis) atomic partial charges (Table S5†) calculated using the NBO (Natural Bond Orbital) method indicate that the phosphorous atom in **3a** (0.49*e*), **3b** (0.49*e*), **4a** (0.65*e*), **4b** (0.65*e*), **5a** (0.85*e*), and **5b** (0.85*e*) carries a positive charge. The former IPr carbene carbon atom (C1) also bears a positive charge (**3a** 0.45*e*, **3b** 0.44*e*, **4a** 0.47*e*, **4b** 0.47*e*, **5a** 0.43*e* and **5b** 0.43*e*), which is larger compared to that of the cAAC carbene carbon atom C3 (**3a** −0.08*e*, **3b** −0.08*e*, **4a** 0.04*e*, **4b** 0.04*e*, **5a** 0.23*e* and **5b** 0.24*e*). This is most likely because of the greater π-acceptor property of cAACs compared to the IPr.

**Fig. 6:**
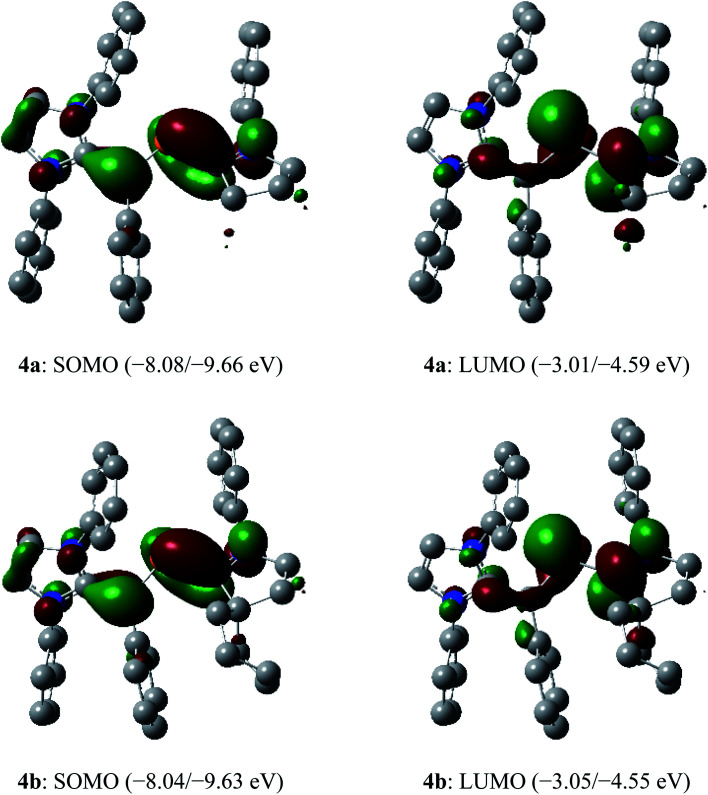
Selected molecular orbitals (isovalue 0.04) of the radical cations **4a** and **4b** (α/β spin orbital energy) calculated at the M06-2X/def2-TZVPP//def2-SVP level of theory. Hydrogen atoms, methyl groups as well as iso-propyl groups were omitted for clarity.

**Fig. 7:**
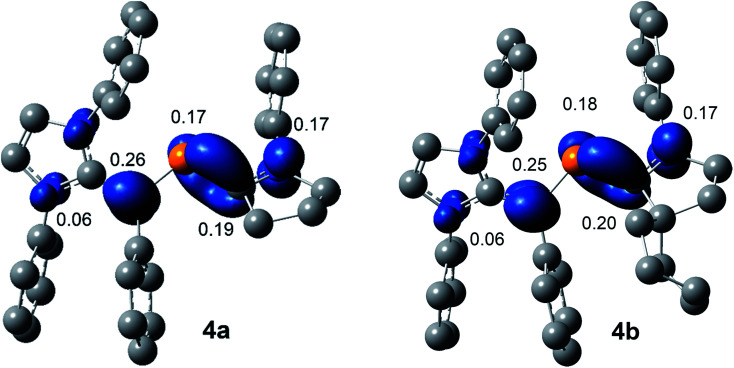
Calculated Mulliken spin densities (isovalue 0.004 a.u.) of radical cations **4a** and **4b** at M06-2X/def2-TZVPP//def2-SVP level of theory.

The SOMO (singly occupied molecular orbital) of **4a** and **4b** (Fig. 6) is the π-orbitals of the C_IPr_=C_vinyl_ and P=C_cAAC_ bonds with a small contribution from the nitrogen atoms of the pyrrolidine and imidazole ring, whereas the LUMO (lowest unoccupied molecular orbital) is the π*-orbitals of the P atom along with the π*-orbitals of C_IPr_=C_vinyl_ and C_cAAC_–N bonds of NHV and cAAC, respectively. The localized Mulliken atomic spin density (Fig. 7) and the plot of the SOMO (Fig. 6) for **4a** and **4b** reveal that the unpaired electron is mainly delocalized over the CPCN moiety with an almost equal spin density distribution on the phosphorus (**4a**: 17%, **4b**: 18%), carbene C_cAAC_ (**4a**: 19%, **4b**: 20%), and vinylic C_Ph_ (**4a**: 26%, **4b**: 25%) atoms. The spin-density at the imidazole-ring nitrogen atoms (6% at each of N) of **4a** and **4b** is rather small compared to that at the pyrrolidine nitrogen atom (17%).

**Fig. 8:**
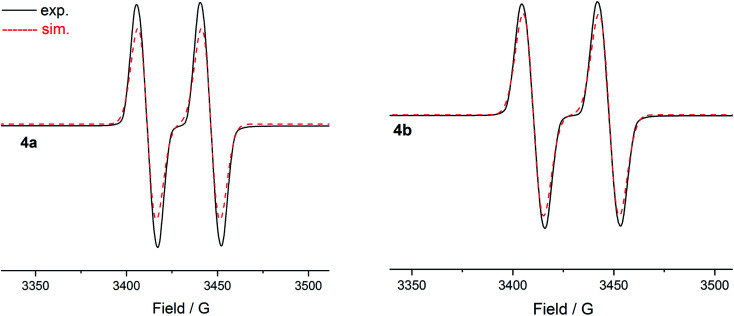
X-band EPR spectra of radical cations **4a** and **4b** in THF at 298 K.

The room temperature X-band EPR spectra of **4a** (*g* = 2.0064) and **4b** (*g* = 2.0064) in THF exhibit a doublet (Fig. 8) owing to the coupling of the unpaired electron with the phosphorous nucleus (*A*_iso_(^31^P) = 35 G **4a**; 38 G **4b**). The magnitude of the hyperfine coupling constant (hfc) of **4a** and **4b** is comparable to those observed for radical cations (**II**)˙^+^ (*A*_iso_(^31^P) = 44 G)^9*b*^ (**III**)˙^+^ (*A*_iso_(^31^P) = 42 G),^9*b*^ and (**IV**)˙^+^ (*A*_iso_(^31^P) = 44 G),^9*c*^ but that is larger than those of (**V**)˙^+^ (*A*_iso_(^31^P) = 12–20 G) featuring a longer π-conjugated system (Fig. 1).^15^ This is, however, considerably smaller than that of the phosphinyl radical cation [(cAAC^Cy^)P(R)]˙^+^ (*A*_iso_(^31^P) = 99 G) (R = 2,2,6,6-tetramethylpiperidino)^9*a*^ as well as those observed for phosphinyl radicals R_2_P˙ (*A*_iso_(^31^P) = 92–96 G) (R = HC(SiMe_3_)_2_ or N(SiMe_3_)_2_),^8*a*,23^ for which, particularly for the latter, the unpaired electron resides predominantly in a 3p(P) valence orbital. The value of coupling constants is in good agreement with the computed values (Table S12†). These hfcs corroborate with the delocalization of the spin-density along the CPCN moiety of **4a** and **4b** (Fig. 7). The measured EPR spectra of **4a** and **4b** were simulated by employing the *g* values, the hyperfine coupling for the phosphorus atom, and two linewidth parameters (Table S13†). The EPR spectra of **4a** (Fig. S29†) and **4b** (Fig. S30†) measured in a frozen THF solution at 80 K show an anisotropic pattern. The *g*-factors (**4a**: *g*_‖_ = 2.0062, *g*_⊥_ = 2.0082; **4b**: *g*_‖_ = 2.0069, *g*_⊥_ = 2.0079) and hfc tensors (**4a**: *A*_*z*_(^31^P) = 149 MHz, *A*_*x*_(^31^P) = −83 MHz, *A*_*y*_(^31^P) = 39 MHz; **4b**: *A*_*z*_(^31^P) = 179 MHz, *A_x_*(^31^P) = −71 MHz, *A*_*y*_(^31^P) = 49 MHz) were determined. The analysis of hfc tensors reveals the major contribution of phosphorus 3p (**4a**: 11.8%; **4b**: 12.8%) orbital to the SOMO, whereas the contribution of the 3s (**4a**: 0.45%; **4b**: 0.46%) orbital is small.

**Fig. 9:**
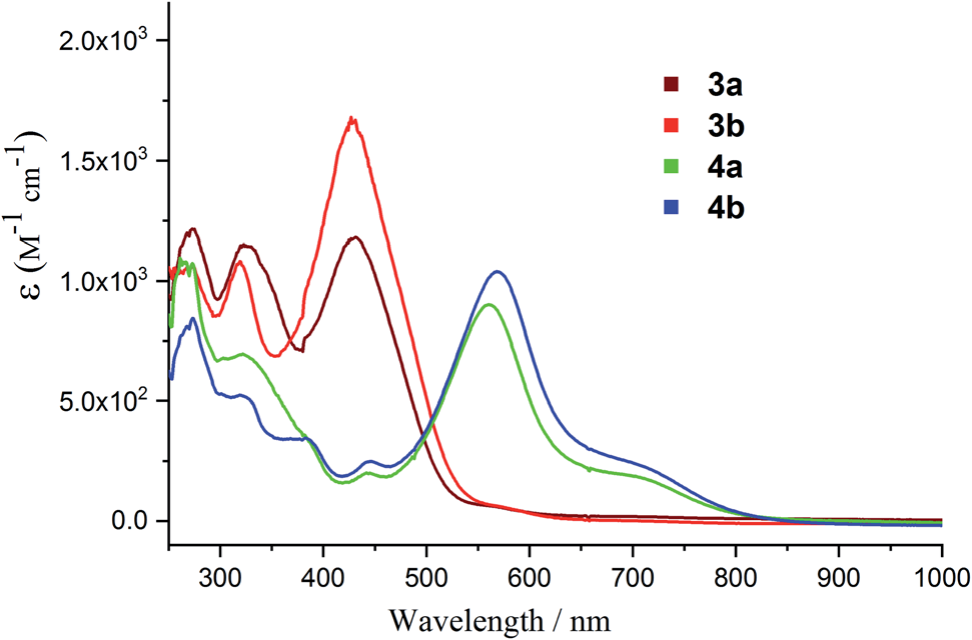
UV-vis spectra of **3a** and **3b** and their radical cations **4a** and **4b** in THF.

The UV-vis spectra of both 2-phospha-1,3-butadienes **3a** (277, 331, 427 nm) and **3b** (271, 322, 430 nm) exhibit three main absorptions (Fig. 9) which, based on TD-DFT calculations at TD-PCM(thf)/M06-2X/def2-SVP level of theory, comprise dominant contributions of the H−1 → L, H → L+2, and H → L transitions, respectively (Fig. S32 and S33, Tables S6 and S7†). The UV-vis spectra of the radical cations **4a** and **4b**, respectively show a broad absorption at 563 and 571 nm along with a shoulder at *ca.* 700 nm (Fig. 9) that corresponds to the SOMO-related transitions S → L and S−1 → S (Tables S8 and S9†).

**Fig. 10:**
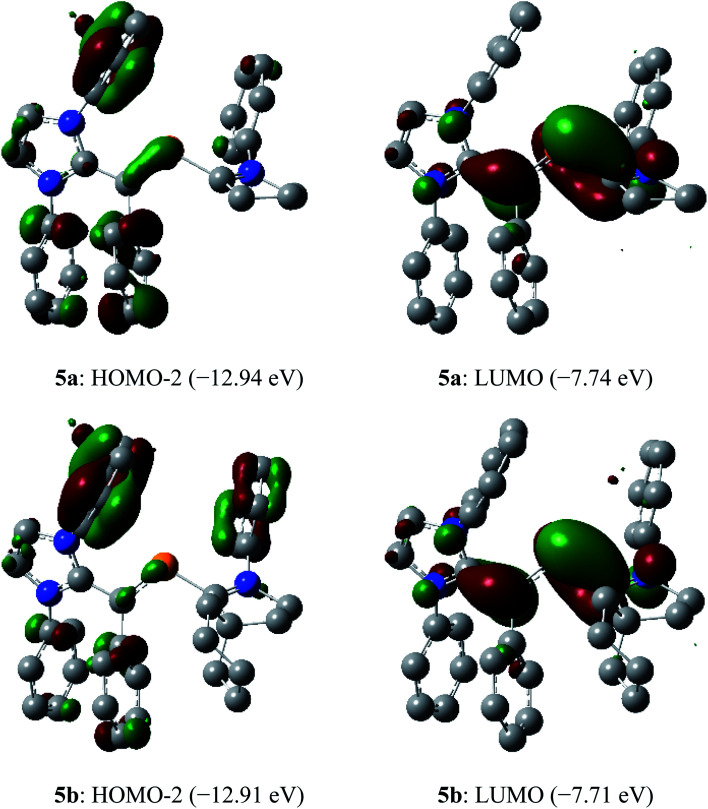
Selected molecular orbitals (isovalue 0.04) of the dications **5a** and **5b** calculated at M06-2X/def2-TZVPP//def2-SVP level of theory. Hydrogen atoms, methyl groups as well as iso-propyl groups were omitted for clarity.

In the dications **5a** and **5b**, the HOMO-2 is a π-type orbital with contributions from the aryl groups and C_vinyl_–P bond (Fig. 10). The LUMO of **5a** and **5b** is the π-orbital of the C_IPr_=C_vinyl_ and P=C_cAAC_ bonds with a small contribution from the nitrogen atoms of pyrrolidine and imidazole rings. Upon removal of one and two electrons from the π-type orbital of **3a** and **3b**, the HOMO of **3a** and **3b** (Fig. 3) becomes SOMO in the radical cations **4a** and **4b** (Fig. 6) and LUMO in the dications **5a** and **5b** (Fig. 10).

## Conclusions

In conclusion, we have isolated the crystalline 2-phospha-1,3-butadiene derivatives **3a** and **3b** by a rational choice of combining a weak π-acceptor (IPr) and a strong π-acceptor (cAAC^R^) singlet carbene scaffolds. Sequential one-electron oxidation of **3a** and **3b** affords the radical cations **4a** and **4b** and the dications **5a** and **5b**. The isolation of **4a**, **4b** and **5a**, **5b** as crystalline solids is consistent with the redox properties of **3a** and **3b** analyzed by electrochemical studies. Molecular structures of all compounds in the solid-state were established by single crystal X-ray diffraction. Computational and EPR spectroscopic data indicate that the unpaired electron in **4a** and **4b** is delocalized over the CPCN π-conjugated framework. The study emphasizes the advantage of merging singlet carbenes with dissimilar donor–acceptor properties in accessing stable open-shell π-conjugated systems. As a variety of stable singlet carbenes with adaptable properties are readily accessible, it is very likely that many π-conjugated systems with other heavier main-group elements, which are hitherto believed to be synthetically challenging targets, may be isolated.

## Conflicts of interest

There are no conflicts to declare.

## Supplementary Material

SC-011-C9SC05598C-s001

SC-011-C9SC05598C-s002
