# Supplementary material for: Isolation of singlet carbene derived 2-phospha-1,3-butadienes and their sequential one-electron oxidation to radical cations and dications
Source: Chem Sci. 2020 Jan 6;11(7):1975–84. doi: 10.1039/c9sc05598c (PMC8148328; doi:10.1039/c9sc05598c)
Supplement: SC-011-C9SC05598C-s001 [file SC-011-C9SC05598C-s001.pdf]

Electronic Supplementary Material (ESI) for Chemical Science.
This journal is © The Royal Society of Chemistry 2020

Electronic Supporting Information

for

Isolation of Singlet Carbenes Derived 2-Phospha-1,3-butadienes and their Sequential One-electron Oxidation to Radical Cations and Dications

Mahendra K. Sharma,[a] Sebastian Blomeyer,[a] Timo Glodde,[a] Beate Neumann,[a] Hans-Georg Stammler,[a] Alexander Hinz,[b] Maurice van Gastel,[c] and Rajendra S. Ghadwal[a]*

[a]*Molecular Inorganic Chemistry and Catalysis, Inorganic and Structural Chemistry, Center for Molecular Materials, Faculty of Chemistry, Universität Bielefeld, Universitätsstrasse 25, D-33615, Bielefeld, Germany.*

[b]*Institute of Inorganic Chemistry, Karlsruhe Institute of Technology (KIT), Engesserstr. 15, 76131 Karlsruhe, Germany.*

[c]*Max-Planck-Institut für Kohlenforschung, Molecular Theory and Spectroscopy, Kaiser-Wilhelm-Platz 1, Mülheim an der Ruhr, D-45470, Germany.*

**Email: rghadwal@uni-bielefeld.de*

https://www.ghadwalgroup.de

Table of contents

Experimental Section	pp 1-5
Plots of the NMR Spectra	pp 6-14
Cyclic Voltammetry	pp 15
UV-vis Spectra	pp 16-19
EPR Spectra	pp 20
Crystallographic Details	pp 21-25
Computational Details	pp 26-52
References	pp 53-54

Experimental Section

All experiments and manipulations were carried out under an inert gas (Ar or N_2) atmosphere using standard *Schlenk* techniques or an MBraun LABmaster Pro glovebox. THF, toluene, and *n*-hexane were dried by refluxing over NaK, distilled prior to use, and stored over 3Å molecular sieve. {(IPr)CPh}PCl_2 (**1**)[1] and cAACs[2] were synthesized by following the literature reported methods. $GaCl_3$ (ABCR) was used as supplied. NMR spectra were recorded using a Bruker Avance III 500HD NMR spectrometer. Chemical shifts are given in δ ppm and referenced to the solvent residual peak(s).[3] Melting points were measured using a Büchi B-545 melting point apparatus. UV-visible spectra were recorded at a Thermo Fisher Evolution 300 spectrophotometer.

Synthesis of [{(IPr)C(Ph)}P(Cl)(cAAC^{Me})](OTf) (2a): To a *Schlenk* flask containing {(IPr)C(Ph)}PCl_2 (**1**) (0.50 g, 0.86 mmol) and cAAC^{Me}(LiOTf) (0.39 g, 0.87 mmol) was added 20 mL THF at room temperature. The resulting violet solution was stirred overnight. The volatiles were removed under vacuum to obtain a dark residue, which was washed with 10 mL toluene and dried, yielding **2a** as a violet solid. Yield: 88%, 0.75 g. M.p. 158 °C (dec.). X-ray quality single crystals were grown by a slow diffusion of *n*-hexane into a saturated dichloromethane solution of **2a**. Elemental analysis (%), calcd for $C_{55}H_{72}N_3ClF_3O_3PS$ (978.67) **2a**: C, 67.50; H, 7.42; N, 4.29; found: C, 68.05; H, 7.93; N, 4.43. ^{1}H NMR (500 MHz, THF-d_8, 298 K): δ = 7.44 (d, J = 7.7 Hz, 1H, NC*H*), 7.38 (d, J = 7.5 Hz, 1H, NC*H*), 7.26–7.32 (m, 7H, C_6H_5, C_6H_3), 7.11 (m, 4H, C_6H_5, C_6H_3), 6.84 (t, J = 7.5 Hz, 2H, C_6H_5), 6.71 (d, J = 7.3 Hz, 1H, C_6H_5), 3.19-3.25 (m, 2H, C*H*$(CH_3)_2$), 3.12-3.18 (m, 1H, C*H*$(CH_3)_2$), 2.87-2.93 (m, 2H, C*H*$(CH_3)_2$), 2.29-2.34 (m, 1H, C*H*$(CH_3)_2$), 2.12-2.20 (q, 2H, CH_2), 1.63 (d, J = 6.4 Hz, 3H, $CH(CH_3)_2$), 1.44 (d, J = 6.7 Hz, 6H, $CH(CH_3)_2$), 1.36 (d, J = 6.4 Hz, 3H, $CH(CH_3)_2$), 1.34 (s, 3H, CH_3), 1.29 (d, J = 6.7 Hz, 6H, $CH(CH_3)_2$), 1.25 (s, 3H, CH_3), 1.22 (m, 6H, $CH(CH_3)_2$), 1.19 (t, J = 6.7 Hz, 3H, $CH(CH_3)_2$), 1.03 (s, 6H, $CH(CH_3)_2$), 1.01 (s, 3H, CH_3), 0.99 (d, J = 7.80 Hz, 3H, $CH(CH_3)_2$), 0.87 (s, 3H, CH_3) ppm. ^{13}C{^{1}H} NMR (125 MHz, THF-d_8, 298 K): δ = 218.0 (d, $J_{P\text{-}C}$ = 93.9 Hz, PC_{cAAC}), 152.6, 152.2, 147.2, 146.3, 145.9, 145.2, 143.7, 136.5, 135.3, 131.8, 131.4, (C_6H_3); 129.2, 129.1, 128.2, 126.9, 126.5, 126.0 125.4, 125.3, 124.0 (C_6H_5); 86.0 (d, $J_{P\text{-}C}$ = 5.7 Hz, CCP), 59.3 ($C(CH_3)_2$), 52.9 (CH_2), 30.7, 30.3, 30.0, 29.8, 29.2, ($CH(CH_3)_2$, 26.8, 24.3, 23.1, 22.0 (CH_3). ^{31}P{^{1}H} NMR (202 MHz, THF-d_8, 298 K): δ = 100.9 ppm. UV-vis (THF, λ (nm) (ε (M^{-1} cm^{-1})): 265 (7560), 326 (12480), 371 (16100), 382 (14800). MS (ESI pos.): *m/z*: 828.5 [**2a**-OTf]$^+$.

Synthesis of [{(IPr)C(Ph)}P(Cl)(cAAC^{Cy})](OTf) (2b): Compound **2b** was synthesized as a violet solid by adopting a similar protocol as described for **2a** using [(IPr)C(Ph)]PCl_2 (1.00 g, 1.73 mmol) and cAAC^{Cy}(LiOTf) (0.83 g, 1.73 mmol). Yield: 83%, 1.45 g. Mp: 167 °C (dec.). Elemental analysis (%), calcd for $C_{58}H_{76}N_3ClF_3O_3PS$ (1018.73) **2b**: C, 68.38; H, 7.52; N, 4.12; found: C, 68.97; H, 7.83; N, 4.31. ^{1}H NMR (500 MHz, THF-d_8, 298 K): δ = 7.43 (t, J = 7.8 Hz, 1H, C_6H_3), 7.37 (s, 2H, NC*H*), 7.35 (t, J = 6.9 Hz, 1H, C_6H_3), 7.31 (d, J = 7.7 Hz, 2H, C_6H_3), 7.27 (d, J = 7.6 Hz, 1H, C_6H_3), 7.23 (d, J =

7.6 Hz, 2H, C_6H_3), 7.16 (m, 3H, C_6H_5), 7.09 (d, J = 7.0 Hz, 2H, C_6H_5), 6.95 (s, 1H), 6.87 (t, J = 7.5 Hz, 2H, C_6H_5), 6.80 (m, 1H, C_6H_3), 6.71-6.74 (m, 1H, C_6H_5), 3.02-3.09 (m, 2H, $CH(CH_3)_2$), 2.95-3.00 (m, 2H, $CH(CH_3)_2$), 2.87-2.93 (m, 1H, $CH(CH_3)_2$), 2.50 (d, J = 14.0 Hz, 1H, CH_2), 2.30-2.35 (m, 1H, $CH(CH_3)_2$), 1.95 (d, J = 13.9 Hz, 1H, CH_2), 1.51-1.58 (m, 4H, CH_2), 1.35-1.39 (m, 6H, CH_2), 1.31 (d, J = 6.9 Hz, 6H, $CH(CH_3)_2$), 1.26 (br, 3H, CH_3), 1.29 (br, 6H, $CH(CH_3)_2$), 1.19 (d, J = 6.6 Hz, 6H, $CH(CH_3)_2$), 1.15 (d, J = 6.8 Hz, 6H, $CH(CH_3)_2$), 1.07 (d, J = 6.7 Hz, 6H, $CH(CH_3)_2$), 1.01 (s, 3H, CH_3) ppm. $^{13}C\{^1H\}$ NMR (125 MHz, THF-d_8, 298 K): δ = 216.6 (d, $J_{P\text{-}C}$ = 95.74 Hz, PC_{cAAC}), 154.1, 153.3, 147.5, 147.3, 145.9, 144.3, 136.1, 135.3, 131.5, 131.1, (C_6H_3); 129.2, 128.3, 127.3, 126.7, 125.8 125.7, 125.6, 125.4, 125.1, 124.6, 121.6 (C_6H_5); 85.5 (d, $J_{P\text{-}C)}$ = 29.8 Hz, CCP), 65.0 ($C(CH_3)_2$, 46.0 (CH_2), 30.8, 30.3, 30.1, 29.9 ($CH(CH_3)_2$, 26.8, 26.5, 23.0, 22.9, 22.7 (CH_3). $^{31}P\{^1H\}$ NMR (202 MHz, THF-d_8, 298 K,): δ = 102.9 ppm. UV-vis (THF, λ (nm) (ε (M^{-1} cm^{-1})): 267 (9100), 325 (7029), 370 (8978). MS (ESI pos.): *m*/*z*: 868.5 [**2b**-OTf]$^+$.

Synthesis of [{(IPr)C(Ph)}P(cAAC^{Me})] (3a): To a *Schlenk* flask containing **2a** (0.50 g, 0.51 mmol) and Mg turnings (25 mg, 1.02 mmol) was added 20 mL THF at room temperature. The resulting reaction mixture was stirred overnight. During this period, the initially violet solution turned into orange. The volatiles were removed in vacuo, giving an orange residue, which was extracted with 50 mL *n*-hexane and filtered through a plug of Celite. The volatiles were removed under vacuum to obtain compound **3a** as an orange solid. Yield: 0.38 g, 94%. M.p. 108 °C (dec.). X-ray quality single crystals were grown by storing a saturated *n*-pentane solution of **3a** at –40 °C for 3 days. Elem. Anal. calcd. for $C_{54}H_{72}N_3P$ (794.14) **3a**: C, 81.67; H, 9.14; N, 5.29, found: C, 82.05; H, 9.51; N, 5.63. ^1H NMR (500 MHz, C_6D_6, 298 K): δ = 7.21 (br, 2H, C_6H_3), 7.14 (d, J = 7.0 Hz, 2H, C_6H_3), 7.10 (d, J = 7.3 Hz, 4H, C_6H_3), 6.95 (br, 4H, C_6H_3, C_6H_5), 6.74 (t, J = 7.4 Hz, 2H, C_6H_5), 6.61 (t, J = 7.2 Hz, 1H, C_6H_5), 3.53 (br, 4H, $CH(CH_3)_2$), 2.88-2.93 (m, 2H, $CH(CH_3)_2$), 1.71 (s, 2H, CH_2), 1.32 (d, J = 6.5 Hz, 12H, $CH(CH_3)_2$), 1.22 (br, 6H, $CH(CH_3)_2$), 1.21 (d, J = 6.7 Hz, 6H, $CH(CH_3)_2$), 1.15 (br, 12H, $CH(CH_3)_2$), 1.09 (br, 6H, CH_3), 0.96 (s, 6H, CH_3) ppm. $^{13}C\{^1H\}$ NMR (125 MHz, C_6D_6, 298 K): δ = 201.2 (d, $J_{P\text{-}C}$ = 74.9 Hz, PC_{cAAC}), 148.9, 146.5, 146.1, 138.5, 135.7, 133.6 (C_6H_3); 126.8, 124.7, 124.1, 122.9, 119.1 (C_6H_5); 76.6 (d, $J_{P\text{-}C}$ = 48.7 Hz, CCP), 66.0, 58.3 ($C(CH_3)_2$, 48.6 (CH_2) 32.2, 29.6, 29.4, 28.9, 28.7, 28.5 ($CH(CH_3)_2$, 25.7, 24.4, 14.4, 14.3 (CH_3). $^{31}P\{^1H\}$ NMR (202 MHz, C_6D_6, 298 K): δ = 102.5 ppm. UV-vis (THF, λ (nm) (ε (M^{-1} cm^{-1})): 277 (11920), 331 (11356), 427 (11710). MS (ESI pos.): *m*/*z*: 794.5 [**3a**+H]$^+$.

One-pot Synthesis of 3a: To a *Schlenk* flask containing **1** (1.20 g, 2.07 mmol), cAAC^{Me}(LiOTf) (0.92 g, 2.07 mmol) and Mg turnings (84 mg, 3.46 mmol) was added 20 mL THF at room temperature. The resulting reaction mixture was stirred overnight. During this period, the initially violet solution turned into orange. The volatiles were removed in vacuo. The resulting orange residue was extracted with 50 mL *n*-hexane. The filtrate was dried under vacuum to obtain **3a** as an orange solid. Yield: 1.60 g, 97%.

Synthesis of [{(IPr)C(Ph)}P(cAAC^{Cy})] (3b): Compound **3b** was synthesized following the similar protocol as described for compound **3a** using compound **2b** (0.20 g, 0.19 mmol) and Mg turnings (10 mg, 0.39 mmol) as an orange. Yield: 150 mg, 93%. Mp: 117 °C (dec.). Elem. Anal. calcd. for $C_{57}H_{76}N_3P$ (834.21) **3b**: C, 82.07; H, 9.18; N, 5.04, found: C, 82.63; H, 9.67; N, 5.29. ^{1}H NMR (500 MHz, C_6D_6, 298 K): δ = 7.31 (br, 2H, C_6H_3), 7.14-7.21 (m, 2H, C_6H_3), 7.04 (br, 3H, C_6H_3), 6.94 (br, 4H, C_6H_3, C_6H_5), 6.78 (t, *J* = 7.4 Hz, 2H, C_6H_5), 6.58 (t, *J* = 7.1 Hz, 1H, C_6H_5), 3.54 (br, 4H, C*H*(CH_3)$_2$), 2.95-2.98 (m, 2H, C*H*(CH_3)$_2$), 1.75 (s, 2H, CH_2), 1.50 (br, 4H, CH(CH_3)$_2$), 1.32 (br, 12H, CH(CH_3)$_2$), 1.22 (d, *J* = 6.7 Hz, 14H, CH(CH_3)$_2$, CH_2), 1.17 (br, 16H, CH(CH_3)$_2$, CH_2), 0.98 (s, 6H, CH_3) ppm. ^{13}C{^{1}H} NMR (125 MHz, C_6D_6, 298 K): δ = 200.4 (d, $J_{P\text{-}C}$ = 75.3 Hz, PC_{cAAC}), 149.0, 148.5, 146.4, 146.0, 145.7, 138.5, 135.9, 133.5 (*C*$_6$H$_3$); 127.4, 124.8, 124.1, 123.0, 119.4 (*C*$_6$H$_5$); 77.7 (d, $J_{P\text{-}C}$ = 50.3 Hz, C*C*P), 66.1, 54.6, 54.5 (*C*(CH_3)$_2$, 50.1 (*C*H$_2$); 29.4, 29.0, 28.7, 28.3 (CH(*C*H$_3$)$_2$, 25.5, 24.6, 24.1, 22.8, 14.4, 14.3 (*C*H$_3$). ^{31}P{^{1}H} NMR (202 MHz, C_6D_6, 298 K): δ = 108.6 ppm. UV-vis (THF, λ (nm) (ε (M^{-1} cm^{-1})): 271 (11790), 322 (11950), 430 (18546).). MS (ESI pos.): *m*/*z*: 834.6 [**3b**+H]$^+$.

One-pot Synthesis of 3b: To a *Schlenk* flask containing **1** (1.00 g, 1.73 mmol), cAAC^{Cy}(LiOTf) (0.83 g, 1.73 mmol), and Mg turnings (84 mg, 3.46 mmol) was added 20 mL THF at room temperature. The resulting reaction mixture was stirred overnight. During this period, the initially violet solution turned into orange. The volatiles were removed in vacuo, giving an orange residue, which was extracted with 50 mL *n*-hexane and filtered through a plug of Celite. Removal of *n*-hexane from the filtrate under vacuum gave **3b** as an orange solid. Yield: 1.4 g, 97%.

Synthesis of [{(IPr)C(Ph)}P(cAAC^{Me})](GaCl$_4$) (4a): To a 20 mL toluene solution of **3a** (0.2 g, 0.25 mmol) was added $GaCl_3$ (88 mg, 0.50 mmol) in one portion with constant stirring at room temperature. After stirring overnight, the violet precipitate was collected by filtration and dried in *vacuo* to afford **4a** as a violet solid. Yield: 0.23 g, 90%. Mp: 143 °C (dec.). Single crystals suitable for X-ray diffraction were grown by a slow diffusion of *n*-hexane into a saturated THF solution of **4a**. Elem. Anal. calcd. for $C_{54}H_{72}Cl_4GaN_3P$ (1005.68) **4a**: C, 64.49; H, 7.22; N, 4.18, found: C, 64.93; H, 7.65; N, 4.39. UV-vis (THF, λ (nm) (ε (M^{-1} cm^{-1})): 268 (5169), 327 (3408), 446 (1110), 563 (4420).). MS (ESI pos.): *m*/*z*: 794.6 [(**4a**-GaCl$_4$)+H]$^+$, 828.5 [(**4a**-GaCl$_4$)+Cl]$^+$.

Synthesis of [{(IPr)C(Ph)}P(cAAC^{Cy})](GaCl$_4$) (4b): Compound **4b** was synthesized following the similar protocol as described for compound **4a** using compound **3b** (0.20 g, 0.24 mmol) and $GaCl_3$ (84 mg, 0.48 mmol). Yield: 0.20 g, 81%; violet solid. Mp: 137 °C (dec.). Single crystals suitable for X-ray diffraction were grown by a slow diffusion of *n*-hexane into a saturated THF solution of **4b**. Elem. Anal. calcd. for $C_{57}H_{76}Cl_4GaN_3P$ (1045.74) **4b**: C, 65.47; H, 7.33; N, 4.02, found: C, 65.97; H, 7.71; N, 4.33. UV-vis (THF, λ (nm) (ε (M^{-1} cm^{-1})): 275 (9200), 329 (5750), 384 (4031), 448 (3000), 571 (11390). MS (ESI pos.): *m*/*z*: 834.6 [(**4b**-GaCl$_4$)+H]$^+$, 868.6 [(**4b**-GaCl$_4$)+Cl]$^+$.

Synthesis of [{(IPr)C(Ph)}P(cAAC^{Me})](aCl$_4$)$_2$ (5a): To a 10 mL dichloromethane solution of **3a** (0.15 g, 0.15 mmol) was transferred $GaCl_3$ (53 mg, 0.30 mmol) at rt. The color of the solution turned orange to yellow immediately, which was further stirred for 6 h. The volatiles were removed in *vacuo* to afford **5a** as a yellow solid. Yield: 178 mg, 98%. Mp: 143 °C (dec.). Single crystals suitable for X-ray diffraction were grown by a slow diffusion of *n*-hexane into a saturated dichloromethane solution of **5a**. Elem. Anal. calcd. for $C_{54}H_{72}Cl_8Ga_2N_3P$ (1217.21) **5a**: C, 53.28; H, 5.96; N, 3.45; found: C, 53.63; H, 6.41; N, 3.67. ^{1}H NMR (500 MHz, CD_2Cl_2, 298 K): δ = 7.98 (s, 2H, NC*H*), 7.74 (t, *J* = 8.5 Hz, 1H, C_6H_3), 7.70 (d, *J* = 8.5 Hz, 1H, C_6H_3), 7.61 (t, *J* = 7.8 Hz, 2H, C_6H_3), 7.53 (t, *J* = 7.6 Hz, 2H, C_6H_5), 7.48 (d, *J* = 7.8 Hz, 2H, C_6H_3), 7.29 (d, *J* = 7.7 Hz, 2H, C_6H_5), 6.84 (d, *J* = 7.6 Hz, 2H, C_6H_5), 2.55 (br, 2H, C*H*(CH_3)$_2$), 2.35 (s, 2H, CH_2), 2.30 (br, 4H, C*H*(CH_3)$_2$), 1.55 (s, 6H, CH_3), 1.33 (d, *J* = 6.4 Hz, 6H, CH(CH_3)$_2$), 1.16 (d, *J* = 6.6 Hz, 12H, CH(CH_3)$_2$), 1.04 (br, 6H, CH(CH_3)$_2$), 0.93 (d, *J* = 5.2 Hz, 12H, CH(CH_3)$_2$), 0.64 (br, 6H, CH_3) ppm. ^{13}C{^{1}H} NMR (125 MHz, CD_2Cl_2, 298 K): δ = 213.6 (d, $J_{P\text{-}C}$ = 91.0 Hz, PC_{cAAC}), 172.3 (d, $J_{P\text{-}C}$ = 75.3 Hz, PC=*C*), 146.2, 145.2, 144.8, 140.3, 136.0, 134.3, 134.2, 131.4 (C_6H_3); 129.1, 129.0, 128.8, 126.2 (C_6H_5); 87.1 (*C*(CH_3)$_2$), 74.2 (N*C*(CH_3)$_2$), 58.3, 52.6 (*C*H_2); 32.1, 30.6, 30.5, 27.4 (CH(*C*H_3)$_2$, 26.3, 25.8, 23.2, 22.9, 22.3, 14.4 (*C*H_3) ppm. ^{31}P{^{1}H} NMR (202 MHz, CD_2Cl_2, 298 K): δ = 243.9 ppm. UV-vis (THF, λ (nm) (ε (M^{-1} cm^{-1})): 340 (16590), 384 (13620). MS (ESI pos.): *m*/*z*: 794.6 [(**5a**-2GaCl$_4$)+H]$^+$.

Alternate Synthesis of 5a: To a DCM solution (5 mL) of **4a** (50 mg, 0.05 mmol), was transferred $GaCl_3$ (18 mg, 0.10 mmol) at rt. The violet colored solution turned yellow immediately, which was further stirred for 10 min. The volatiles were removed in *vacuo* to get a yellow residue. This was washed with n-hexane (5 mL) and dried to afford **5a** as a yellow solid. Yield (60 mg, 99%).

Synthesis of [{(IPr)C(Ph)}P(cAAC^{Cy})](GaCl$_4$)$_2$ (5b): Compound **5b** was synthesized following the similar protocol as described for compound **5a** using **3b** (30 mg, 0.03 mmol), and $GaCl_3$ (10 mg, 0.06 mmol). Yield (35 mg, 99%; yellow solid). Mp: 149 °C (dec.). Elem. Anal. calcd. for $C_{57}H_{76}Cl_8Ga_2N_3P$ (1257.28) **5b**: C, 54.45; H, 6.09; N, 3.34; found: C, 55.15; H, 6.69; N, 3.54. ^{1}H NMR (500 MHz, CD_2Cl_2, 298 K): δ = 7.95 (s, 2H, NC*H*), 7.76 (t, *J* = 7.5 Hz, 1H, C_6H_3), 7.70 (t, *J* = 8.8 Hz, 1H, C_6H_3), 7.63 (t, *J* = 7.8 Hz, 2H, C_6H_3), 7.54 (t, *J* = 7.6 Hz, 2H, C_6H_5), 7.46 (d, *J* = 7.0 Hz, 2H, C_6H_3), 7.32 (d, *J* = 6.5 Hz, 4H, C_6H_5, C_6H_3), 6.92 (d, *J* = 7.6 Hz, 2H, C_6H_5), 2.23-2.75 (br, 6H, C*H*(CH_3)$_2$), 2.36 (s, 2H, CH_2), 1.39-1.68 (br, 10H, CH_2), 1.33 (d, *J* = 6.2 Hz, 12H, CH(CH_3)$_2$), 1.17 (d, *J* = 6.4 Hz, 12H, CH(CH_3)$_2$), 0.64 (m, 3H, CH_3), 0.45 (br, 3H, CH_3) ppm. ^{13}C{^{1}H} NMR (125 MHz, CD_2Cl_2, 298 K): δ = 212.9 (d, $J_{P\text{-}C}$ = 91.1 Hz, PC_{cAAC}), 171.4 (d, $J_{P\text{-}C}$ = 75.3 Hz, PC=*C*), 145.5, 144.5, 139.6, 136.3, 134.2, 134.2 131.1, 130.8 (C_6H_3); 129.1, 129.0, 128.8, 126.2 (C_6H_5); 87.1 (*C*(CH_3)$_2$), 64.1 (N*C*(CH_3)$_2$), 46.0 (*C*H_2); 30.7, 27.4 (CH(*C*H_3)$_2$, 25.8, 23.5, 22.6, 14.4 (*C*H_3) ppm. ^{31}P{^{1}H} NMR (202 MHz, CD_2Cl_2, 298 K): δ = 236.3 ppm. UV-vis (THF, λ (nm) (ε (M^{-1} cm^{-1})): 329 (12900), 385 (15990). MS (ESI pos.): *m*/*z*: 834.6 [(**5b**-2GaCl$_4$)+H]$^+$.

Alternate Synthesis of 5b: To a DCM solution (5 mL) of **4b** (0.15 g, 0.14 mmol), was transferred $GaCl_3$ (50 mg, 0.28 mmol), at rt. The violet colored solution turned yellow immediately, which was further stirred for 10 min. The volatiles were removed in *vacuo* to get a yellow residue. This was washed with n-hexane (5 mL) and dried to afford **5b** as a yellow solid. Yield (178 mg, 99%).

Plots of the NMR spectra

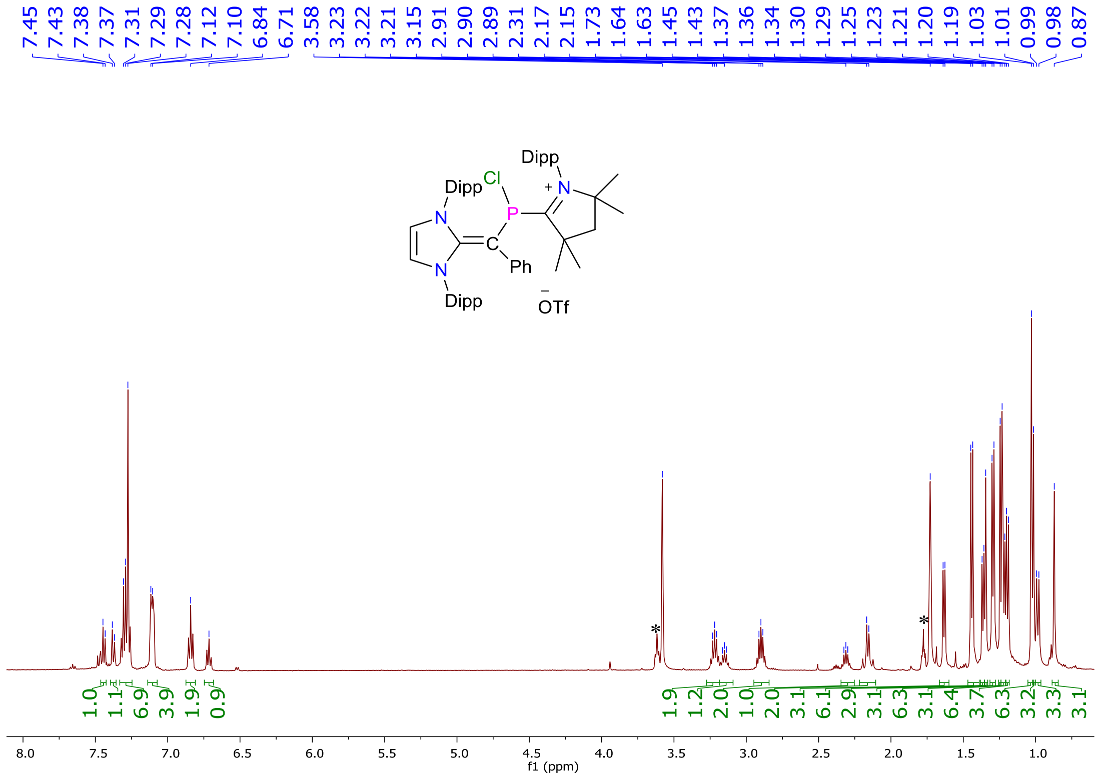

Figure S1. ^{1}H NMR (500 MHz, THF-d_8, 298 K,) spectrum of compound **2a** (* = THF).

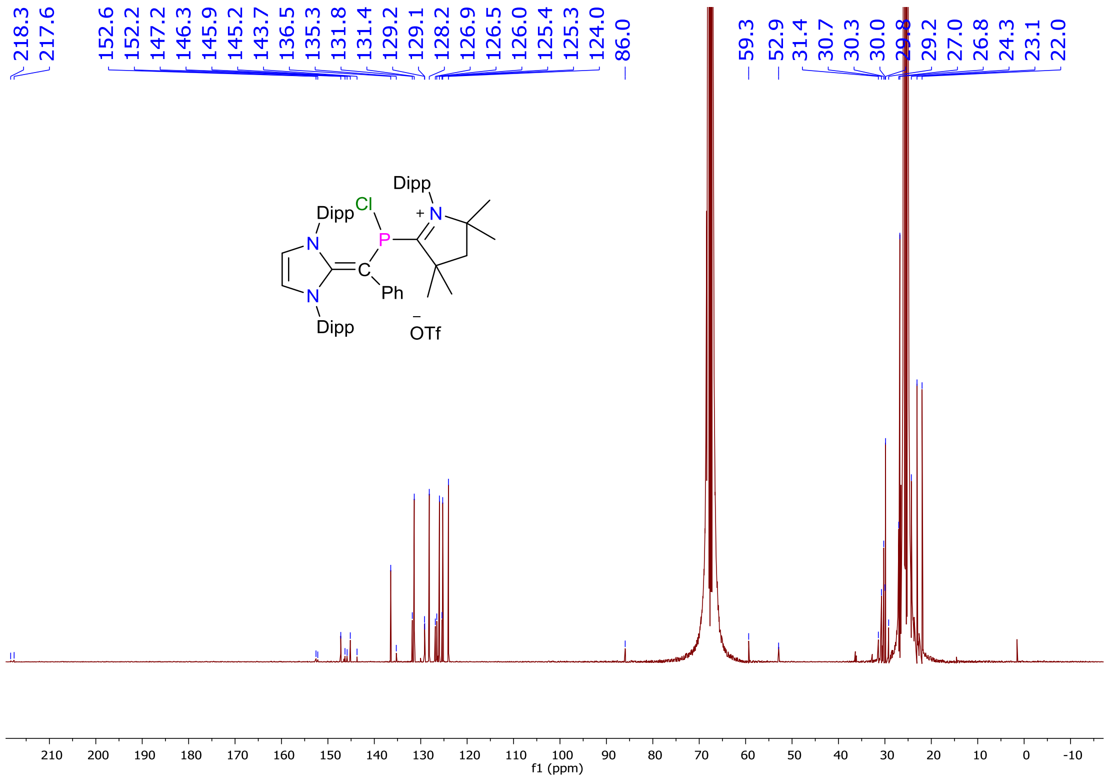

Figure S2 . $^{13}C\{^{1}H\}$ NMR (125 MHz, THF-d_8, 298 K) spectrum of compound **2a**.

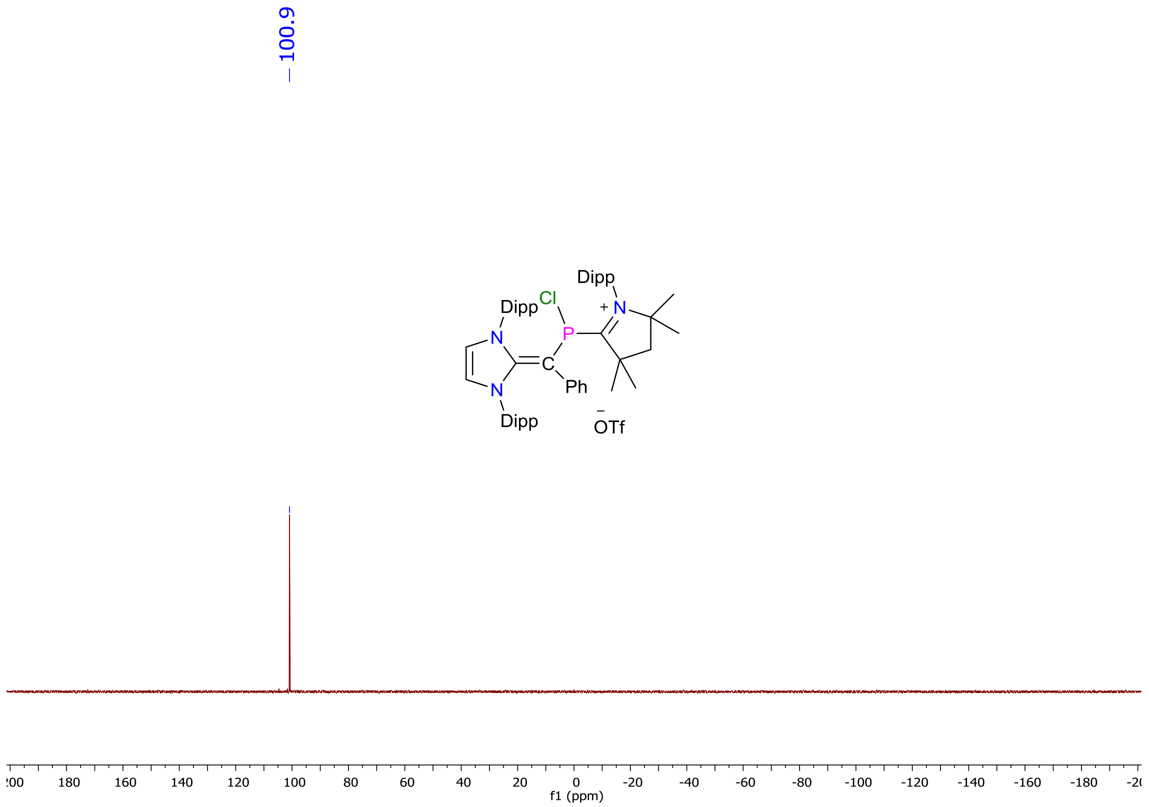

Figure S3. $^{31}P\{^1H\}$ NMR (202 MHz, THF-d_8, 298 K) spectrum of compound **2a**.

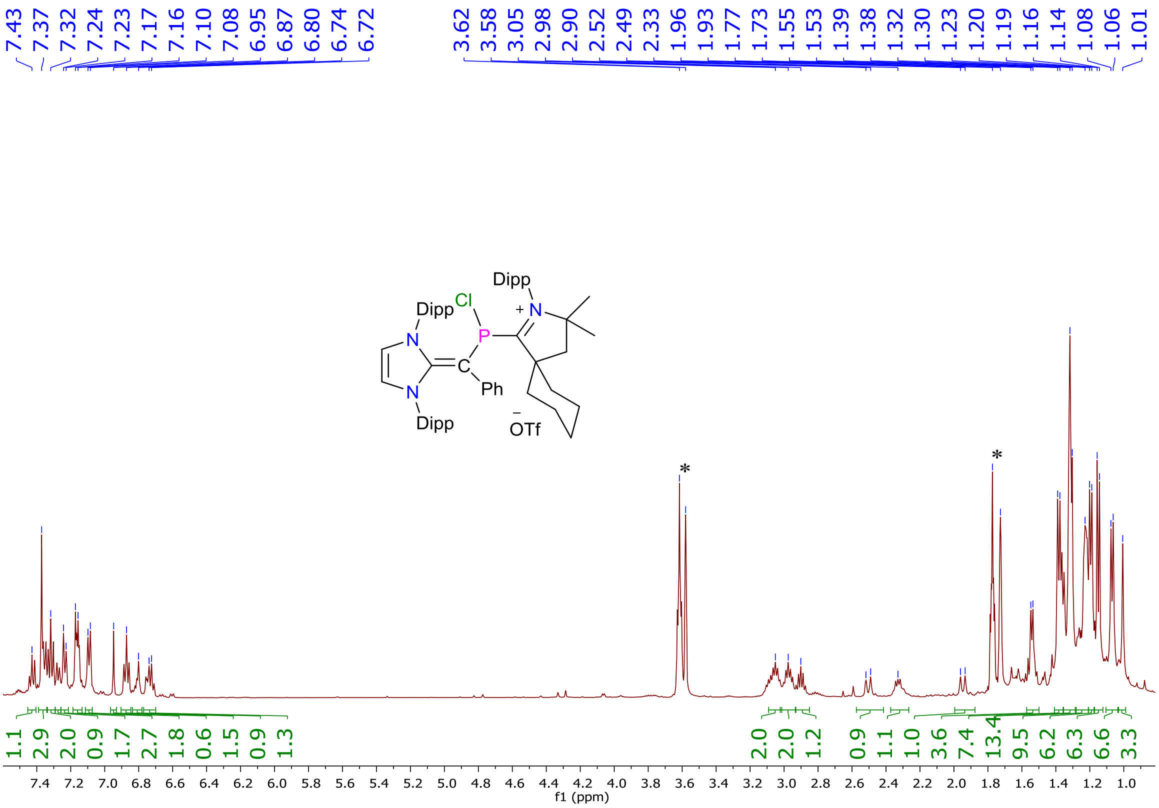

Figure S4. ^1H NMR (500 MHz, THF-d_8, 298 K) spectrum of compound **2b** (* = THF).

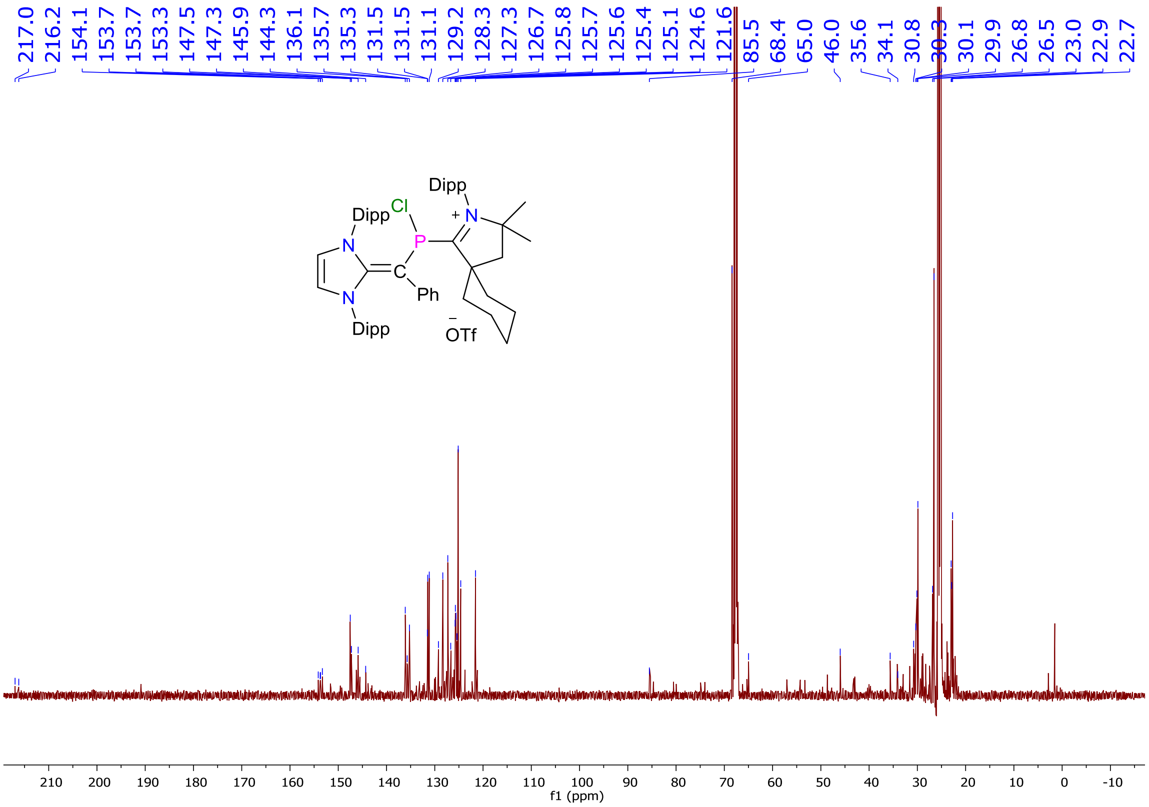

Figure S5. $^{13}C\{^{1}H\}$ NMR (125 MHz, THF-d_8, 298 K,) spectrum of compound **2b**.

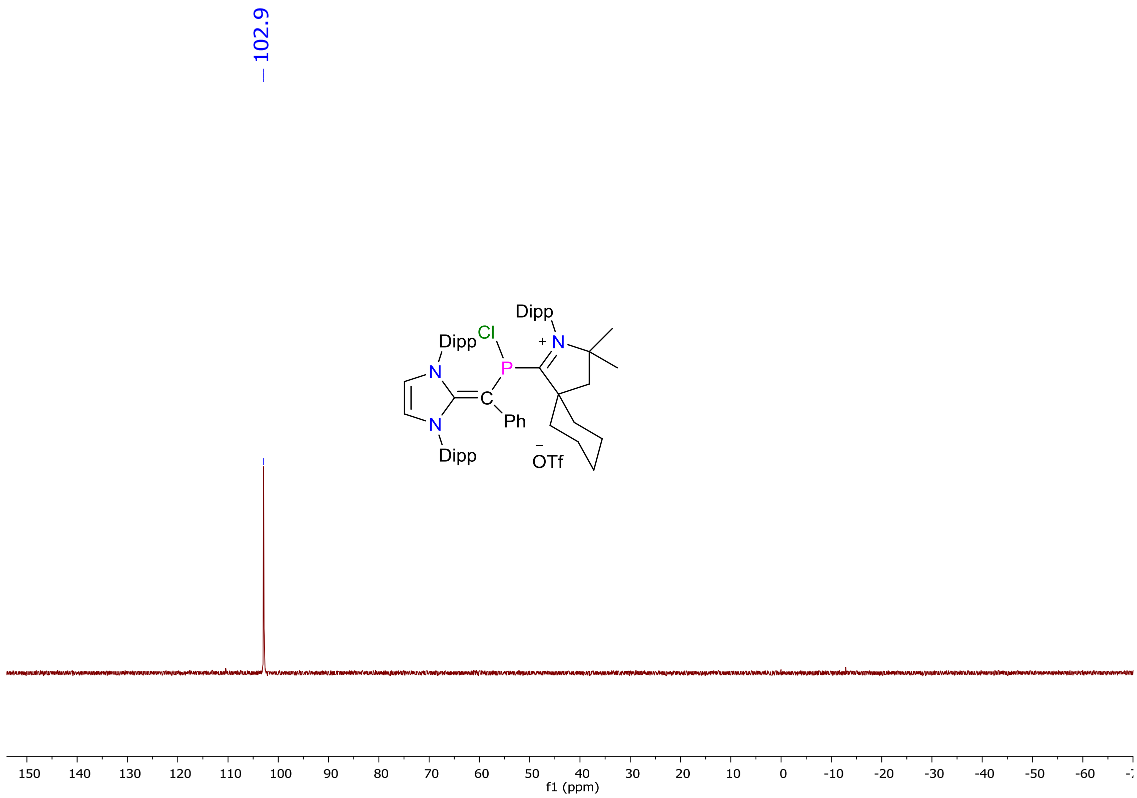

Figure S6. $^{31}P\{^{1}H\}$ NMR (202 MHz, THF-d_8, 298 K) spectrum of compound **2b**.

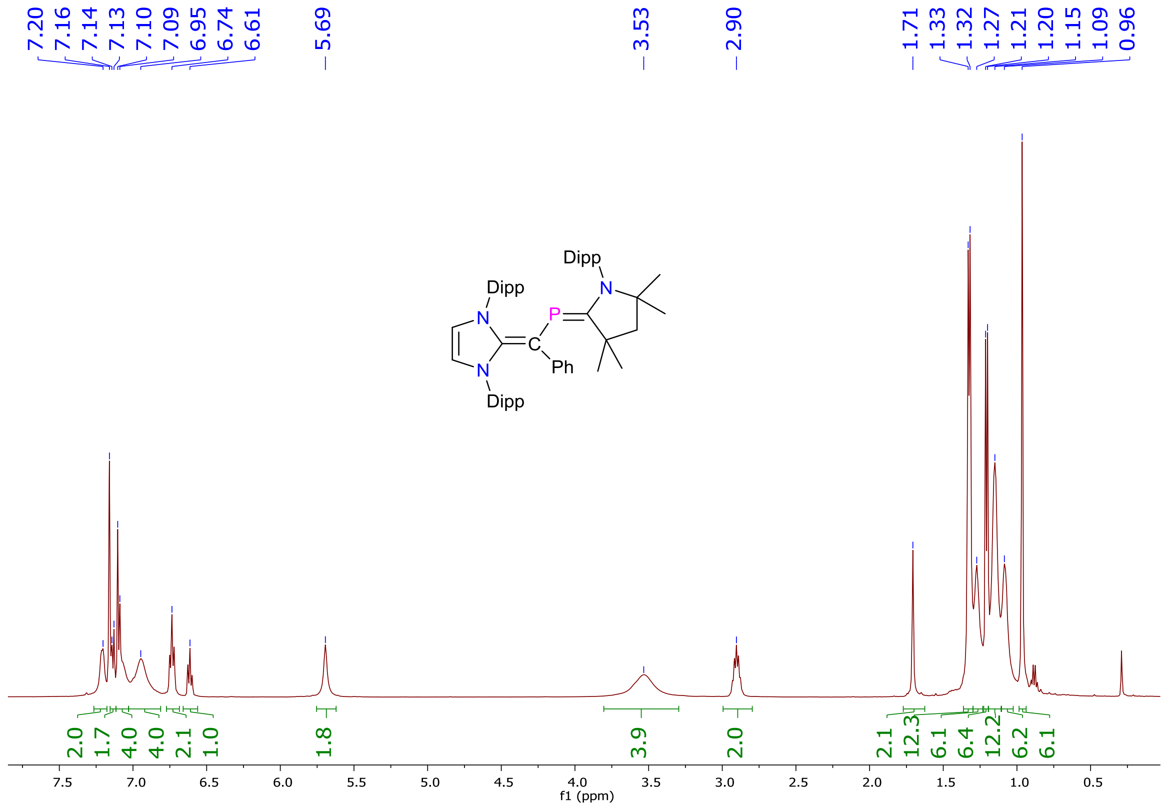

Figure S7. ^{1}H NMR (500 MHz, C_6D_6, 298 K) spectrum of compound **3a**.

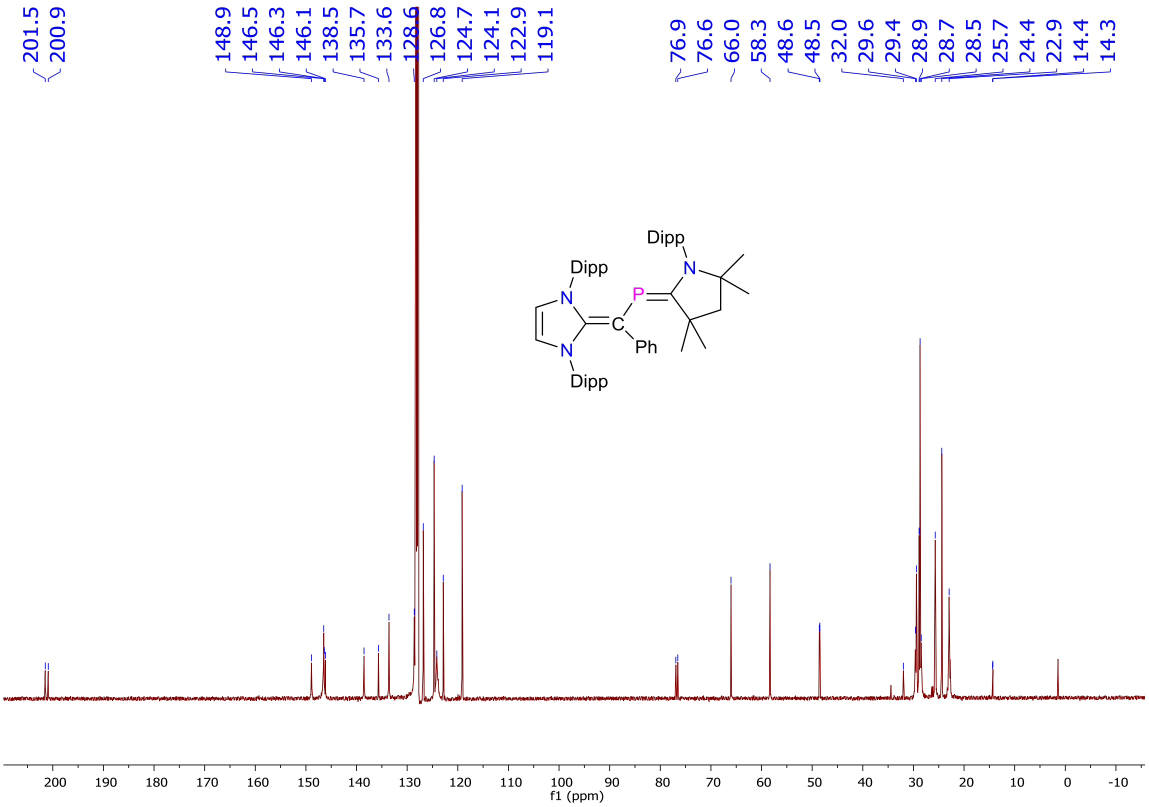

Figure S8. ^{13}C{^{1}H} NMR (125 MHz, C_6D_6, 298 K) spectrum of compound **3a**.

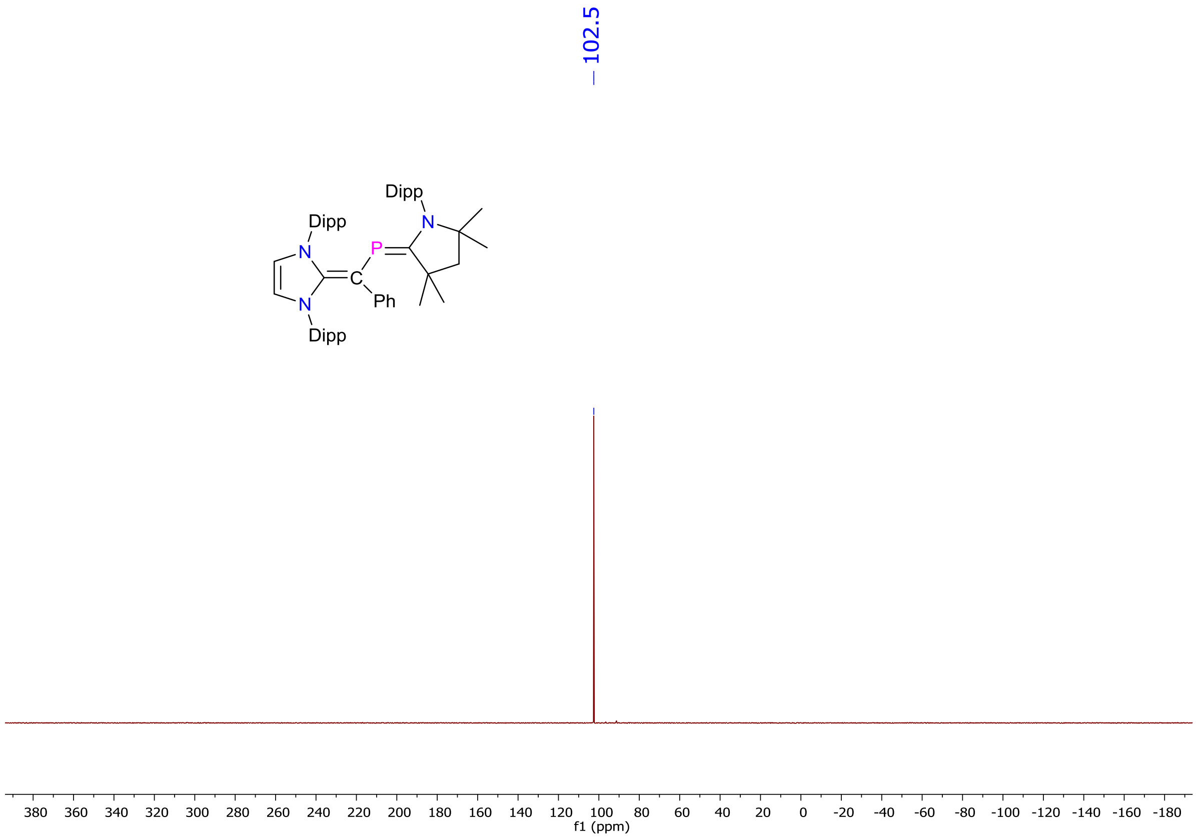

Figure S9. $^{31}P\{^{1}H\}$ NMR (202 MHz, C_6D_6, 298 K) spectrum of compound **3a**.

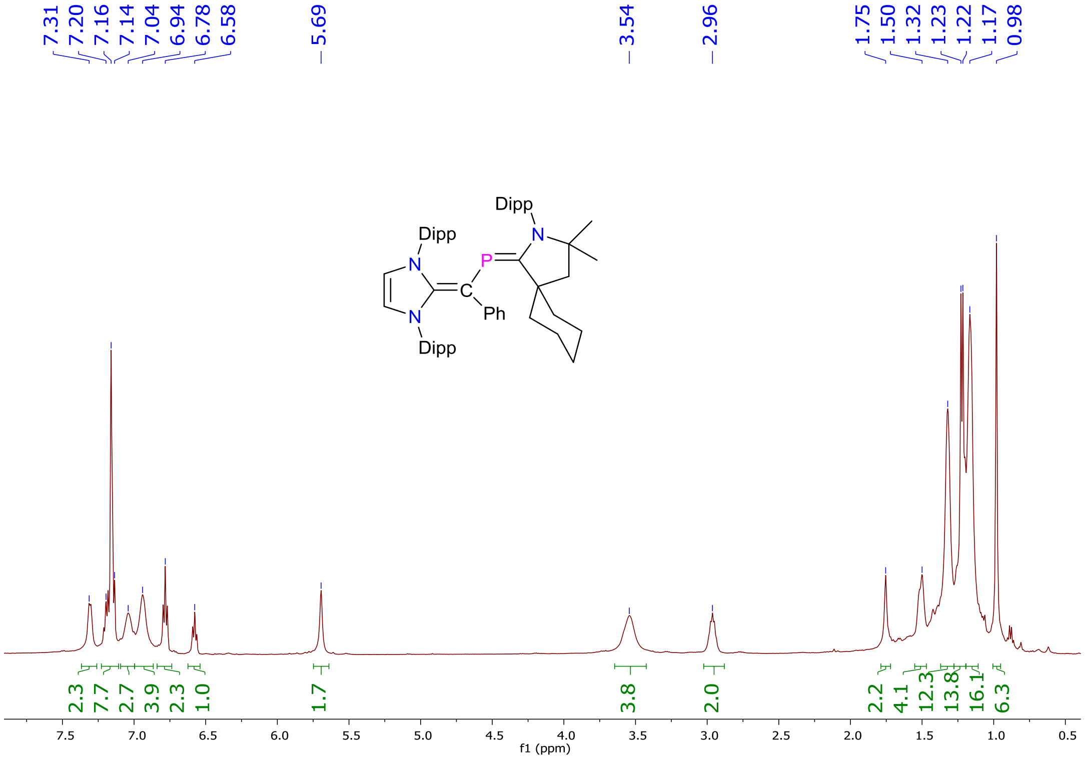

Figure S10. ^{1}H NMR (500 MHz, C_6D_6, 298 K) spectrum of compound **3b**.

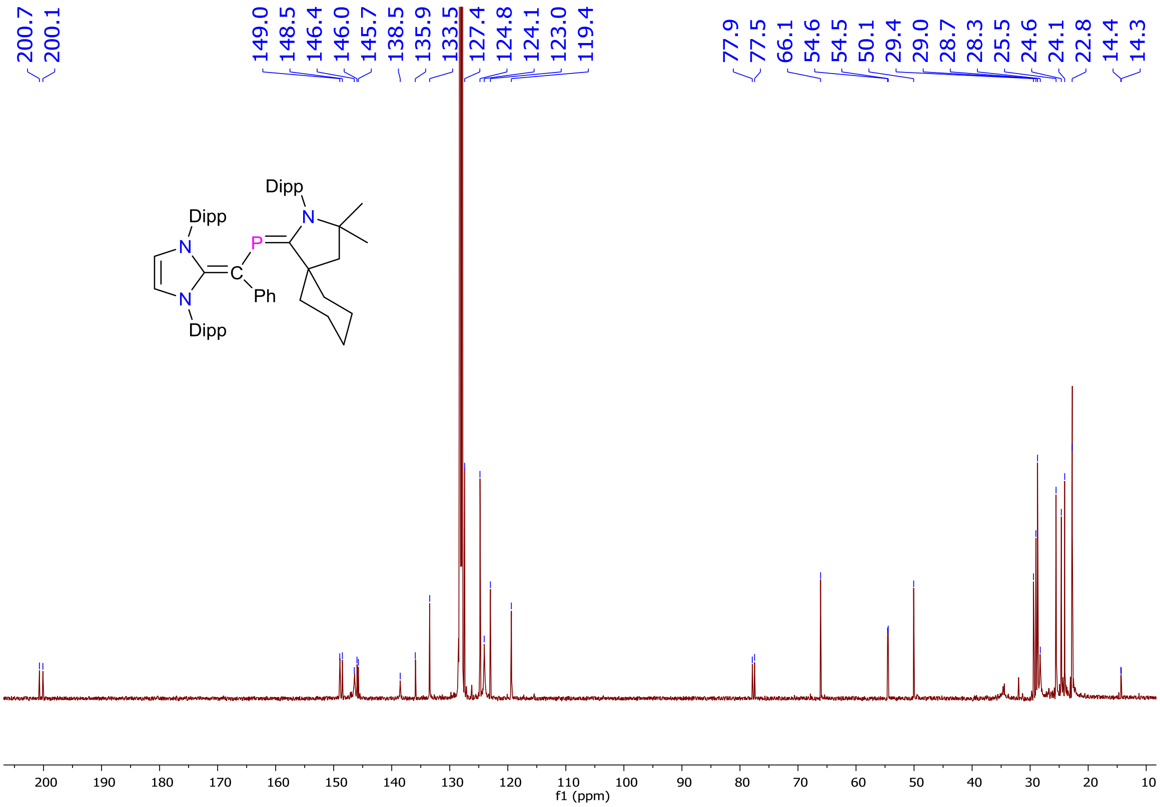

Figure S11. $^{13}C\{^{1}H\}$ NMR (125 MHz, C_6D_6, 298 K) spectrum of compound **3b**.

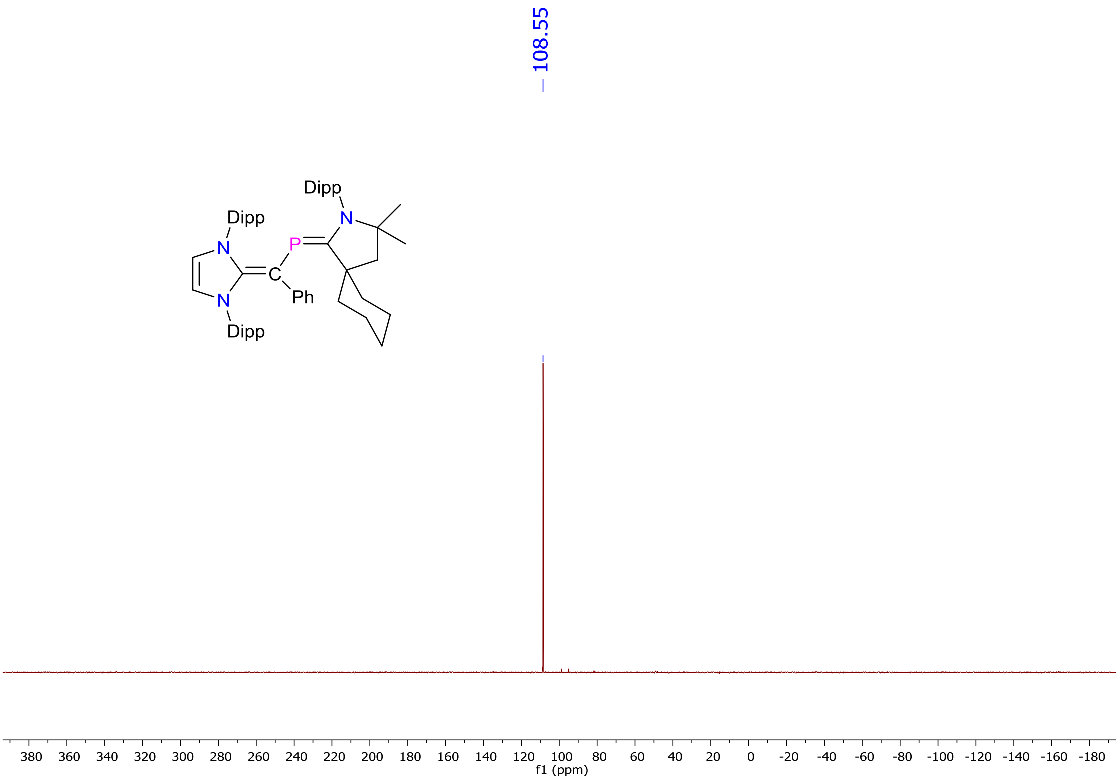

Figure S12. $^{31}P\{^{1}H\}$ NMR (202 MHz, C_6D_6, 298 K) spectrum of compound **3b**.

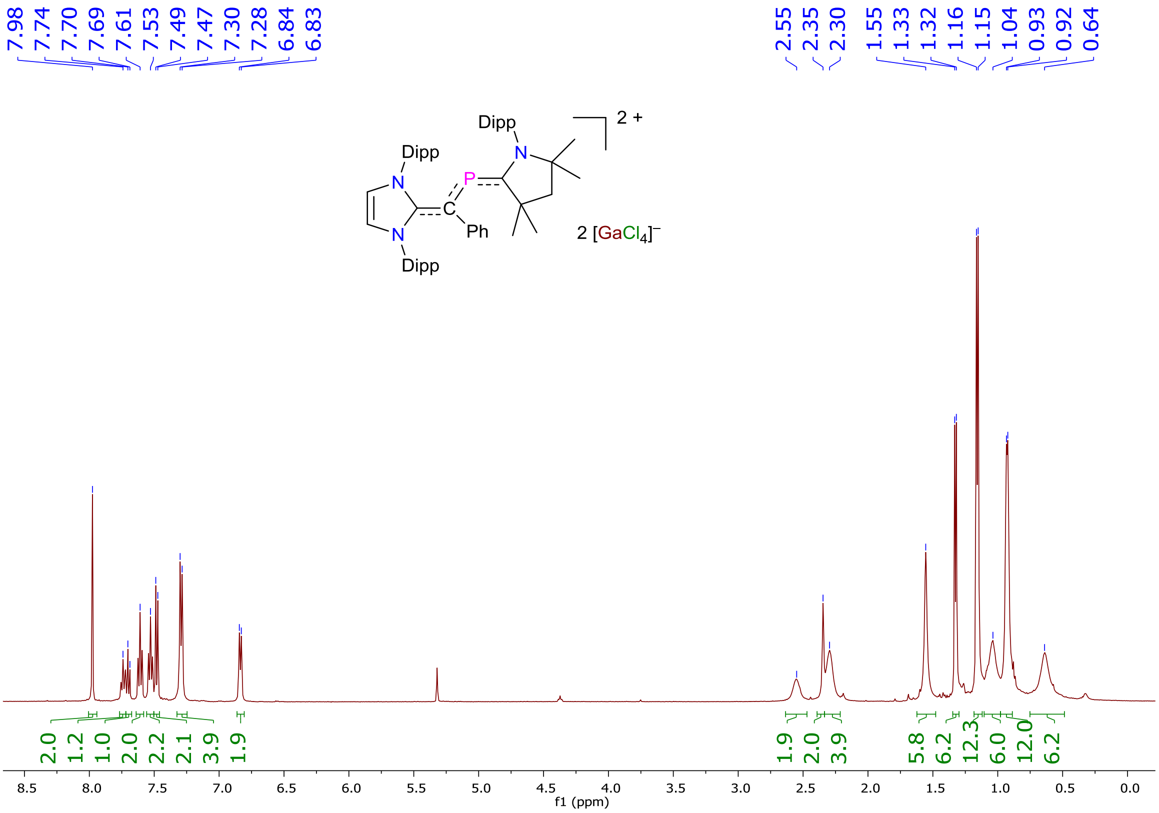

Figure S13. ^{1}H NMR (500 MHz, CD_2Cl_2, 298 K) spectrum of compound **5a**.

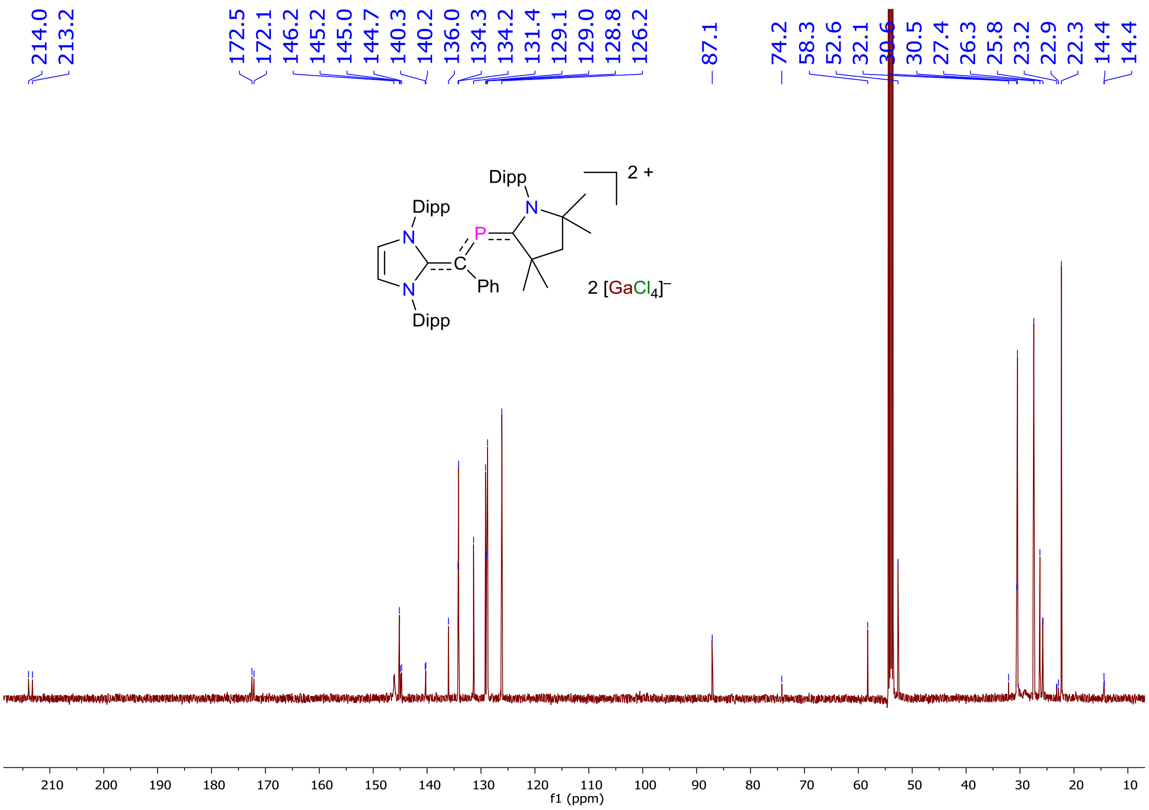

Figure S14. $^{13}C\{^{1}H\}$ NMR (125 MHz, CD_2Cl_2, 298 K) spectrum of compound **5a**.

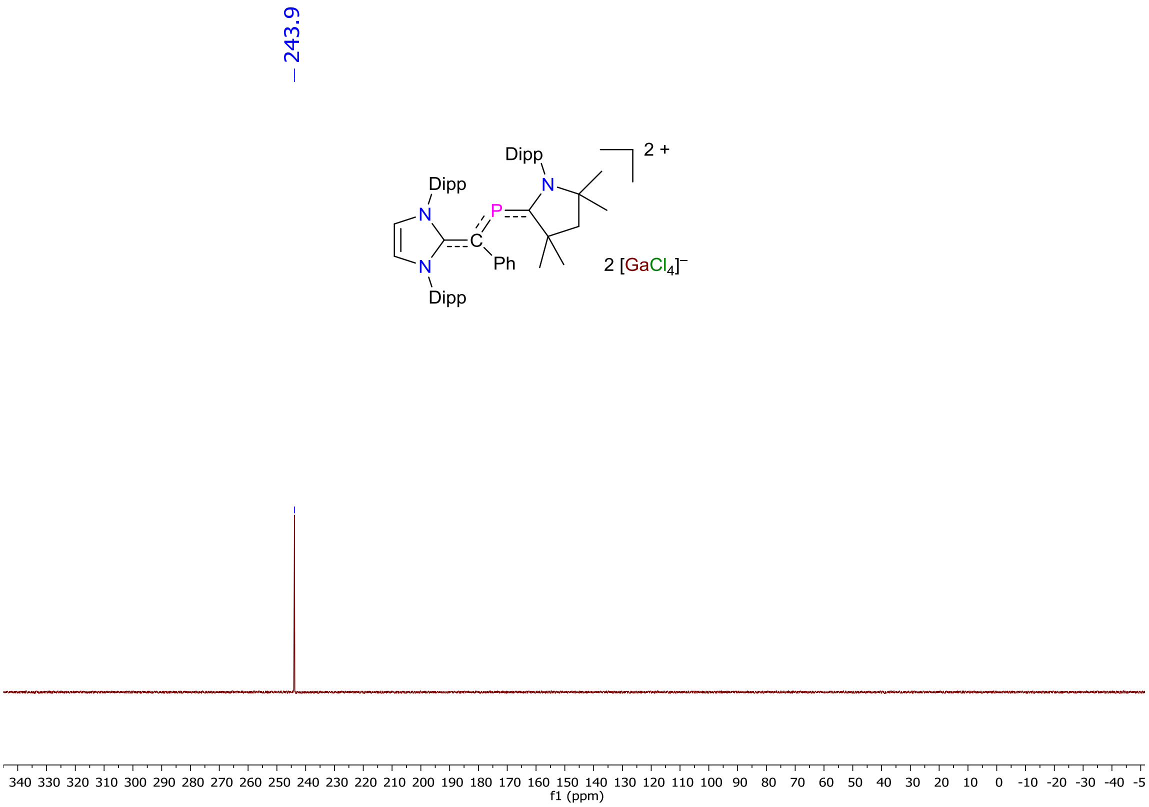

Figure S15. $^{31}P\{^{1}H\}$ NMR (202 MHz, CD_2Cl_2, 298 K) spectrum of compound **5a**.

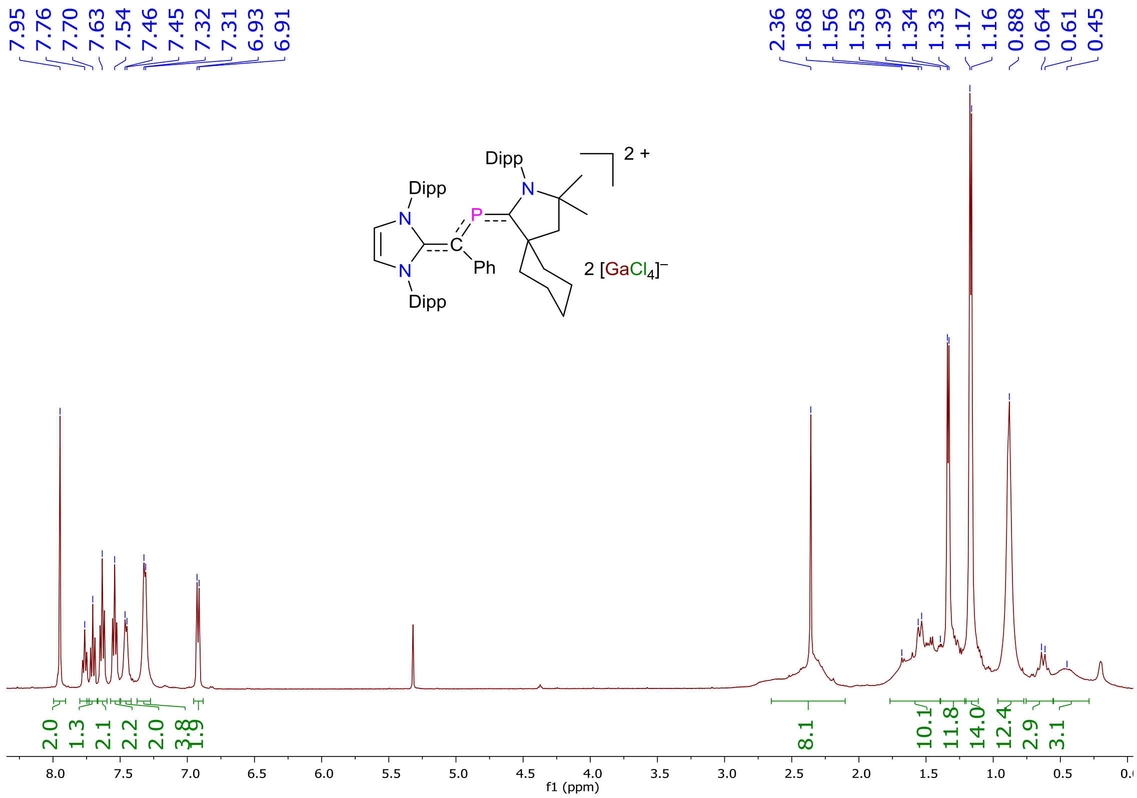

Figure S16. ^{1}H NMR (500 MHz, CD_2Cl_2, 298 K) spectrum of compound **5b**.

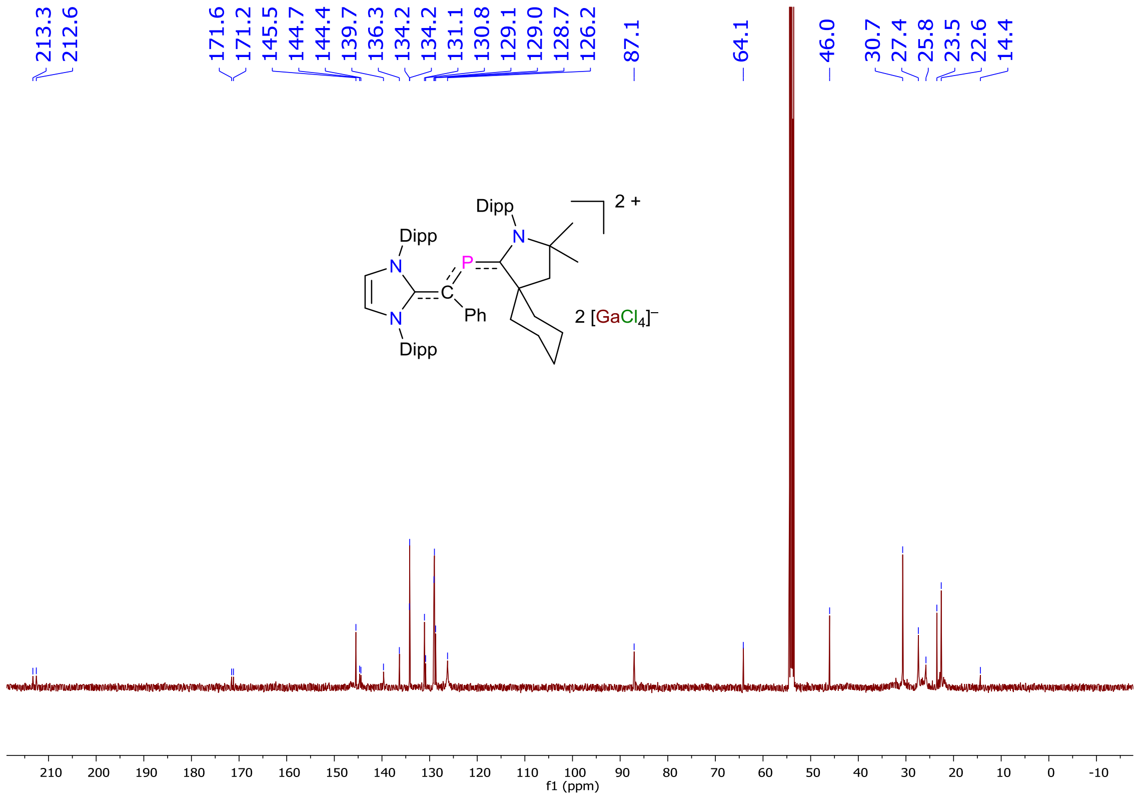

Figure S17. $^{13}C\{^{1}H\}$ NMR (125 MHz, CD_2Cl_2, 298 K) spectrum of compound **5b**.

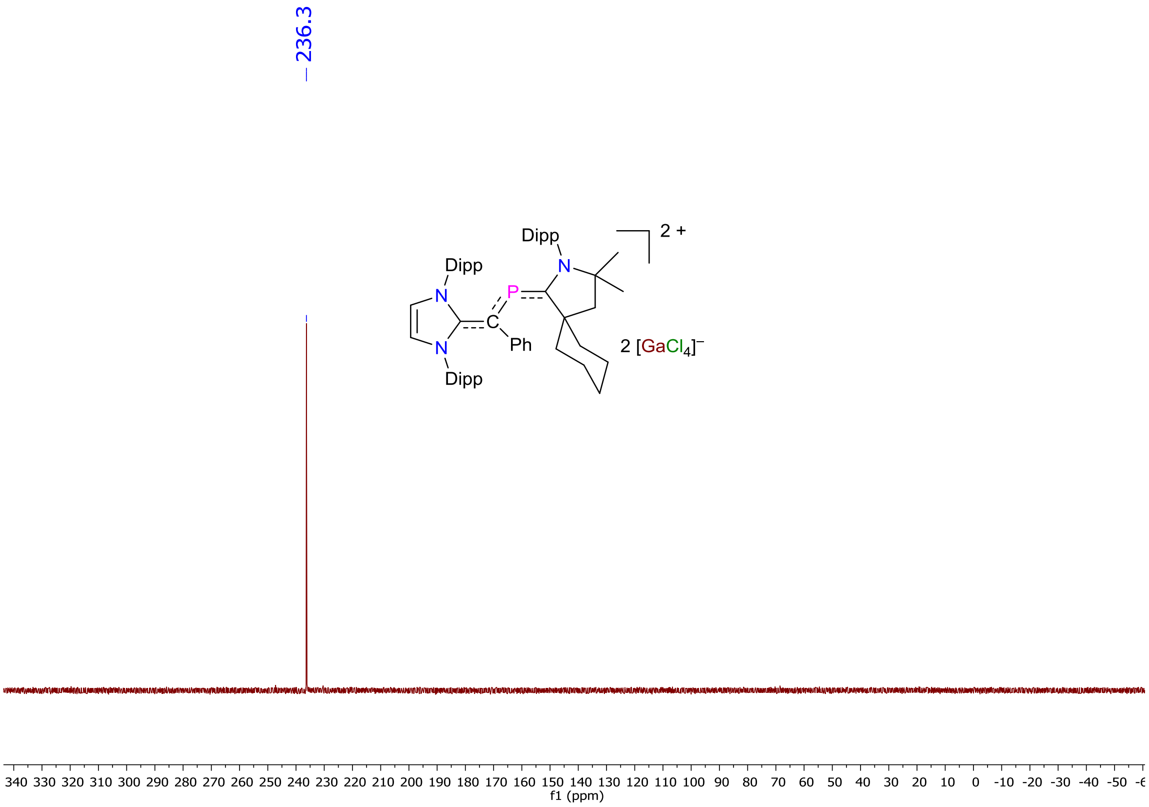

Figure S18. $^{31}P\{^{1}H\}$ NMR (202 MHz, CD_2Cl_2, 298 K) spectrum of compound **5b**.

Cyclic Voltammetry

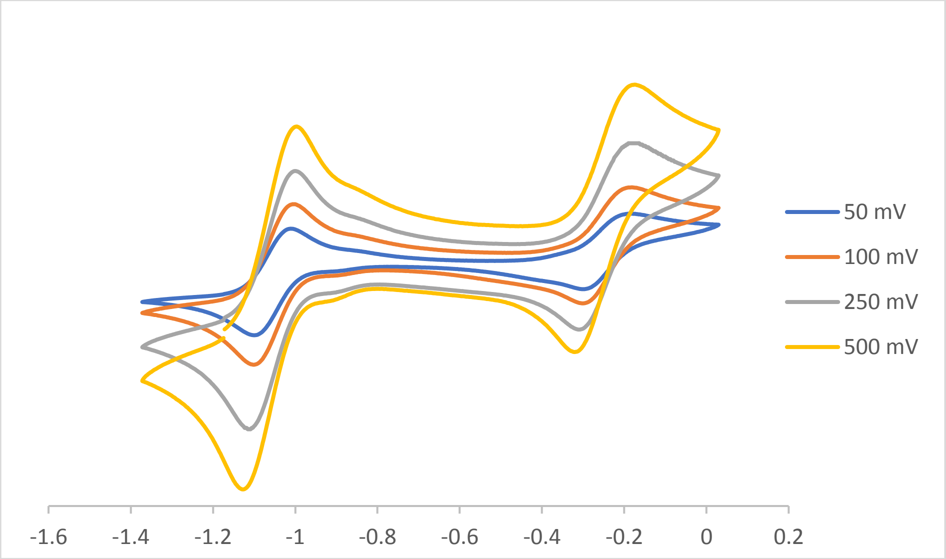

Figure S19. Cyclic voltammogram of **3a** in DCM (0.01 M *n*-$Bu_4N[Al(OC(CF_3)_3]_4$ as a supporting electrolyte, at 50, 100, 250, and 500 mVs^{-1}, *vs* Fc/Fc^+). The cycle for Fc/Fc^+ couple has been removed for clarity.

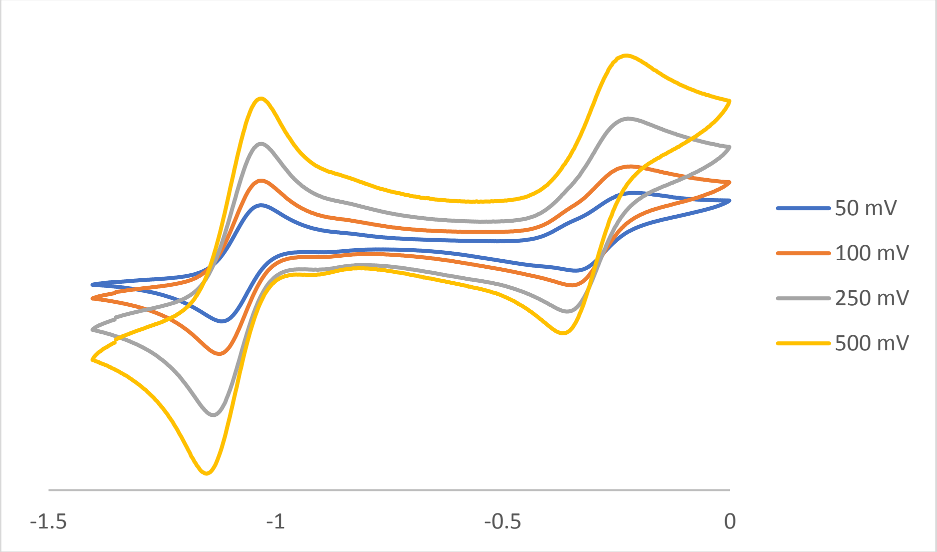

Figure S20. Cyclic voltammogram of **4a** in DCM (0.01 M *n*-$Bu_4N[Al(OC(CF_3)_3]_4$ as a supporting electrolyte, at 50, 100, 250, and 500 mVs^{-1}, *vs* Fc/Fc^+). The cycle for Fc/Fc^+ couple has been removed for clarity.

UV-vis Spectra

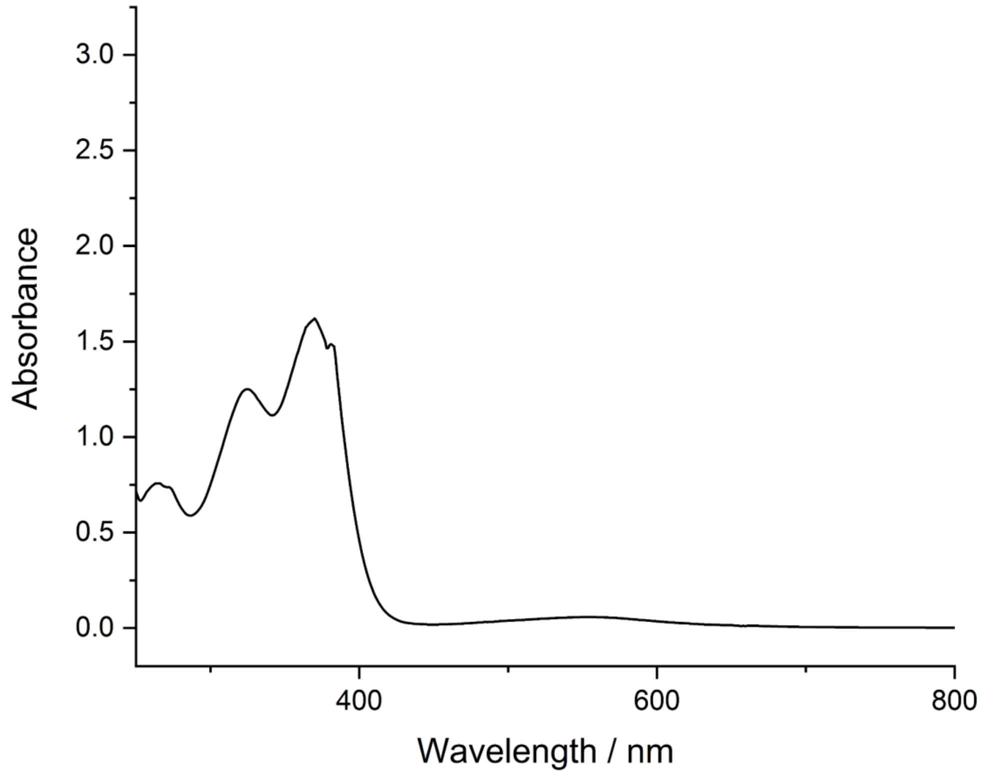

Figure S21. UV-vis spectrum of **2a** (10^{-4} M) recorded in THF.

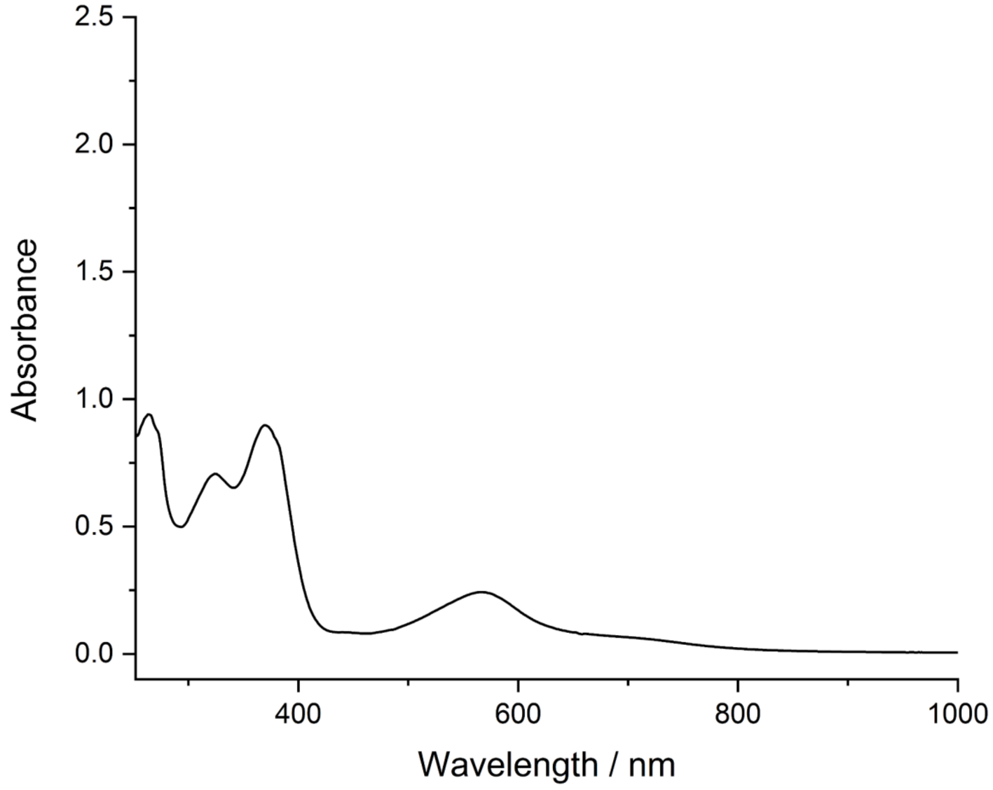

Figure S22. UV-vis spectrum of **2b** (10^{-4} M) recorded in THF.

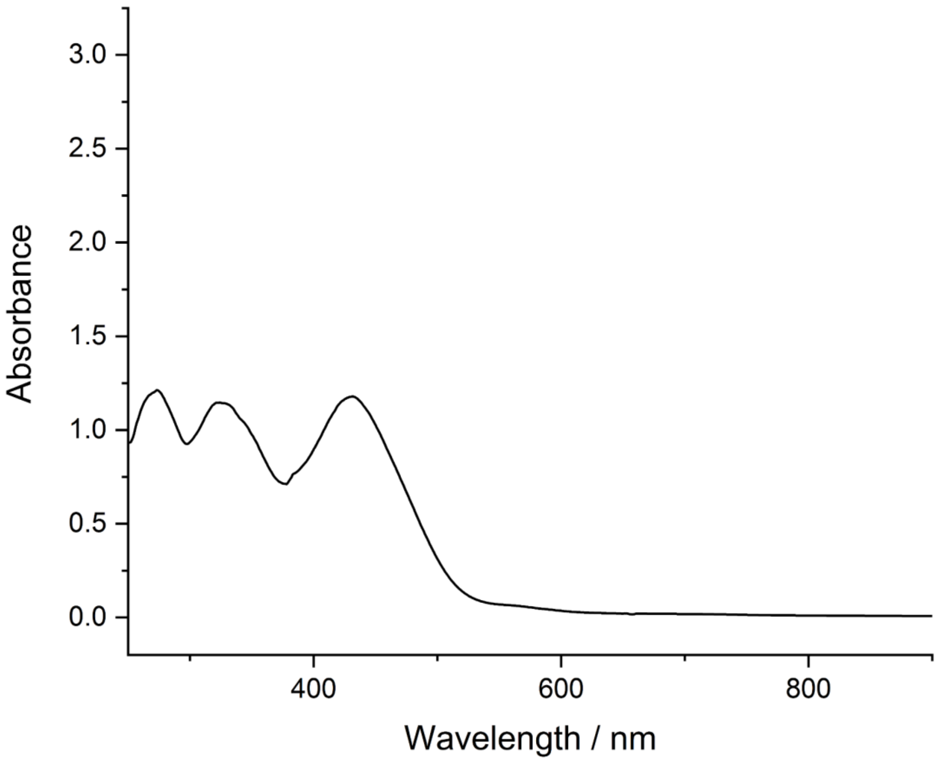

Figure S23. UV-vis spectrum of **3a** (10^{-4} M) recorded in THF.

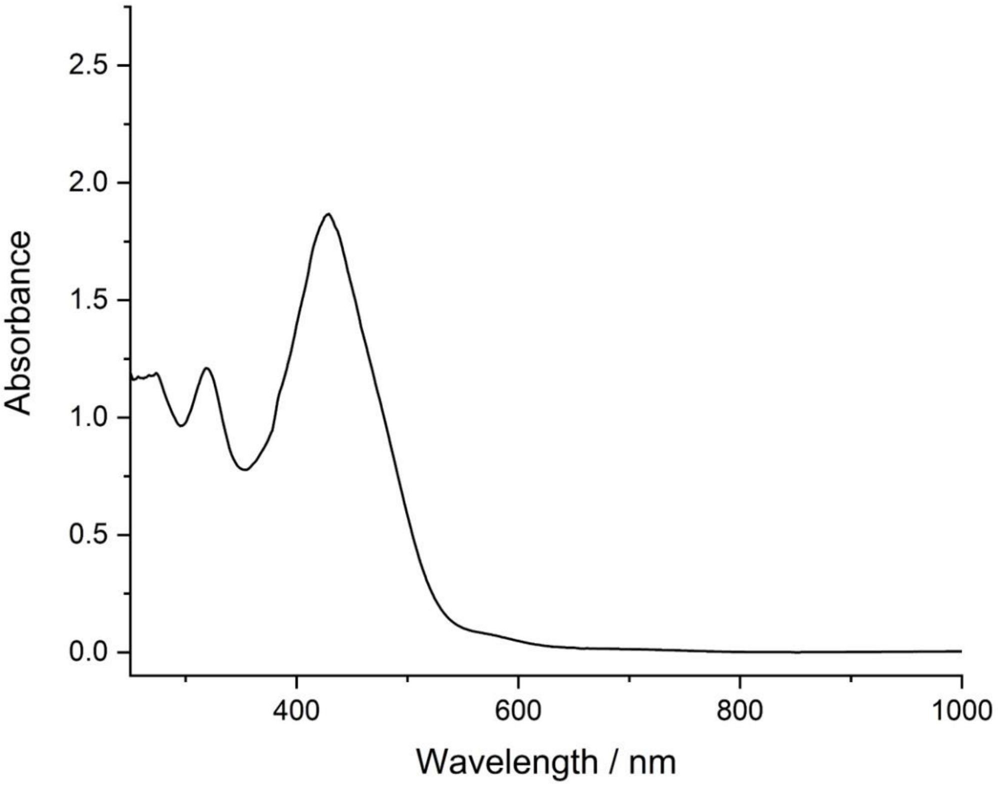

Figure S24. UV-vis spectrum of **3b** (10^{-4} M) recorded in THF.

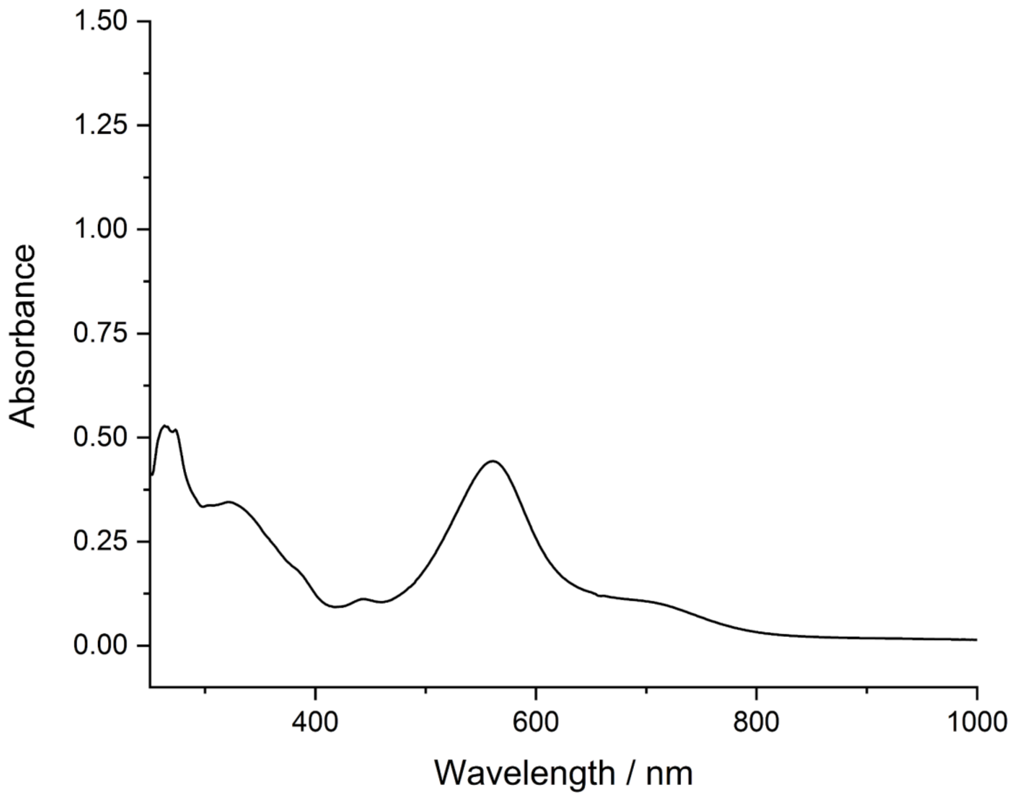

Figure S25. UV-vis spectrum of **4a** (10^{-4} M) recorded in THF.

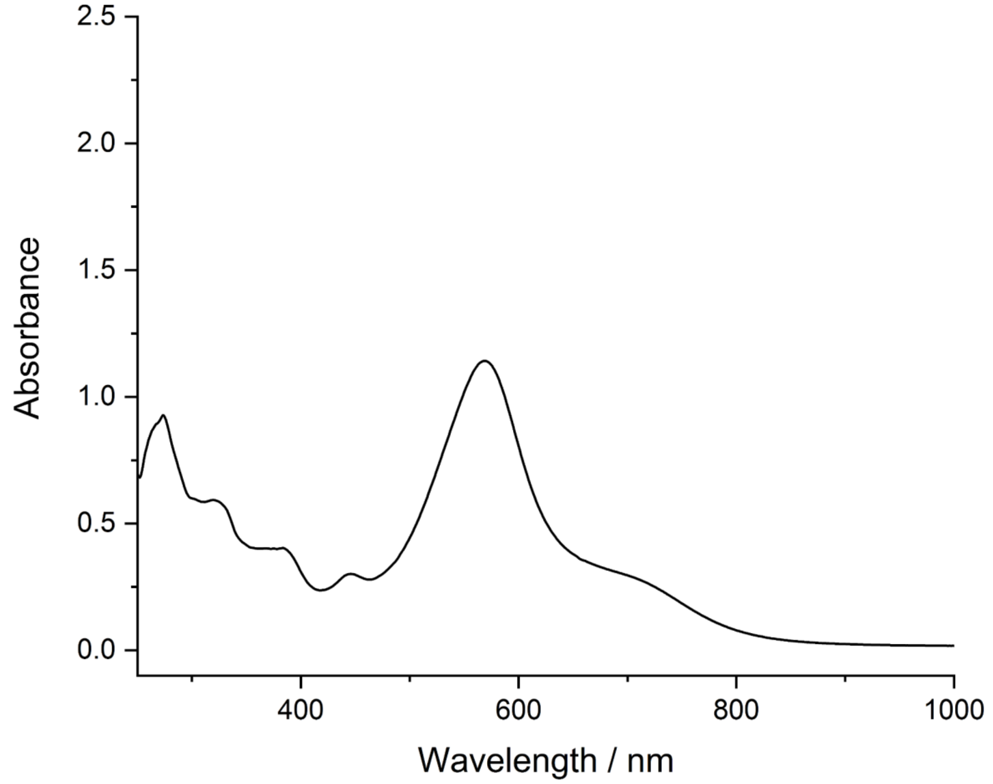

Figure S26. UV-vis spectrum of **4b** (10^{-4} M) recorded in THF.

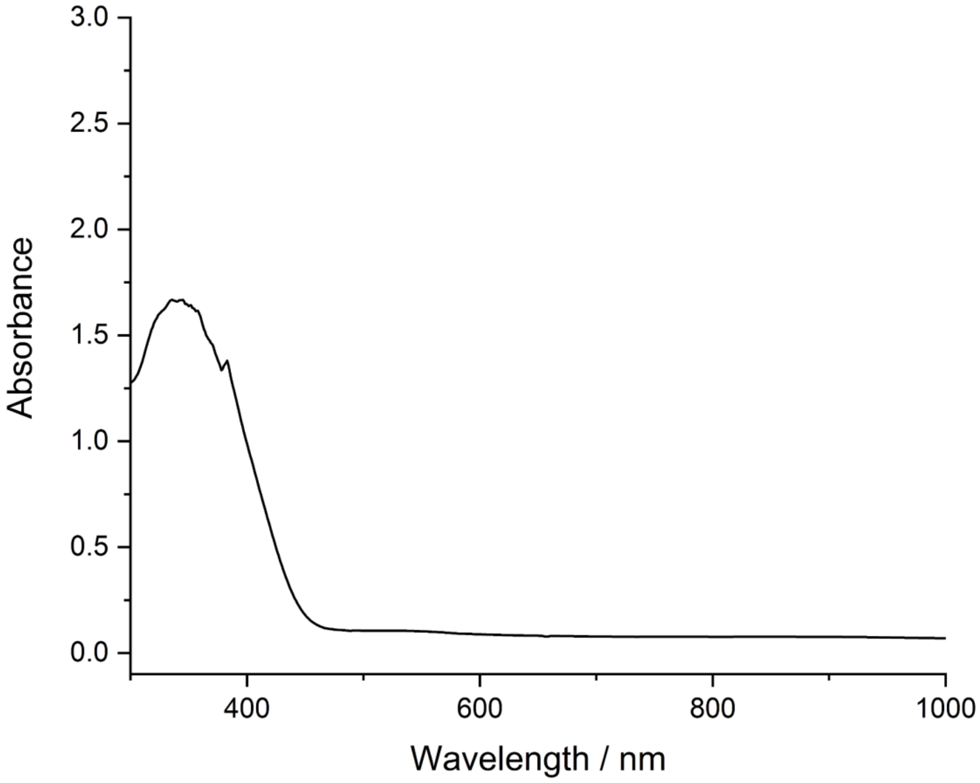

Figure S27. UV-vis spectrum of **5a** (10^{-4} M) recorded in THF.

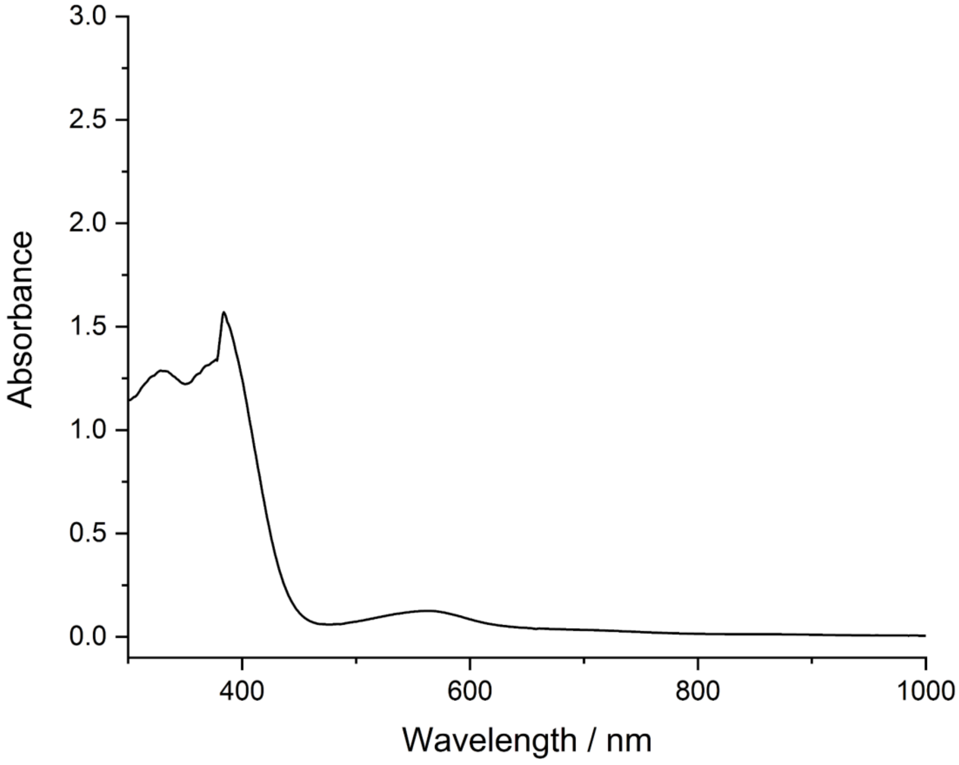

Figure S28. UV-vis spectrum of **5b** (10^{-4} M) recorded in THF.

EPR Spectra

The continuous wave (CW) EPR experiments were performed at room temperature (298 K) as well as at 80 K on a Bruker standard ST9402 resonator and with a Bruker ELEXSY E500 spectrometer. The microwave frequency was 9.628 GHz and the modulation amplitude was 0.3 mT.

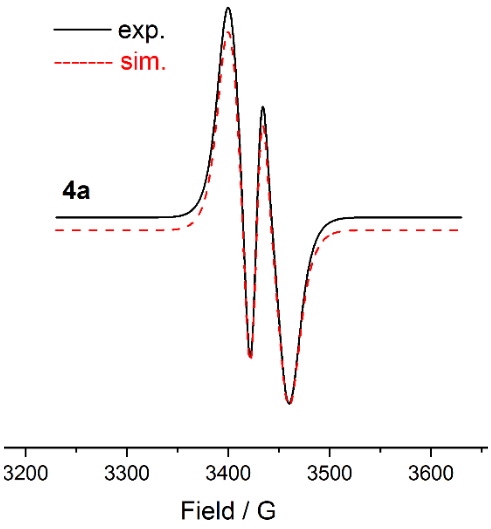

Figure S29. EPR spectrum of compound **4a** (1 mM THF solution) at 80 K. Microwave freq. 9.628 GHz, power = 2 mW, mod. freq. 100 KHz.

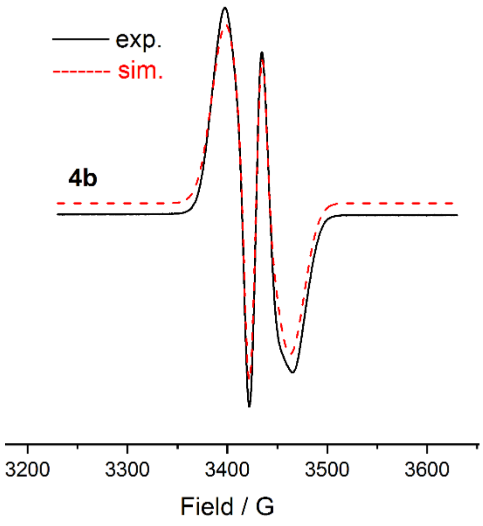

Figure S30. EPR spectrum of compound **4b** (1 mM THF solution) at 80 K. Microwave freq. 9.628 GHz, power = 2 mW, mod. freq. 100 KHz.

Crystallographic Details

The single crystal data were examined on a Rigaku Supernova diffractometer using either MoKα (λ = 0.71073 Å) or CuKα (λ = 1.54184 Å) radiation. The crystals were kept at 100.0(1) K during data collection. Using Olex2,[4] the structure was solved with the ShelXT[5] structure solution program using Intrinsic Phasing and refined with the ShelXL[6] refinement package using Least Squares minimization. Hydrogen atoms were taken into account using a riding model. Details of the X-ray investigation are given in Table S1, S2, and S3. CCDC 1949887-1949892, 1951632 contains the supplementary crystallographic data for this paper. These data can be obtained free of charge from The Cambridge Crystallographic Data Centre via www.ccdc.cam.ac.uk/conts/retrieving.html.

Table S1. Crystal data and structure refinement for compounds **2a**, **3a**, and **3b**.

	**2a**	**3a**	**3b**
Empirical formula	$C_{55}H_{72}ClF_3N_3O_3PS$	$C_{54}H_{72}N_3P$	$C_{57}H_{76}N_3P$
Formula weight	978.63	794.11	834.17
Temperature/K	100.0(1)	100.0(1)	100.0(1)
Crystal system	triclinic	monoclinic	monoclinic
Space group	P-1	$P2_1/n$	$P2_1/c$
a/Å	11.0592(4)	12.56270(10)	18.56527(11)
b/Å	12.9006(3)	15.9337(2)	11.96597(7)
c/Å	18.7834(5)	23.6169(2)	22.85445(16)
α/°	98.063(2)	90	90
β/°	91.131(2)	93.5540(10)	101.6144(6)
γ/°	103.315(3)	90	90
Volume/Å^3	2578.25(13)	4718.31(8)	4973.19(5)
Z	2	4	4
ρ_{calc}g/cm^3	1.261	1.118	1.114
μ/mm^{-1}	1.785	0.788	0.770
F(000)	1044.0	1728.0	1816.0
Crystal size/mm^3	0.5 × 0.31 × 0.03	0.45 × 0.31 × 0.22	0.43 × 0.33 × 0.25
Radiation	Cu Kα	Cu Kα	Cu Kα
2θ range for data collection/°	4.758 to 153.386	6.696 to 153.434	4.86 to 153.822
Index ranges	-13 ≤ h ≤ 11, -16 ≤ k ≤ 16, -23 ≤ l ≤ 23	-15 ≤ h ≤ 15, -19 ≤ k ≤ 19, -29 ≤ l ≤ 29	-23 ≤ h ≤ 23, -15 ≤ k ≤ 15, -28 ≤ l ≤ 28
Reflections collected	46168	86959	142708
Independent reflections	10686	9876	10414
Reflections with *I > 2σ(I)*	9327	9416	9389
R_{int}	0.0440	0.0355	0.0828
Data/restraints/parameters	10686/36/675	9876/0/539	10414/0/564
Goodness-of-fit on F^2	1.082	1.030	1.037
Final R indexes [*I > 2σ(I)*]	R_1 = 0.0420, wR_2 = 0.1129	R_1 = 0.0358, wR_2 = 0.0921	R_1 = 0.0419, wR_2 = 0.1173
Final R indexes [all data]	R_1 = 0.0483, wR_2 = 0.1182	R_1 = 0.0373, wR_2 = 0.0933	R_1 = 0.0459, wR_2 = 0.1215
Largest diff. peak/hole / e Å^{-3}	0.45/-0.47	0.27/-0.28	0.67/-0.45
CCDC	1949887	1949888	1949889

Table S2. Crystal data and structure refinement for compounds **4a** and **4b**.

	**4a**	**4b**
Empirical formula	$C_{54}H_{72}Cl_4GaN_3P$	$C_{115}H_{154}Cl_{10}Ga_2N_6P_2$
Formula weight	1005.63	2176.31
Temperature/K	100.0(1)	100.0(1)
Crystal system	monoclinic	monoclinic
Space group	$P2_1/c$	C2/c
a/Å	11.5123(2)	40.7612(6)
b/Å	12.8811(2)	12.3237(2)
c/Å	37.2397(5)	23.4798(3)
β/°	97.7240(10)	101.3980(10)
Volume/Å^3	5472.21(15)	11562.0(3)
Z	4	4
ρ_{calc}g/cm^3	1.221	1.250
μ/mm^{-1}	0.764	0.773
F(000)	2124.0	4592.0
Crystal size/mm^3	0.41 × 0.25 × 0.23	0.24 × 0.13 × 0.03
Radiation	MoKα	MoKα
2θ range for data collection/°	5.454 to 65.576	3.458 to 64.282
Index ranges	$-17 \leq h \leq 17, -19 \leq k \leq 19, -55 \leq l \leq 56$	$-59 \leq h \leq 60, -18 \leq k \leq 18, -33 \leq l \leq 34$
Reflections collected	120621	114564
Independent reflections	19071	19086
Reflections with $I > 2\sigma(I)$	16638	13305
R_{int}	0.0381	0.0542
Data/restraints/parameters	19071/0/584	19086/0/770
Goodness-of-fit on F^2	1.174	1.036
Final R indexes [$I > 2\sigma(I)$]	R_1 = 0.0544, wR_2 = 0.1072	R_1 = 0.0517, wR_2 = 0.1046
Final R indexes [all data]	R_1 = 0.0642, wR_2 = 0.1106	R_1 = 0.0873, wR_2 = 0.1194
Largest diff. peak/hole / e Å^{-3}	0.69/-0.64	0.90/-0.57
CCDC	1949890	1949891

Table S3. Crystal data and structure refinement for compound **5a** and **5b**.

	**5a**	**5b**
Empirical formula	$C_{54}H_{72}Cl_8Ga_2N_3P$	$C_{59}H_{80}Cl_{15}Ga_3N_3P$
Formula weight	1217.15	1603.14
Temperature/K	100.0(1)	100.0(1)
Crystal system	orthorhombic	monoclinic
Space group	Pbca	$P2_1/c$
a/Å	24.13916(17)	19.16275(15)
b/Å	18.90210(13)	13.46701(10)
c/Å	26.08922(18)	28.2924(2)
β/°	90	94.5840(7)
Volume/Å^3	11904.01(15)	7277.92(10)
Z	8	4
ρ_{calc}g/cm^3	1.358	1.463
μ/mm^{-1}	4.946	6.866
F(000)	5040.0	3272.0
Crystal size/mm^3	0.38 × 0.19 × 0.17	0.42 × 0.29 × 0.21
Radiation	Cu Kα	Cu Kα
2θ range for data collection/°	6.776 to 153.742	6.27 to 153.51
Index ranges	$-29 \leq h \leq 30$, $-23 \leq k \leq 23$, $-32 \leq l \leq 32$	$-24 \leq h \leq 24$, $-16 \leq k \leq 16$, $-35 \leq l \leq 35$
Reflections collected	220917	160048
Independent reflections	12495	15269
Reflections with $I > 2\sigma(I)$	11792	14273
R_{int}	0.0607	0.0721
Data/restraints/parameters	12495/95/669	15269/0/745
Goodness-of-fit on F^2	1.030	1.034
Final R indexes [$I > 2\sigma(I)$]	$R_1 = 0.0476$, $wR_2 = 0.1232$	$R_1 = 0.0360$, $wR_2 = 0.0954$
Final R indexes [all data]	$R_1 = 0.0497$, $wR_2 = 0.1251$	$R_1 = 0.0384$, $wR_2 = 0.0974$
Largest diff. peak/hole / e Å^{-3}	2.50/-0.87	0.68/-0.77
CCDC	1949892	1951632

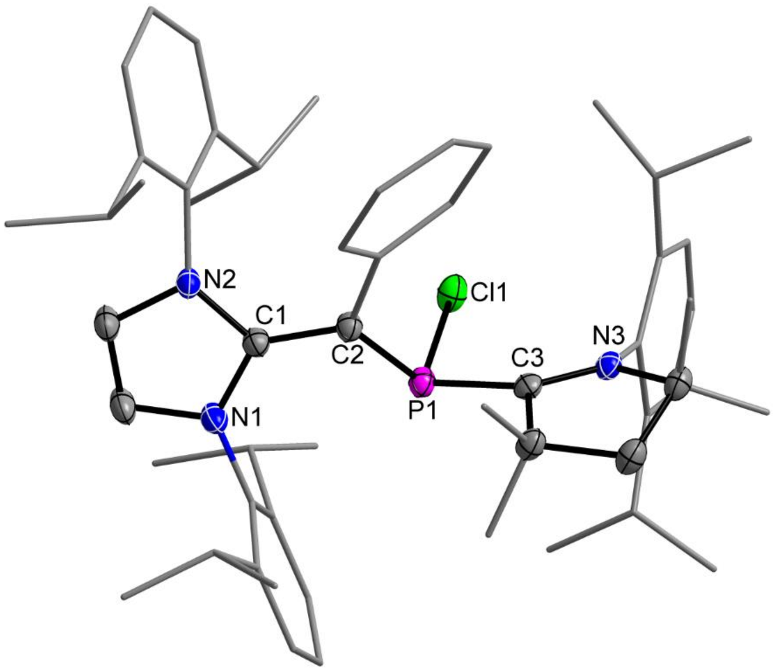

Figure S31. Molecular structure of **2a**. Hydrogen atoms and the counter anion (OTf) have been omitted for clarity. Selected bond lengths (Å) and bond angles (º): C2–P1 1.751(2), C3–P1 1.860(2), C1–C2 1.437(2), P1–Cl1 2.147(1), N1–C1 1.374(2), N2–C1 1.376(2), N3–C3 1.314(2); C2–P1–C3 104.1(1), C2–P1–Cl1 107.3(1), C3–P1–Cl1 103.0(1), C1–C2–P1 117.0(1), C2–C1–N1 128.7(1), C2–C1–N2 126.7(1), P1–C3–N3 130.0(1).

Computational Details

All geometries were optimized with the Gaussian 16 program suite[7] using the DFT functional M06-2X[8] in combination with the Ahlrichs def2-SVP[9] basis function as implemented. The stationary points were located with the Berny algorithm using redundant internal coordinates. Analytical Hessians were computed to determine the nature of stationary points (zero imaginary frequencies for minima).[10] The electronic energies were improved by single point calculations at the M06-2X/def2-TZVPP level of theory.

The Wiberg Bond Indices (WBI)[11] and NPA[12] atomic partial charges were calculated at the M06-2X/def2-TZVPP//M06-2X/def2-SVP level of theory using the NBO 3.1 interface of Gaussian.[13]

Time-dependent density functional theory (Full-TDDFT) was employed to calculate excitation energies as implemented in ORCA 4.0.1.[14] We used the functional M06-2X in combination the def2-SVP basis sets. The solvent (THF) was described by the conductor-like polarizable continuum model, CPCM.[15]

Table S4. Electronic energies of selected molecular orbitals of compounds **3a**, **3b**, **4a**, **4b**, **5a**, and **5b** calculated at M06-2X/def2-TZVPP//M06-2X/def2-SVP level of theory. For the radical cations **4a** and **4b**, the value for both (α/β) spin orbitals are given.

**orbital**	**energy / eV**					
	**3a**	**3b**	**4a**	**4b**	**5a**	**5b**
L+1	+0.24	+0.24	−2.52/−2.48	−2.49/−2.47	−6.21	−6.11
L	+0.14	+0.10	−3.01/−4.59	−3.05/−4.55	−7.74	−7.71
S/ H	−4.95	−4.95	−8.08/−9.66	−8.04/−9.63	−12.79	−12.75
S−1/ H−1	−6.52	−6.49	−10.09/−10.05	−10.05/−10.01	−12.86	−12.83
S−2/ H−2	−7.07	−7.05	−10.11/−10.24	−10.08/−10.21	−12.94	−12.91
S−3/ H−3	−7.65	−7.67	−10.29/−10.32	−10.27/−10.32	−13.00	−12.94
S−4// H−4	−7.70	−7.69	−10.33/−10.43	−10.33/−10.42	−13.19	−13.13
SOMO-LUMO or HOMO-LUMO gap	5.09	5.05	5.07	4.99	5.05	5.04
L = lowest unoccupied molecular orbital (LUMO); S = singly occupied molecular orbital (SOMO); H = highest occupied molecular orbital (HOMO)						

Table S5. Wiberg bond indices as well as natural population analysis (NPA) atomic charges of compounds **3a**, **3b**, **4a**, **4b**, **5a**, and **5b** calculated at M06-2X/def2-TZVPP//def2-SVP level of theory.

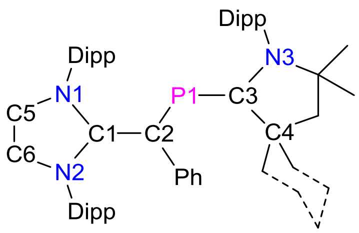

bond	Wiberg bond indices					
	**3a**	**3b**	**4a**	**4b**	**5a**	**5b**
P1–C2	0.95	0.95	1.22	1.22	1.63	1.63
C1–C2	1.49	1.49	1.20	1.20	1.06	1.06
C1–N1	1.08	1.08	1.20	1.20	1.27	1.26
C1–N2	1.07	1.07	1.19	1.19	1.26	1.26
N1–C5	1.06	1.06	1.11	1.11	1.16	1.16
N2–C6	1.07	1.07	1.12	1.12	1.17	1.17
C5–C6	1.72	1.72	1.65	1.65	1.60	1.60
P1–C3	1.57	1.56	1.19	1.18	0.92	0.91
C3–N3	1.11	1.11	1.32	1.32	1.61	1.61
C3–C4	0.96	0.96	0.97	0.98	0.98	0.99
**atom**	**NPA atomic charge**					
	**3a**	**3b**	**4a**	**4b**	**5a**	**5b**
P1	+0.49	+0.49	+0.65	+0.65	+0.85	+0.85
C2	−0.59	−0.59	−0.54	−0.55	−0.45	−0.46
C1	+0.45	+0.44	+0.47	+0.47	+0.43	+0.43
N1	−0.44	−0.44	−0.36	−0.36	−0.31	−0.31
N2	−0.44	−0.44	−0.36	−0.36	−0.31	−0.31
C5	−0.09	−0.09	−0.06	−0.06	−0.04	−0.04
C6	−0.08	−0.08	−0.05	−0.05	−0.03	−0.03
C3	−0.08	−0.08	+0.04	+0.04	+0.23	+0.24
N3	−0.50	−0.50	−0.40	−0.40	−0.33	−0.33
C4	−0.13	−0.11	−0.13	−0.11	−0.14	−0.13

Table S6. Wavelength (*λ*), oscillator strength (*f*) and main assignment of the TD-PCM(thf)/M06-2X/def2-SVP results for compound **3a**; threshold for printing excitations was chosen to be $f \geq 0.06$.

state no.	*λ* / nm	*f*	Assignment
1	409.2	0.1398	H → L (*c* = 0.9131)
2	377.0	0.1981	H → L+2 (*c* = 0.7205)
10	285.5	0.0708	H−1 → L (*c* = 0.5147)
44	197.8	0.0711	H−4 → L (*c* = 0.2549)<br>H−5 → L+6 (*c* = 0.2532)

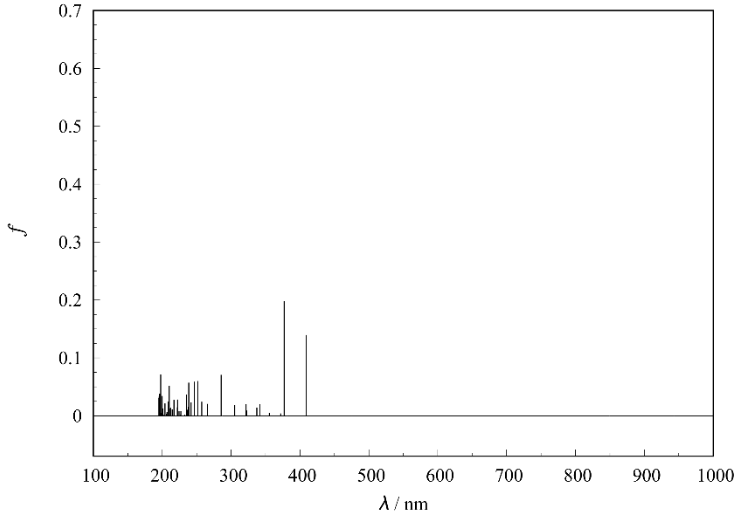

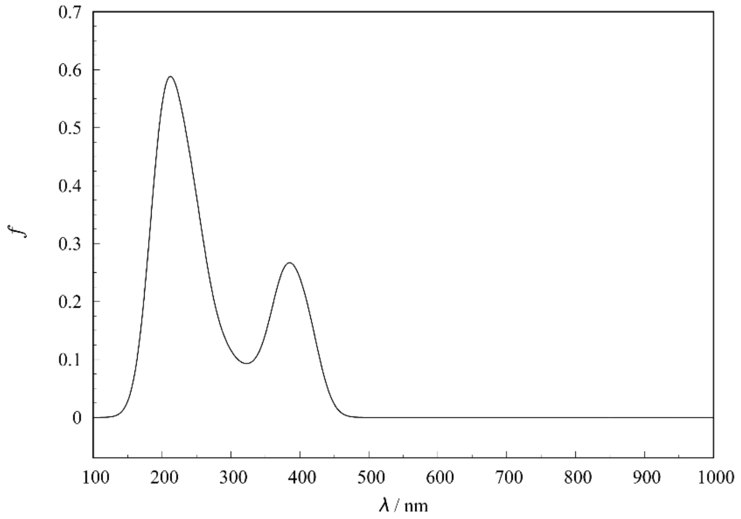

Figure S32. UV-visible spectrum (lines: top; Gaussian line broadening with a full width at half maximum (fwhm) of 50 cm^{-1}: bottom) of **3a** calculated at TD-PCM(thf)/M06-2X/def2-SVP.

Table S7. Wavelength (λ), oscillator strength (f) and main assignment of the TD-PCM(thf)/M06-2X/def2-SVP results for compound **3b**; threshold for printing excitations was chosen to be $f \geq 0.06$.

**state no.**	**λ / nm**	***f***	**Assignment**
1	414.4	0.1523	H → L (c = 0.9141)
2	379.3	0.1652	H → L+2 (c = 0.5450)
10	289.4	0.0717	H−1 → L (c = 0.5674)
13	252.4	0.0792	H−1 → L+2 (c = 0.2306)
17	239.4	0.0673	H → L+10 (c = 0.1404)
36	207.3	0.0785	H−3 → L+1 (c = 0.1828)
46	198.0	0.0640	H−2 → L+6 (c = 0.3371)

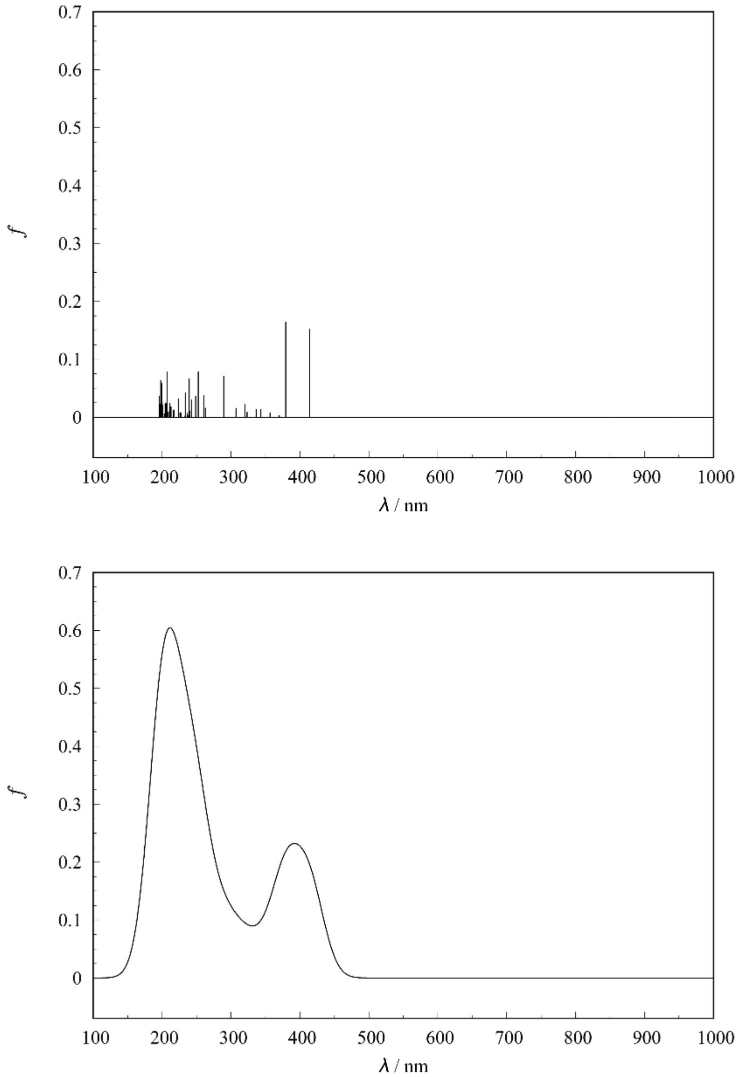

Figure S33. UV-visible spectrum (lines: top; Gaussian line broadening with a full width at half maximum (fwhm) of 50 cm^{-1}: bottom) of **3b** calculated at TD-PCM(thf)/M06-2X/def2-SVP.

Table S8. Wavelength (λ), oscillator strength (f) and main assignment of the TD-PCM(thf)/M06-2X/def2-SVP results for compound **4a**; threshold for printing excitations was chosen to be $f \geq 0.03$.

**state no.**	λ **/ nm**	f	**Assignment**
2	514.8	0.2862	S → L (c = 0.6181)
4	386.7	0.0351	S → L+1 (c = 0.7508)
24	278.0	0.0344	S−12 → S (c = 0.1543)
49	230.2	0.0431	S−1 → L+1 (c = 0.1646)

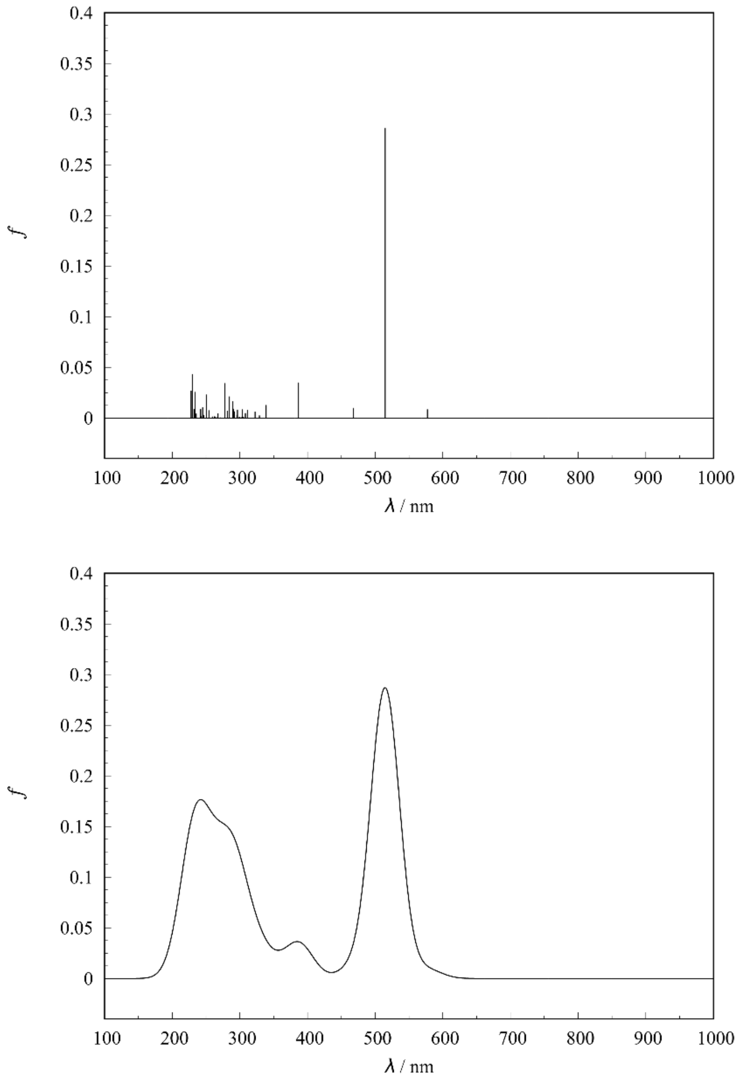

Figure S34. UV-visible spectrum (lines: top; Gaussian line broadening with a full width at half maximum (fwhm) of 50 cm^{-1}: bottom) of **4a** calculated at TD-PCM(thf)/M06-2X/def2-SVP.

Table S9. Wavelength (λ), oscillator strength (f) and main assignment of the TD-PCM(thf)/M06-2X/def2-SVP results for compound **4b**; threshold for printing excitations was chosen to be $f \geq 0.03$.

**state no.**	**λ / nm**	***f***	**Assignment**
2	518.6	0.2522	S−1 → S (c = 0.6697)
4	384.3	0.0373	S → L+1 (c = 0.7536)
23	280.5	0.0375	S−12 → S (c = 0.1797)
50	230.5	0.0325	S → L+11 (c = 0.1567)

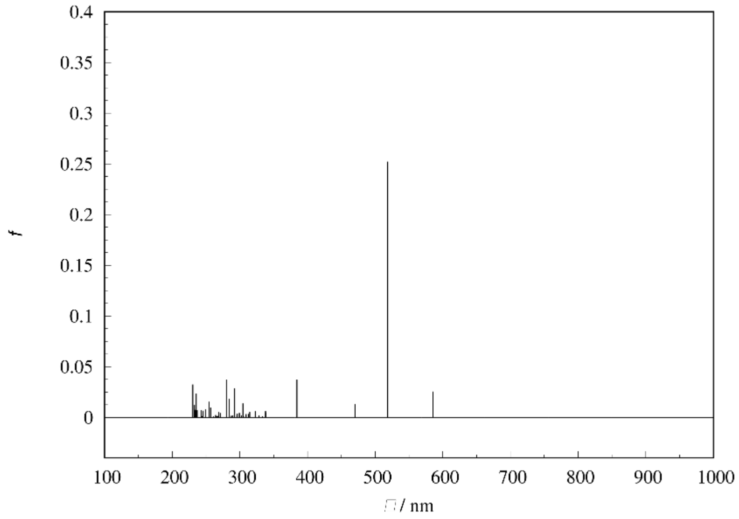

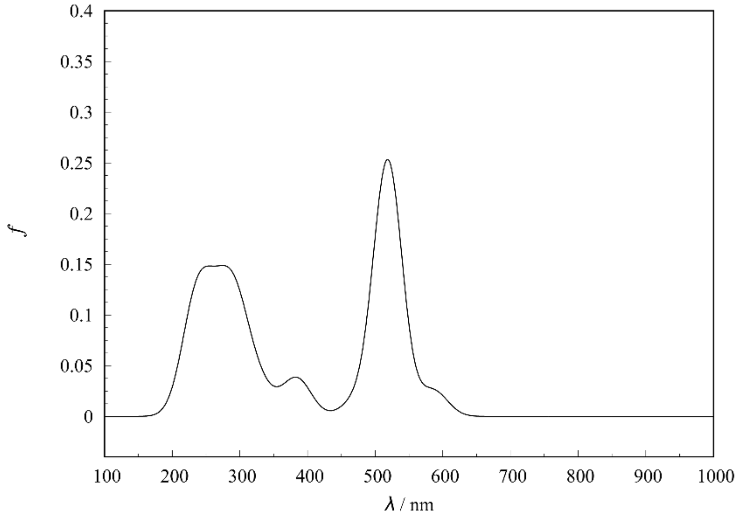

Figure S35. UV-visible spectrum (lines: top; Gaussian line broadening with a full width at half maximum (fwhm) of 50 cm^{-1}: bottom) of **4b** calculated at TD-PCM(thf)/M06-2X/def2-SVP.

Table S10. Wavelength (*λ*), oscillator strength (*f*) and main assignment of the TD-PCM(thf)/M06-2X/def2-SVP results for compound **5a**; threshold for printing excitations was chosen to be $f \geq 0.05$.

**state no.**	***λ* / nm**	***f***	**Assignment**
2	372.9	0.1203	H−4 → L (*c* = 0.3077)
6	341.0	0.0524	H−5 → L (*c* = 0.4875)
9	309.5	0.0975	H−8 → L (*c* = 0.7296)
25	225.8	0.0508	H−6 → L+1 (*c* = 0.2221)
27	220.0	0.0759	H−1 → L+2 (*c* = 0.1728)
48	199.1	0.0556	H−5 → L+2 (*c* = 0.2154)

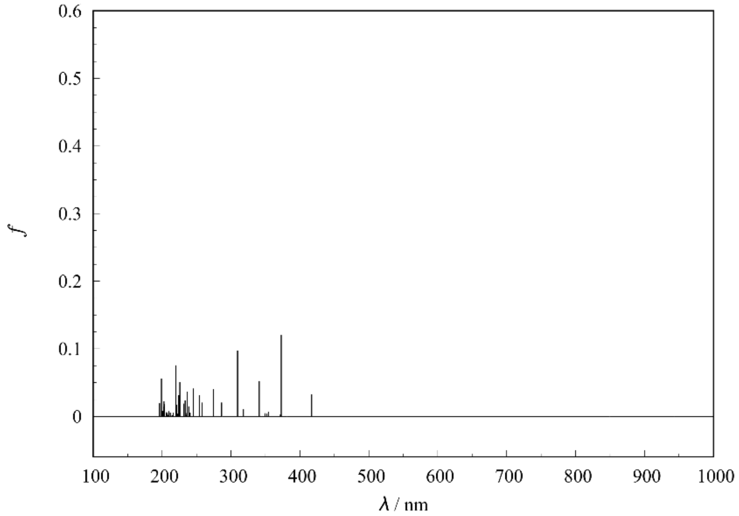

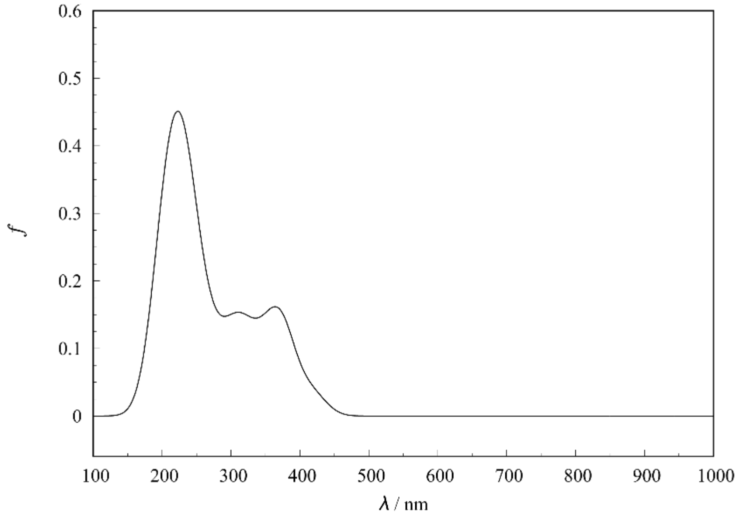

Figure S36. UV-visible spectrum (lines: top; Gaussian line broadening with a full width at half maximum (fwhm) of 50 cm^{-1}: bottom) of **5a** calculated at TD-PCM(thf)/M06-2X/def2-SVP.

Table S11. Wavelength (λ), oscillator strength (f) and main assignment of the TD-PCM(thf)/M06-2X/def2-SVP results for compound **5b**; threshold for printing excitations was chosen to be $f \geq 0.05$.

**state no.**	**λ / nm**	***f***	**Assignment**
2	377.2	0.1285	H−1 → L (c = 0.3804)
6	345.7	0.0524	H−5 → L (c = 0.6223)
9	311.0	0.1007	H−7 → L (c = 0.7891)
27	225.8	0.0596	H−6 → L+1 (c = 0.3394)
28	220.7	0.0520	H−1 → L+2 (c = 0.1362)
29	220.9	0.0506	H−6 → L+1 (c = 0.1953)

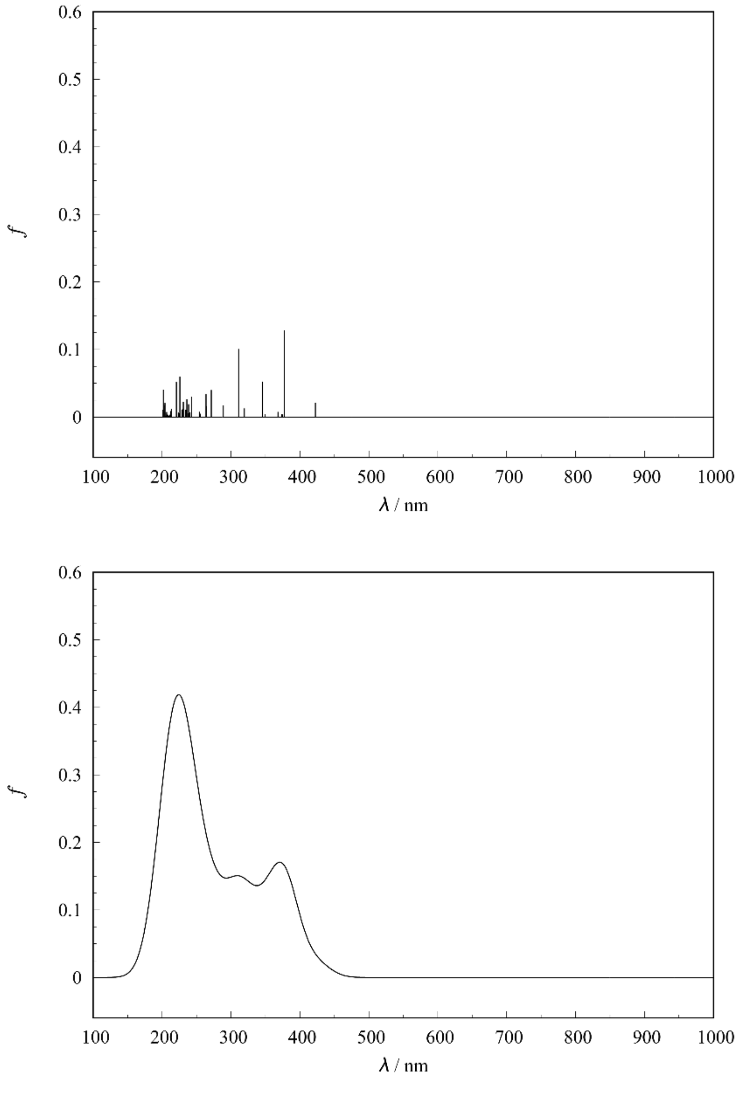

Figure S37. UV-visible spectrum (lines: top; Gaussian line broadening with a full width at half maximum (fwhm) of 50 cm^{-1}: bottom) of **5b** calculated at TD-PCM(thf)/M06-2X/def2-SVP.

Table S12. Calculated *g*-factor and hyperfine coupling constants (*A* in MHz), Löwdin and Mulliken (in parenthesis) spin densities for the phosphabutadiene radical cations **4a** and **4b** calculated at the TPSS/decon-def2-TZVP//M06-2X/def2-SVP level of theory.

	**4a**	**4b**
g-factor	2.0033601	2.0033690
A_{iso}(P)	72.7939	77.5263
$A_{iso}(N_{IPr\_cis})$	3.3114	3.2277
$A_{iso}(N_{IPr\_trans})$	4.1805	4.1326
$A_{iso}(N_{pyrollidine})$	9.0493	9.2702
$A_{iso}(H_{Ph\text{-}ortho})$	−2.1049	−1.0971
$A_{iso}(H_{Ph\text{-}ortho})$	−1.2096	−0.9908
$A_{iso}(H_{Ph\text{-}para})$	−0.6019	−0.4654
$A_{iso}(H_{carb})$	−2.2097	−2.1951
$A_{iso}(H_{carb})$	−1.4377	−1.4425
ρ_{spin}(P)	0.18(0.17)	0.19(0.18)
$\rho_{spin}(C_{vinylic})$	0.21(0.26)	0.20(0.25)
$\rho_{spin}(C_{carb\text{-}ipso})$	0.04(0.01)	0.04(0.02)
$\rho_{spin}(N_{IPr\_cis})$	0.04(0.06)	0.04(0.06)
$\rho_{spin}(N_{IPr\_trans})$	0.05(0.06)	0.05(0.06)
$\rho_{spin}(C_{IPr\_cis})$	0.03(0.04)	0.03(0.04)
$\rho_{spin}(C_{IPr\_trans})$	0.02(0.02)	0.02(0.02)
$\rho_{spin}(C_{Ph\text{-}ipso})$	0.02(−0.01)	0.02(0.00)
$\rho_{spin}(C_{Ph\text{-}ortho})$	0.01(0.01)	0.01(0.01)
$\rho_{spin}(C_{Ph\text{-}ortho})$	0.01(0.01)	0.01(0.01)
$\rho_{spin}(C_{Ph\text{-}para})$	0.01(0.01)	0.01(0.01)
$\rho_{spin}(C_{pyrolidine\text{-}ipso})$	0.15(0.19)	0.15(0.20)
$\rho_{spin}(N_{pyrolidine})$	0.14(0.17)	0.14(0.17)

Table S13. Parameters used in the Easyspin simulations of the room-temperature EPR spectra of compounds **4a** and **4b**.

	**4a**	**4b**
g-factor	2.00649	2.00647
A_{iso}(P) [MHz]	98.6	105.6
Linewidth Gaussian component [mT]	1.1928	1.1924
Linewidth Lorentzian component [mT]	0.0391	0.0391

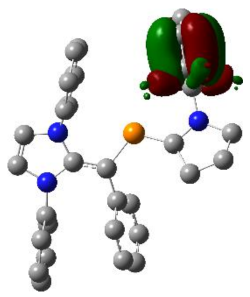

HOMO−4

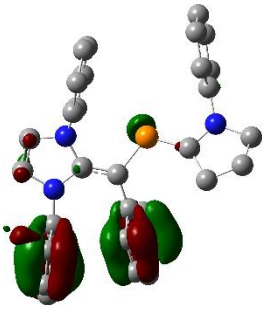

HOMO−3

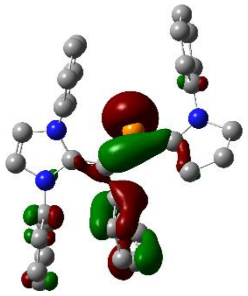

HOMO−2

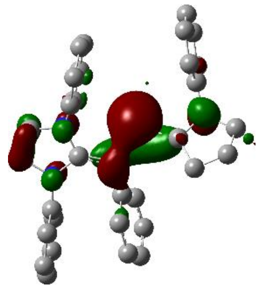

HOMO−1

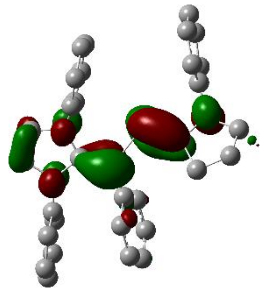

HOMO

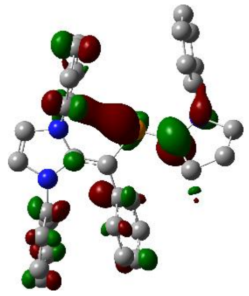

LUMO

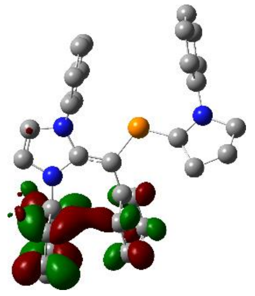

LUMO+1

Figure S38. Selected molecular orbitals (from HOMO−4 to LUMO+1) of compound **3a** calculated at M06-2X/def2-TZVPP//def2-SVP. The isovalue was arbitrarily chosen to be 0.04. Hydrogen atoms, methyl groups as well as *iso*-propyl groups were omitted for clarity reasons.

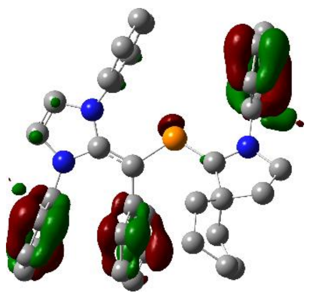

HOMO−4

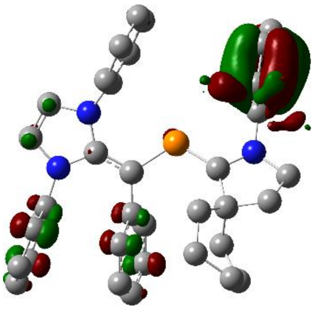

HOMO−3

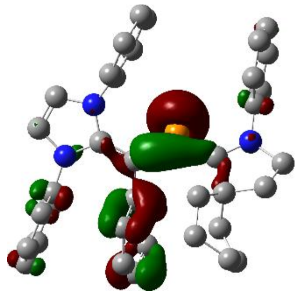

HOMO−2

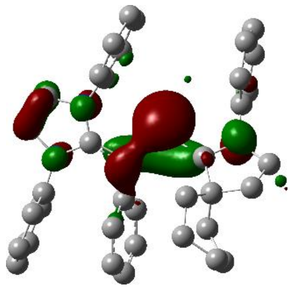

HOMO−1

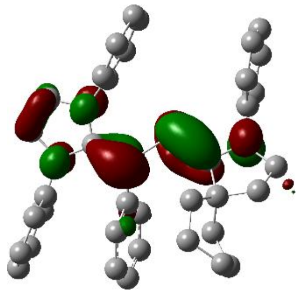

HOMO

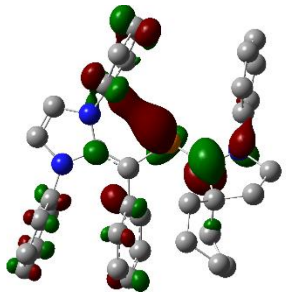

LUMO

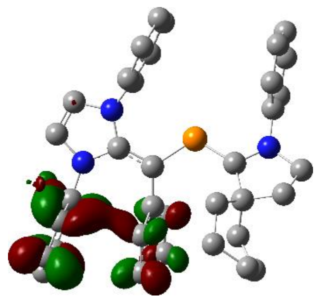

LUMO+1

Figure S39. Selected molecular orbitals (from HOMO−4 to LUMO+1) of compound **3b** calculated at M06-2X/def2-TZVPP//def2-SVP. The isovalue was arbitrarily chosen to be 0.04. Hydrogen atoms, methyl groups as well as *iso*-propyl groups were omitted for clarity reasons.

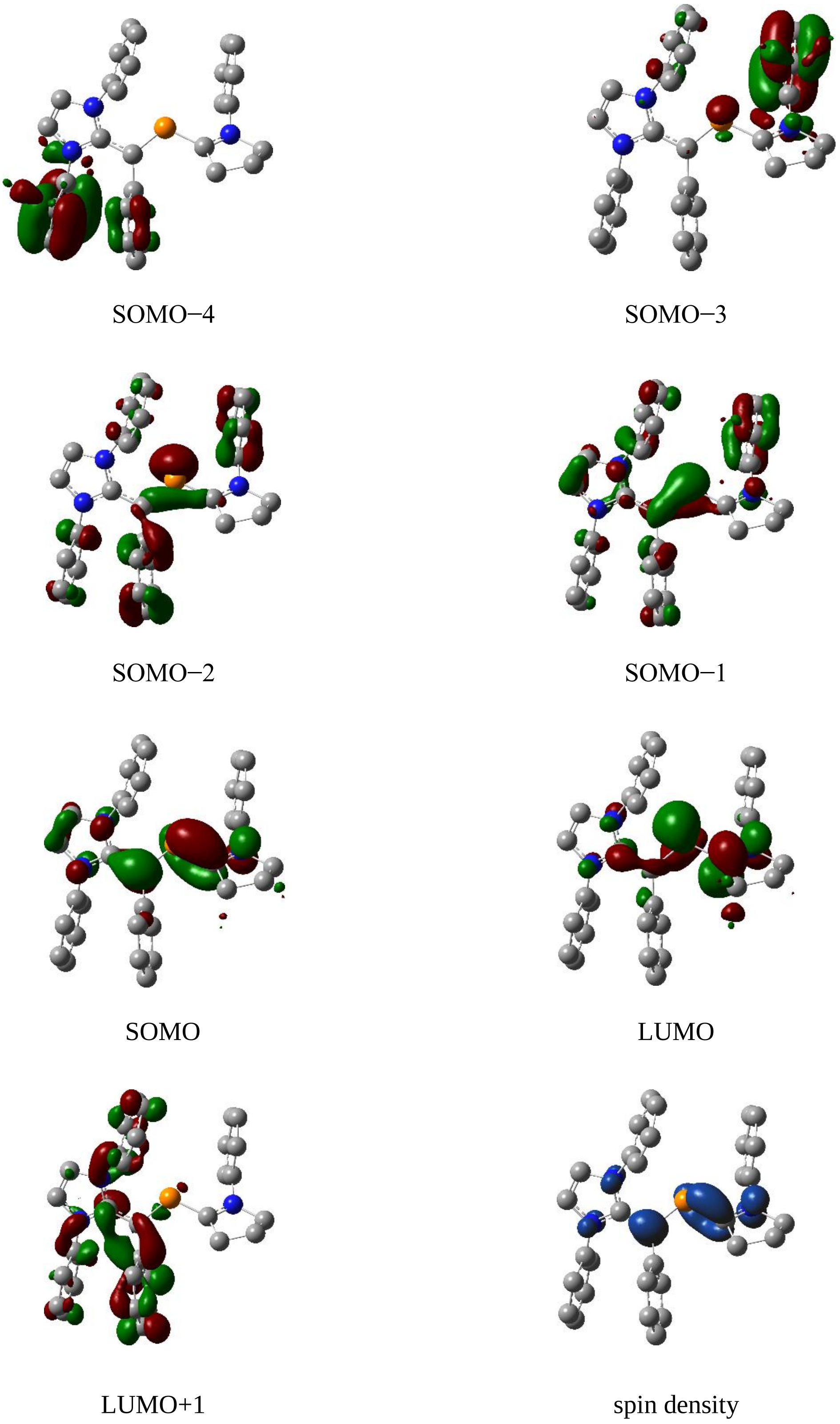

Figure S40. Selected molecular orbitals (from SOMO−4 to LUMO+1) and spin density plot of compound **4a** calculated at M06-2X/def2-TZVPP//def2-SVP. The isovalue was arbitrarily chosen to be 0.04 for molecular orbitals and 0.004 for spin density. Hydrogen atoms, methyl groups as well as *iso*-propyl groups were omitted for clarity reasons.

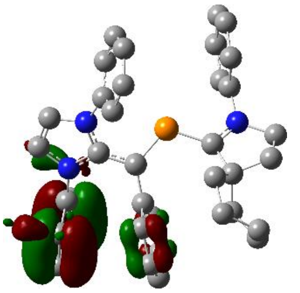

SOMO−4

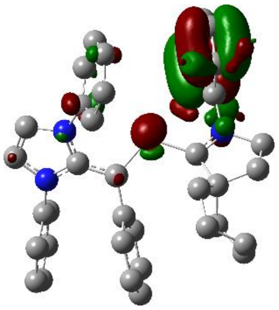

SOMO−3

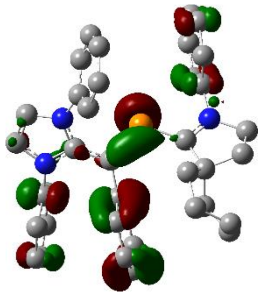

SOMO−2

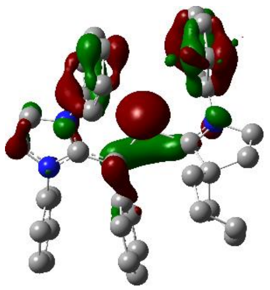

SOMO−1

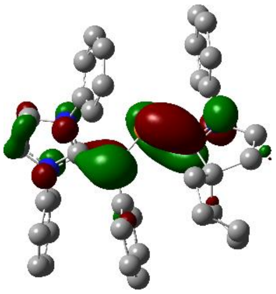

SOMO

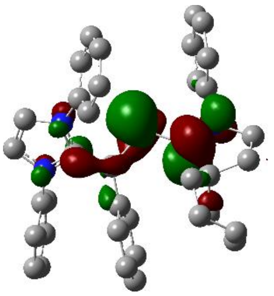

LUMO

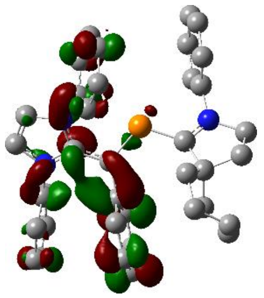

LUMO+1

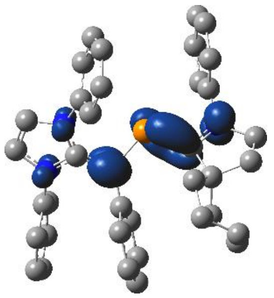

spin density

Figure S41. Selected molecular orbitals (from SOMO−4 to LUMO+1) and spin density plot of compound **4b** calculated at M06-2X/def2-TZVPP//def2-SVP. The isovalue was arbitrarily chosen to be 0.04 for molecular orbitals and 0.004 for spin density. Hydrogen atoms, methyl groups as well as *iso*-propyl groups were omitted for clarity reasons.

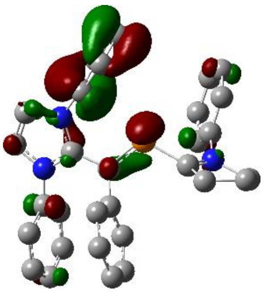

HOMO−4

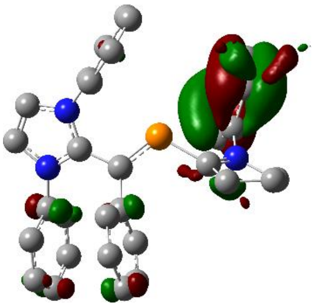

HOMO−3

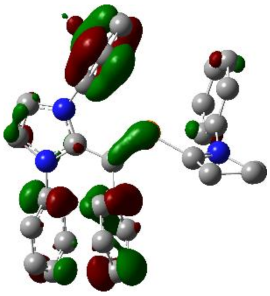

HOMO−2

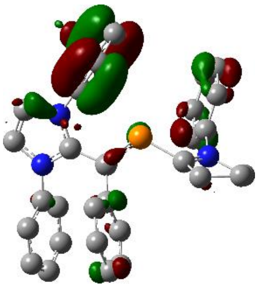

HOMO−1

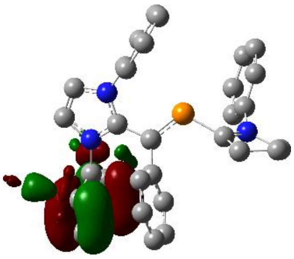

HOMO

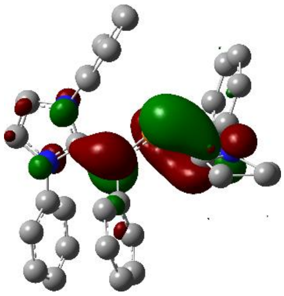

LUMO

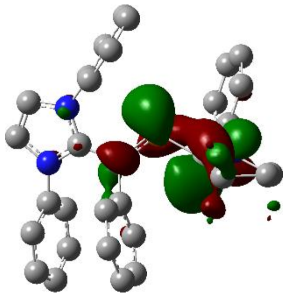

LUMO+1

Figure S42. Selected molecular orbitals (from HOMO−4 to LUMO+1) of compound **5a** calculated at M06-2X/def2-TZVPP//def2-SVP. The isovalue was arbitrarily chosen to be 0.04. Hydrogen atoms, methyl groups as well as *iso*-propyl groups were omitted for clarity reasons.

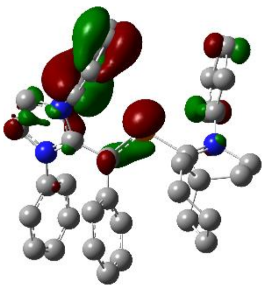

HOMO−4

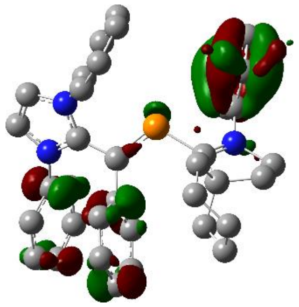

HOMO−3

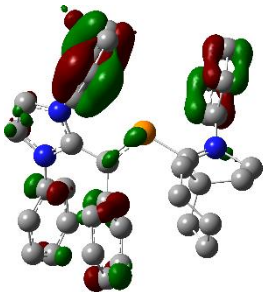

HOMO−2

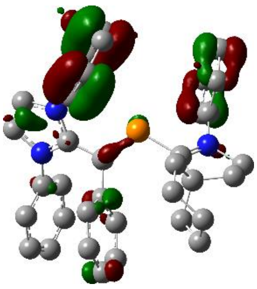

HOMO−1

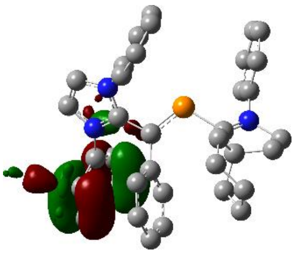

HOMO

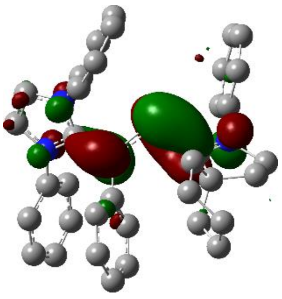

LUMO

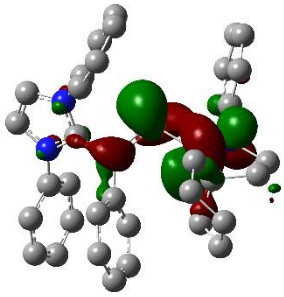

LUMO+1

Figure S43. Selected molecular orbitals (from HOMO−4 to LUMO+1) of compound **5b** calculated at M06-2X/def2-TZVPP//def2-SVP. The isovalue was arbitrarily chosen to be 0.04. Hydrogen atoms, methyl groups as well as *iso*-propyl groups were omitted for clarity reasons.

3a ν_{min} = 14 cm^{-1} *E* = −2603.9082114

C	1.724713	0.685963	0.403057
C	0.933372	-0.353093	-0.077205
C	1.566543	-1.681320	-0.309552
P	-0.866757	-0.253915	0.241243
N	1.323293	1.967184	0.810772
C	2.387046	2.635546	1.425122
C	3.460746	1.835858	1.378128
N	3.087386	0.653114	0.746024
C	0.240500	2.808795	0.383551
C	-0.792759	3.087213	1.294368
C	-1.699220	4.100170	0.965864
C	-1.569155	4.819670	-0.217138
C	0.386982	3.519247	-0.824939
C	-0.543985	4.521725	-1.110448
C	1.516036	3.198435	-1.790511
H	-0.455791	5.091568	-2.036393
H	-2.278238	5.615844	-0.451210
C	-0.898322	2.359998	2.622566
H	-2.510466	4.335803	1.657204
H	2.260312	3.644339	1.802667
H	4.471927	1.969458	1.748351
C	4.080715	-0.360000	0.553400
C	4.316625	-1.313629	1.554225
C	5.331010	-2.254267	1.314502
C	6.110082	-2.212335	0.168509
C	5.883131	-1.227537	-0.790957
C	4.859514	-0.297484	-0.624965
C	4.567254	0.737204	-1.703564
H	6.497013	-1.203759	-1.692003
H	6.897352	-2.952438	0.016564
C	3.646863	-1.389277	2.927371
H	5.512270	-3.028521	2.064212
C	-0.386479	3.238289	3.769900
H	0.664488	3.526851	3.624099
H	-0.460343	2.699293	4.726219
H	-0.983454	4.160121	3.851545
H	-0.254672	1.470987	2.558638
C	-2.324049	1.878564	2.884682
H	-3.012061	2.717493	3.075036
H	-2.349099	1.223833	3.769509
H	-2.700065	1.308786	2.024522
H	2.346215	2.779284	-1.199131
C	2.057985	4.429751	-2.516595
C	1.064154	2.127172	-2.789849
H	0.694764	1.233650	-2.267396
H	1.897638	1.830198	-3.446892
H	0.251296	2.516518	-3.423531
H	2.335873	5.225759	-1.810765
H	1.323571	4.839958	-3.225825
H	2.950548	4.157518	-3.098733
C	4.878560	0.231226	-3.114381
C	5.318473	2.051547	-1.454768
H	3.487135	0.952044	-1.658459
H	5.963122	0.159144	-3.286221
H	4.479233	0.933861	-3.860350
H	4.439956	-0.759428	-3.303602
H	6.404872	1.872979	-1.444902
H	5.036063	2.513261	-0.500123
H	5.098439	2.773846	-2.255703
C	2.302853	-0.693089	3.161635
C	4.649004	-0.909133	3.990009
H	3.478295	-2.467120	3.092508
H	4.861441	0.162484	3.850992
H	5.602446	-1.452140	3.933478
H	4.231724	-1.042396	4.998990
H	2.406806	0.400566	3.218285
H	1.906043	-1.032111	4.130198
H	1.558443	-0.926675	2.392411
C	-1.754755	-1.288355	-0.822290
N	-3.110877	-1.491245	-0.568813
C	-1.396409	-2.080105	-2.098438
C	-0.669024	-3.420660	-1.825030
C	-0.574125	-1.229817	-3.069907
C	-2.792639	-2.346776	-2.706786

C	-3.753432	-2.447289	-1.521329
C	-5.179387	-2.045726	-1.884639
C	-3.804295	-3.885435	-0.984778
H	-2.803148	-3.248738	-3.335012
H	-3.089660	-1.498729	-3.343086
H	0.414832	-3.319891	-1.940442
H	-1.009085	-4.183041	-2.541927
H	-0.849182	-3.796228	-0.810408
H	0.406991	-0.983786	-2.647449
H	-1.094828	-0.288159	-3.302582
H	-0.417345	-1.787148	-4.007425
C	-3.893524	-0.567945	0.205011
H	-4.224412	-4.541957	-1.760882
H	-4.449823	-3.952243	-0.101489
H	-2.814164	-4.268316	-0.718437
H	-5.220228	-1.054295	-2.344673
H	-5.830457	-2.045818	-0.997897
H	-5.578585	-2.774011	-2.604913
C	-4.317181	-0.852970	1.522103
C	-5.151891	0.075745	2.167354
C	-5.532116	1.266731	1.573666
C	-5.069638	1.561837	0.295855
C	-4.263979	0.665237	-0.406190
C	-3.839561	1.064830	-1.814156
H	-6.170659	1.971506	2.108671
H	-5.350550	2.505672	-0.175768
C	-3.983544	-2.076028	2.379231
H	-5.483538	-0.147707	3.184605
C	-2.673730	-2.811400	2.107928
C	-5.185326	-3.029109	2.452382
H	-3.873047	-1.655798	3.392871
H	-5.449521	-3.425948	1.462761
H	-6.074925	-2.514147	2.842228
H	-4.963015	-3.880033	3.113591
H	-2.694828	-3.389767	1.179284
H	-2.479660	-3.508692	2.936912
H	-1.836376	-2.103583	2.043864
C	-2.821363	2.205884	-1.790510
C	-5.030373	1.486932	-2.688273
H	-3.355972	0.198089	-2.278992
H	-5.417331	2.467483	-2.373389
H	-5.868090	0.777530	-2.648335
H	-4.710349	1.587452	-3.736247
H	-3.287093	3.123000	-1.400858
H	-2.461576	2.420823	-2.809597
H	-1.956811	1.966945	-1.156893
C	2.432223	-1.916528	-1.386659
C	1.320480	-2.754274	0.566587
C	1.943868	-3.986453	0.396749
C	3.064427	-3.146860	-1.560699
C	2.825624	-4.189080	-0.666807
H	3.318568	-5.153374	-0.799800
H	1.738470	-4.797375	1.098346
H	2.611410	-1.107127	-2.097237
H	3.747595	-3.290513	-2.400605
H	0.614926	-2.613138	1.388168

3b ν_{min} = 10 cm^{-1} *E* = −2720.5003255

C	1.757056	0.975458	0.313153
C	0.949983	-0.151469	0.192845
C	1.559199	-1.493261	0.415469
P	-0.857957	0.044500	0.411861
N	1.385804	2.328374	0.311879
C	2.475802	3.131139	0.663120
C	3.537273	2.333977	0.841251
N	3.129152	1.022653	0.615631
C	0.285378	3.027887	-0.290029
C	-0.723283	3.537537	0.543925
C	-1.640850	4.434643	-0.012419
C	-1.539507	4.825931	-1.343057
C	0.397519	3.397266	-1.645817
C	-0.537903	4.301345	-2.155638
C	1.489782	2.811725	-2.525742
H	-0.475224	4.611912	-3.199343
H	-2.254309	5.539884	-1.756433
C	-0.784541	3.182211	2.018290
H	-2.435191	4.844391	0.614070
H	2.371206	4.209463	0.714734
H	4.561425	2.553790	1.124568
C	4.107683	-0.019369	0.684335
C	4.402995	-0.625630	1.913806
C	5.396771	-1.617376	1.914776
C	6.099298	-1.947855	0.766098
C	5.813241	-1.301438	-0.435118
C	4.806685	-0.340892	-0.501873
C	4.442386	0.316164	-1.826876
H	6.366245	-1.572029	-1.335220
H	6.872052	-2.717412	0.801830
C	3.810980	-0.270341	3.278801
H	5.622542	-2.129536	2.853406
C	-0.207807	4.314613	2.875454
H	0.840421	4.524416	2.617672
H	-0.247778	4.045925	3.941656
H	-0.784961	5.242000	2.735345
H	-0.157074	2.291243	2.164958
C	-2.204386	2.822086	2.450399
H	-2.870734	3.699179	2.443861
H	-2.200096	2.419116	3.474984
H	-2.625849	2.059479	1.781923
H	2.346713	2.573254	-1.874842
C	1.990471	3.780300	-3.596737
C	1.003443	1.501040	-3.155483
H	0.648887	0.803117	-2.383822
H	1.813884	1.015567	-3.722750
H	0.169466	1.697276	-3.847696
H	2.290835	4.744539	-3.161893
H	1.223149	3.972106	-4.361666
H	2.860099	3.349352	-4.113956
C	4.674835	-0.599449	-3.031481
C	5.194248	1.636857	-2.037288
H	3.364334	0.544009	-1.787085
H	5.748098	-0.735513	-3.232942
H	4.231389	-0.151004	-3.932459
H	4.229102	-1.594838	-2.887815
H	6.280741	1.458943	-2.048459
H	4.972407	2.367552	-1.249072
H	4.911660	2.086103	-3.001785
C	2.493537	0.507167	3.360324
C	4.877420	0.469092	4.103224
H	3.633287	-1.245684	3.763168
H	5.101794	1.443842	3.642486
H	5.816532	-0.097471	4.167517
H	4.512590	0.654378	5.124243
H	2.620463	1.566828	3.094468
H	2.140305	0.473612	4.401801
H	1.706525	0.087084	2.724153
C	-1.752495	-1.267944	-0.287436
N	-3.112237	-1.338851	0.012865
C	-1.423479	-2.431385	-1.252113
C	-0.727680	-3.650329	-0.598157
C	-0.507377	-1.963257	-2.408938
C	-2.836112	-2.817880	-1.760315

C	-3.807664	-2.493033	-0.626987
C	-5.199244	-2.140736	-1.147268
C	-3.980839	-3.692271	0.316741
H	-2.920615	-3.869193	-2.059604
H	-3.087821	-2.205661	-2.640674
H	0.304853	-3.371715	-0.385358
C	-0.703836	-4.864285	-1.542447
H	-1.167809	-3.908332	0.373560
H	0.226473	-1.252096	-2.005487
H	-1.116460	-1.399670	-3.135231
C	0.243344	-3.118303	-3.101747
C	-3.859691	-0.213932	0.502294
H	-4.461113	-4.514216	-0.234159
H	-4.628869	-3.429382	1.161268
H	-3.030749	-4.063164	0.712983
H	-5.167373	-1.348485	-1.899185
H	-5.863823	-1.824334	-0.329729
H	-5.633593	-3.035134	-1.616219
C	-4.273273	-0.109576	1.849246
C	-5.088648	0.977441	2.209602
C	-5.456586	1.959344	1.306729
C	-5.000191	1.876125	-0.004375
C	-4.215600	0.804161	-0.429934
C	-3.798934	0.787597	-1.896442
H	-6.079559	2.796807	1.624920
H	-5.270288	2.654466	-0.720869
C	-3.928921	-1.026649	3.027268
H	-5.411217	1.058013	3.250805
C	-2.666859	-1.879834	2.940012
C	-5.149520	-1.852034	3.459227
H	-3.741159	-0.316663	3.850380
H	-5.462515	-2.554737	2.674990
H	-6.011122	-1.206613	3.681076
H	-4.915926	-2.434939	4.362654
H	-2.774279	-2.727158	2.256140
H	-2.440722	-2.280663	3.939681
H	-1.811609	-1.280053	2.602615
C	-2.764360	1.872923	-2.196466
C	-4.992475	0.979239	-2.846117
H	-3.332923	-0.183001	-2.106827
C	2.381922	-2.107363	-0.536636
C	1.306330	-2.201335	1.605185
C	1.877810	-3.447921	1.841213
C	2.964709	-3.353078	-0.304770
C	2.715480	-4.030666	0.887235
H	3.166362	-5.007129	1.071102
H	1.665029	-3.971971	2.774950
H	2.556651	-1.589716	-1.481476
H	3.613243	-3.796442	-1.063839
H	0.633399	-1.765365	2.346055
H	-4.690602	0.754483	-3.880116
H	-5.337012	2.023932	-2.831021
H	-5.854636	0.348568	-2.591018
H	-2.416353	1.792886	-3.238733
H	-1.895186	1.801984	-1.529844
H	-3.212288	2.869213	-2.069110
C	-0.505008	-4.447965	-3.014124
H	-1.620329	-5.467227	-1.439338
H	0.125554	-5.516618	-1.227917
H	-1.475666	-4.349375	-3.525307
H	0.045026	-5.230284	-3.557577
H	1.225790	-3.251953	-2.622853
H	0.443556	-2.855129	-4.151010

4a ν_{min} = 17 cm^{-1} *E* = −2603.7376240

C	1.701494	0.839366	0.290381
C	0.914179	-0.178826	-0.356758
C	1.627463	-1.354679	-0.932326
P	-0.802450	-0.048534	-0.011711
N	1.351815	2.147272	0.479596
C	2.286803	2.772506	1.286996
C	3.243931	1.862526	1.572742
N	2.894231	0.682223	0.949896
C	0.321789	2.954595	-0.137572
C	-0.613765	3.567034	0.711483
C	-1.446575	4.543962	0.154703
C	-1.374434	4.861405	-1.194480
C	0.408451	3.238649	-1.516409
C	-0.475454	4.193570	-2.024122
C	1.443497	2.569054	-2.408769
H	-0.445476	4.441824	-3.084572
H	-2.032166	5.625106	-1.611828
C	-0.771889	3.195996	2.177849
H	-2.173297	5.050963	0.792665
H	2.189827	3.821459	1.546644
H	4.149388	1.939537	2.164698
C	3.686879	-0.515437	1.105501
C	3.470529	-1.344848	2.217614
C	4.253288	-2.504930	2.299862
C	5.206753	-2.809131	1.339018
C	5.433247	-1.935990	0.277646
C	4.691962	-0.761191	0.144994
C	5.012952	0.243739	-0.953805
H	6.203884	-2.172760	-0.455501
H	5.793323	-3.724875	1.424329
C	2.552880	-1.060869	3.405639
H	4.105376	-3.176533	3.148329
C	-0.489362	4.386594	3.099311
H	0.515546	4.803980	2.940153
H	-0.569804	4.082030	4.152391
H	-1.214601	5.196463	2.930340
H	-0.049060	2.401463	2.411882
C	-2.166821	2.616854	2.436636
H	-2.952567	3.363191	2.239290
H	-2.260342	2.303589	3.487170
H	-2.357017	1.740852	1.800099
H	1.462043	1.507683	-2.120606
C	2.847733	3.149418	-2.186894
C	1.084470	2.618065	-3.893145
H	0.061516	2.260448	-4.077359
H	1.777272	1.982448	-4.463347
H	1.172850	3.636810	-4.298913
H	3.193329	3.040012	-1.148464
H	2.861777	4.220757	-2.437317
H	3.575180	2.640234	-2.837724
C	5.708769	-0.375895	-2.166378
C	5.900411	1.380276	-0.421001
H	4.060338	0.684891	-1.290195
H	6.752313	-0.634106	-1.932034
H	5.735815	0.351617	-2.990247
H	5.203370	-1.283066	-2.517446
H	6.844722	0.970555	-0.032766
H	5.425140	1.956886	0.381095
H	6.142380	2.080343	-1.234049
C	1.192190	-0.416320	3.137448
C	3.319321	-0.273905	4.478565
H	2.343280	-2.054301	3.832191
H	3.543687	0.745960	4.132042
H	4.268218	-0.764259	4.734987
H	2.714795	-0.188550	5.393036
H	1.272460	0.659370	2.918875
H	0.569082	-0.510240	4.038620
H	0.658297	-0.900465	2.309554
C	-1.749857	-1.190211	-1.004170
N	-2.981149	-1.509205	-0.564462
C	-1.546660	-1.762877	-2.416003
C	-0.668395	-3.035646	-2.461724
C	-0.976116	-0.690462	-3.350539
C	-3.007236	-2.086866	-2.815274

C	-3.742766	-2.407615	-1.515101
C	-5.232326	-2.090561	-1.567394
C	-3.596188	-3.882240	-1.126364
H	-3.064676	-2.911657	-3.537752
H	-3.466917	-1.201888	-3.282151
H	0.373640	-2.792575	-2.688246
H	-1.033315	-3.702000	-3.255997
H	-0.673959	-3.592442	-1.516704
H	0.040524	-0.402376	-3.053269
H	-1.607791	0.211238	-3.345568
H	-0.935784	-1.083712	-4.377824
C	-3.632242	-0.854391	0.551686
H	-4.158020	-4.489381	-1.849211
H	-4.020058	-4.065911	-0.132470
H	-2.557078	-4.225158	-1.137222
H	-5.435439	-1.080259	-1.931284
H	-5.703208	-2.217663	-0.581969
H	-5.705232	-2.797293	-2.262894
C	-3.593404	-1.366901	1.866683
C	-4.304477	-0.662254	2.852360
C	-5.028125	0.484646	2.572499
C	-5.029327	0.986915	1.277150
C	-4.327324	0.349503	0.254253
C	-4.325579	1.026446	-1.113120
H	-5.576136	0.996552	3.364535
H	-5.574907	1.905583	1.055864
C	-2.891425	-2.618744	2.403861
H	-4.279727	-1.043346	3.875818
C	-1.661089	-3.143055	1.669616
C	-3.909534	-3.734375	2.683259
H	-2.521622	-2.297859	3.391304
H	-4.389496	-4.092993	1.763004
H	-4.707848	-3.384383	3.351615
H	-3.413597	-4.590886	3.162030
H	-1.894926	-3.576587	0.691721
H	-1.193567	-3.931067	2.277812
H	-0.922628	-2.344510	1.523643
C	-3.444748	2.279271	-1.104286
C	-5.738195	1.412323	-1.576335
H	-3.897307	0.333087	-1.848443
H	-6.108253	2.285466	-1.019847
H	-6.467667	0.602704	-1.439543
H	-5.722616	1.692136	-2.639534
H	-3.816875	3.006127	-0.365627
H	-3.461959	2.765671	-2.091671
H	-2.399271	2.052772	-0.853305
C	2.310088	-1.268691	-2.154086
C	1.635143	-2.581371	-0.250347
C	2.303504	-3.685603	-0.769893
C	2.946895	-2.386247	-2.695075
C	2.951917	-3.595577	-2.002783
H	3.458565	-4.465697	-2.422262
H	2.312809	-4.623906	-0.213141
H	2.322707	-0.324866	-2.704646
H	3.440971	-2.310058	-3.664951
H	1.122922	-2.658228	0.710986

4b ν_{min} = 17 cm^{-1} *E* = −2720.3315519

C	1.769657	1.036613	0.035590
C	0.944465	-0.135867	-0.092906
C	1.613929	-1.467144	-0.118116
P	-0.763559	0.170502	0.162566
N	1.456308	2.308618	-0.354776
C	2.434331	3.188512	0.078192
C	3.381505	2.459025	0.707463
N	2.982840	1.138707	0.667540
C	0.425673	2.804623	-1.240277
C	-0.463420	3.760851	-0.724516
C	-1.286915	4.431763	-1.635219
C	-1.251273	4.129925	-2.989621
C	0.473032	2.458620	-2.606538
C	-0.400377	3.132836	-3.462928
C	1.462740	1.433192	-3.139425
H	-0.399258	2.894239	-4.526172
H	-1.901465	4.662577	-3.685015
C	-0.582536	4.066170	0.760511
H	-1.977937	5.193110	-1.267842
H	2.368622	4.249254	-0.140131
H	4.310805	2.751965	1.183836
C	3.760283	0.096424	1.294755
C	3.581012	-0.168758	2.661333
C	4.347656	-1.208078	3.206932
C	5.248588	-1.928907	2.436401
C	5.437036	-1.606359	1.094257
C	4.710816	-0.576110	0.496253
C	4.987697	-0.157689	-0.941975
H	6.165449	-2.164509	0.506585
H	5.823153	-2.739775	2.886202
C	2.715889	0.624633	3.639017
H	4.228311	-1.445872	4.266222
C	-0.259171	5.531159	1.069171
H	0.744817	5.815085	0.721484
H	-0.309887	5.713192	2.151975
H	-0.980526	6.205617	0.584268
H	0.137605	3.435349	1.301068
C	-1.976332	3.687283	1.272414
H	-2.758843	4.287585	0.781711
H	-2.047095	3.866857	2.355597
H	-2.191674	2.625178	1.086622
H	1.498630	0.618118	-2.402389
C	2.874891	2.023200	-3.261234
C	1.030310	0.808359	-4.465327
H	-0.004159	0.437846	-4.420653
H	1.688145	-0.038956	-4.708659
H	1.105676	1.524330	-5.297183
H	3.260468	2.391485	-2.298793
H	2.878851	2.863248	-3.971841
H	3.575046	1.259674	-3.633927
C	5.610282	-1.265827	-1.792128
C	5.916706	1.065945	-0.992632
H	4.024246	0.125924	-1.397523
H	6.660527	-1.434823	-1.511085
H	5.605522	-0.968260	-2.850546
H	5.073283	-2.216522	-1.693864
H	6.875424	0.831063	-0.506839
H	5.492722	1.947948	-0.498506
H	6.123054	1.338195	-2.038115
C	1.358894	1.142054	3.157441
C	3.538538	1.765710	4.254725
H	2.502506	-0.085787	4.453069
H	3.772671	2.531786	3.500468
H	4.485174	1.397749	4.673185
H	2.970175	2.254174	5.059303
H	1.452991	2.025196	2.507353
H	0.766814	1.453628	4.030075
H	0.785520	0.375085	2.620524
C	-1.767896	-1.263958	-0.217196
N	-2.989551	-1.287620	0.348620
C	-1.650068	-2.404869	-1.245303
C	-0.792791	-3.618075	-0.809948
C	-1.046271	-1.870363	-2.572776
C	-3.139794	-2.813482	-1.401142

C	-3.828671	-2.465441	-0.083196
C	-5.304562	-2.124036	-0.255604
C	-3.741622	-3.608674	0.932926
H	-3.271535	-3.874978	-1.639116
H	-3.598731	-2.232934	-2.216468
H	0.253048	-3.313033	-0.827992
C	-0.961767	-4.797647	-1.783293
H	-0.998033	-3.925565	0.223582
H	-0.264488	-1.131692	-2.338709
H	-1.832251	-1.322130	-3.118217
C	-0.442390	-2.980382	-3.452641
C	-3.572216	-0.179877	1.077776
H	-4.357165	-4.444024	0.572196
H	-4.139717	-3.292774	1.904309
H	-2.721941	-3.979568	1.073802
H	-5.476474	-1.381768	-1.038686
H	-5.747088	-1.764164	0.684288
H	-5.830446	-3.043290	-0.547706
C	-3.500068	-0.066301	2.483075
C	-4.163930	1.022161	3.073772
C	-4.873171	1.953388	2.334391
C	-4.904104	1.839441	0.949950
C	-4.248721	0.796468	0.296093
C	-4.271861	0.809552	-1.229696
H	-5.385082	2.777411	2.832997
H	-5.436363	2.587049	0.359750
C	-2.796598	-0.970491	3.502230
H	-4.112448	1.128207	4.159764
C	-1.619504	-1.824737	3.041602
C	-3.817456	-1.795784	4.299801
H	-2.367232	-0.252538	4.220145
H	-4.337756	-2.527866	3.668142
H	-4.583367	-1.152489	4.753554
H	-3.312167	-2.346138	5.106344
H	-1.913413	-2.641991	2.374776
H	-1.140932	-2.273839	3.924128
H	-0.869299	-1.214498	2.523567
C	-3.356833	1.906430	-1.781677
C	-5.686579	1.014431	-1.792285
H	-3.886350	-0.150757	-1.597046
C	2.213803	-1.956268	-1.285642
C	1.638344	-2.270175	1.033868
C	2.241942	-3.523232	1.018606
C	2.789947	-3.227991	-1.310545
C	2.810085	-4.012940	-0.159752
H	3.266604	-5.003442	-0.179155
H	2.263539	-4.124908	1.928474
H	2.198815	-1.353203	-2.197066
H	3.222000	-3.607389	-2.238401
H	1.187373	-1.894596	1.954498
H	-5.699359	0.792985	-2.869213
H	-6.006152	2.060116	-1.676218
H	-6.438395	0.384838	-1.297834
H	-3.392770	1.915970	-2.881834
H	-2.310240	1.771583	-1.475906
H	-3.686086	2.895191	-1.426385
C	-1.131441	-4.327265	-3.240602
H	-1.806691	-5.438837	-1.488310
H	-0.062836	-5.427037	-1.699008
H	-2.198118	-4.230193	-3.497916
H	-0.722498	-5.078547	-3.930250
H	0.625289	-3.098260	-3.208931
H	-0.488673	-2.679548	-4.509026

5a ν_{min} = 16 cm^{-1} *E* = −2603.4441379

C	1.736398	0.752621	0.480025
C	0.845181	-0.015666	-0.424143
C	1.499033	-0.963564	-1.366837
P	-0.795229	0.302866	-0.162519
N	1.663537	2.067086	0.790165
C	2.641233	2.373091	1.705028
C	3.318255	1.220643	1.970914
N	2.755879	0.233428	1.202825
C	0.771140	3.070751	0.239973
C	-0.322269	3.483867	1.020605
C	-1.139211	4.482509	0.482529
C	-0.879032	5.024206	-0.772654
C	1.093306	3.626302	-1.010245
C	0.224798	4.601543	-1.509112
C	2.381237	3.291477	-1.742903
H	0.432828	5.061706	-2.475831
H	-1.530869	5.801496	-1.173896
C	-0.584437	2.944013	2.416826
H	-1.991239	4.844644	1.060466
H	2.768785	3.383737	2.081604
H	4.132297	1.015900	2.658346
C	3.166220	-1.159248	1.211428
C	2.476335	-2.070563	2.029358
C	2.817328	-3.422459	1.871168
C	3.815224	-3.826878	0.995005
C	4.541279	-2.880342	0.275341
C	4.240845	-1.521011	0.369675
C	5.105672	-0.487887	-0.340184
H	5.354893	-3.207613	-0.371531
H	4.057317	-4.886116	0.898078
C	1.567822	-1.752729	3.219365
H	2.304211	-4.165679	2.485968
C	-0.170804	3.981401	3.469109
H	0.879224	4.287218	3.357706
H	-0.305603	3.576810	4.481971
H	-0.788188	4.887586	3.381134
H	0.039354	2.049606	2.556549
C	-2.035643	2.508435	2.624641
H	-2.730923	3.358816	2.553022
H	-2.154963	2.074484	3.628163
H	-2.339297	1.749741	1.889705
H	2.750388	2.322588	-1.369335
C	3.451095	4.341007	-1.407833
C	2.195736	3.170007	-3.256628
H	1.373327	2.487437	-3.517477
H	3.119232	2.795310	-3.720954
H	1.981775	4.145332	-3.715662
H	3.636045	4.401004	-0.325671
H	3.131155	5.336423	-1.748776
H	4.399886	4.097068	-1.906103
C	5.791660	-1.024551	-1.598464
C	6.195332	0.034531	0.611858
H	4.458449	0.357996	-0.632973
H	6.617959	-1.701112	-1.336514
H	6.230187	-0.192228	-2.165958
H	5.099730	-1.567822	-2.251973
H	6.845478	-0.795696	0.923424
H	5.793977	0.496862	1.521259
H	6.819457	0.780728	0.100670
C	0.654440	-0.527160	3.188057
C	2.449163	-1.696426	4.478941
H	0.912488	-2.632930	3.318876
H	3.140225	-0.840607	4.427369
H	3.051049	-2.607464	4.591569
H	1.828538	-1.576602	5.377983
H	1.219338	0.413352	3.265961
H	-0.008345	-0.561721	4.064911
H	0.007728	-0.487958	2.300942
C	-1.801312	-0.768752	-1.333595
N	-2.889229	-1.256346	-0.817008
C	-1.817984	-0.838515	-2.867510
C	-0.789721	-1.732496	-3.575507
C	-1.628802	0.607638	-3.363583
C	-3.259100	-1.354202	-3.128833

C	-3.706799	-2.056410	-1.843960
C	-5.199885	-1.996118	-1.571666
C	-3.244703	-3.514057	-1.813936
H	-3.291138	-2.042871	-3.982375
H	-3.932252	-0.514855	-3.357928
H	0.223445	-1.329258	-3.508579
H	-1.066489	-1.758343	-4.638553
H	-0.784236	-2.762781	-3.203402
H	-0.602561	0.955193	-3.169451
H	-2.334288	1.307604	-2.890897
H	-1.799360	0.633039	-4.449502
C	-3.441860	-0.885343	0.492172
H	-3.786533	-4.059294	-2.598252
H	-3.475017	-3.989131	-0.854452
H	-2.169921	-3.614094	-2.008353
H	-5.600630	-0.979619	-1.625678
H	-5.445919	-2.428754	-0.591753
H	-5.703005	-2.596578	-2.341903
C	-3.216622	-1.615307	1.678834
C	-3.847521	-1.138953	2.840388
C	-4.670917	-0.025811	2.841556
C	-4.868866	0.675212	1.658458
C	-4.259818	0.278126	0.468548
C	-4.487679	1.160651	-0.757233
H	-5.153329	0.300230	3.763642
H	-5.503000	1.562588	1.659062
C	-2.430140	-2.907677	1.897426
H	-3.685349	-1.685865	3.771587
C	-1.204728	-3.142870	1.023733
C	-3.381065	-4.113439	1.937624
H	-2.036488	-2.803379	2.921057
H	-3.931439	-4.242474	0.996243
H	-4.129555	-3.992540	2.732068
H	-2.820276	-5.037494	2.136295
H	-1.445649	-3.323853	-0.030137
H	-0.662203	-4.028957	1.386112
H	-0.513562	-2.289148	1.089265
C	-3.667083	2.454341	-0.672454
C	-5.968187	1.514344	-0.958807
H	-4.154405	0.618825	-1.651084
H	-6.304466	2.253035	-0.217993
H	-6.628374	0.640380	-0.875904
H	-6.112740	1.966297	-1.949971
H	-3.962933	3.039353	0.211635
H	-3.844011	3.075732	-1.563228
H	-2.584833	2.274465	-0.593845
C	2.282232	-0.442222	-2.408359
C	1.339518	-2.349664	-1.252598
C	1.946528	-3.201199	-2.173829
C	2.847897	-1.296975	-3.351726
C	2.685011	-2.678857	-3.234702
H	3.141824	-3.346602	-3.966385
H	1.835543	-4.280997	-2.063506
H	2.408876	0.636210	-2.510275
H	3.426370	-0.881423	-4.178185
H	0.774777	-2.765284	-0.420190

5b ν_{min} = 12 cm^{-1} *E* = −2720.0399323

C	1.839507	0.974966	0.163892
C	0.895887	−0.112894	−0.189260
C	1.486368	−1.413100	−0.611351
P	−0.724912	0.327687	0.007502
N	1.755178	2.281746	−0.177434
C	2.804339	2.967335	0.385079
C	3.539501	2.066497	1.094941
N	2.939349	0.843075	0.941657
C	0.788623	2.938991	−1.038695
C	−0.197512	3.736780	−0.432924
C	−1.059877	4.431713	−1.285692
C	−0.952277	4.314493	−2.667752
C	0.951340	2.823345	−2.430073
C	0.038420	3.515051	−3.231951
C	2.122477	2.088674	−3.058853
H	0.126901	3.456804	−4.317549
H	−1.636523	4.865321	−3.314636
C	−0.316373	3.901541	1.073016
H	−1.828095	5.077668	−0.856720
H	2.933599	4.033784	0.225341
H	4.421915	2.195782	1.712953
C	3.406560	−0.382655	1.563349
C	2.846338	−0.787462	2.786826
C	3.241875	−2.047815	3.259964
C	4.171132	−2.824405	2.581393
C	4.770342	−2.344255	1.418902
C	4.408566	−1.108144	0.882545
C	5.137582	−0.551349	−0.333079
H	5.531647	−2.944330	0.921002
H	4.459113	−3.798671	2.978715
C	2.027498	0.059944	3.762920
H	2.828320	−2.403601	4.206468
C	0.156796	5.296558	1.500192
H	1.186140	5.501851	1.173340
H	0.117196	5.397464	2.593801
H	−0.488034	6.074690	1.065715
H	0.338970	3.158446	1.549085
C	−1.734582	3.626833	1.576817
H	−2.453889	4.364206	1.188345
H	−1.762362	3.690320	2.674514
H	−2.077835	2.624149	1.285218
H	2.547166	1.405875	−2.305698
C	3.224162	3.091490	−3.430189
C	1.715018	1.250189	−4.272445
H	0.874541	0.577172	−4.043456
H	2.564976	0.641580	−4.613935
H	1.417357	1.884398	−5.119164
H	3.562171	3.663910	−2.554374
H	2.854765	3.808946	−4.177407
H	4.092591	2.570094	−3.856874
C	5.701680	−1.634933	−1.254786
C	6.299692	0.353052	0.111566
H	4.420933	0.056607	−0.913529
H	6.585028	−2.111625	−0.805765
H	6.030552	−1.183601	−2.201008
H	4.966719	−2.417266	−1.476182
H	7.019695	−0.230626	0.703183
H	5.979397	1.202217	0.726652
H	6.826670	0.752261	−0.766312
C	1.056341	1.116489	3.235444
C	3.005555	0.717246	4.751435
H	1.425728	−0.665841	4.332899
H	3.648136	1.444570	4.231045
H	3.657677	−0.025626	5.228760
H	2.455798	1.253210	5.537789
H	1.580628	1.982659	2.805894
H	0.464891	1.501305	4.078827
H	0.344342	0.722569	2.497551
C	−1.829096	−1.125348	−0.468661
N	−2.892054	−1.238215	0.270855
C	−1.932381	−1.951711	−1.753232
C	−0.972621	−3.156621	−1.899456
C	−1.654642	−0.987904	−2.950887
C	−3.410694	−2.428220	−1.676905

C	-3.809000	-2.373767	-0.200372
C	-5.279721	-2.081734	0.048656
C	-3.427606	-3.659832	0.534698
H	-3.540153	-3.443912	-2.067550
H	-4.059025	-1.765148	-2.268549
H	0.038189	-2.767221	-2.026230
C	-1.329835	-3.993790	-3.140251
H	-0.956535	-3.783726	-0.999671
H	-0.873951	-0.261629	-2.667630
H	-2.565229	-0.400451	-3.151112
C	-1.196902	-1.730857	-4.217580
C	-3.338441	-0.259467	1.265857
H	-4.063750	-4.469785	0.153642
H	-3.606188	-3.571901	1.611647
H	-2.381657	-3.942636	0.369963
H	-5.636674	-1.198155	-0.487851
H	-5.488937	-1.962074	1.120977
H	-5.853408	-2.946337	-0.312218
C	-3.047838	-0.351056	2.642864
C	-3.599315	0.644096	3.467899
C	-4.404411	1.658538	2.978506
C	-4.658581	1.730057	1.613685
C	-4.129396	0.793019	0.727421
C	-4.401154	1.004701	-0.761649
H	-4.825759	2.401018	3.657096
H	-5.273006	2.542281	1.223534
C	-2.249828	-1.408551	3.407475
H	-3.386701	0.598034	4.538108
C	-1.109793	-2.106170	2.674065
C	-3.192623	-2.391644	4.117708
H	-1.762353	-0.832170	4.210070
H	-3.814567	-2.958836	3.412883
H	-3.875807	-1.859360	4.792816
H	-2.614472	-3.109365	4.716322
H	-1.443206	-2.772907	1.870046
H	-0.543421	-2.722189	3.388248
H	-0.410648	-1.367337	2.255374
C	-3.523883	2.124883	-1.335388
C	-5.875469	1.323516	-1.048305
H	-4.149598	0.084776	-1.304821
C	2.113262	-1.497259	-1.863779
C	1.406102	-2.552635	0.198130
C	1.936235	-3.762656	-0.245236
C	2.602465	-2.720477	-2.318600
C	2.518179	-3.855164	-1.509239
H	2.915096	-4.808275	-1.861559
H	1.887067	-4.640837	0.400280
H	2.172691	-0.617522	-2.505364
H	3.060821	-2.784516	-3.306803
H	0.959011	-2.487039	1.189187
H	-6.069632	1.254572	-2.127671
H	-6.126045	2.349121	-0.743208
H	-6.565498	0.646197	-0.527015
H	-3.757415	2.282355	-2.398944
H	-2.446907	1.915448	-1.256427
H	-3.710391	3.070249	-0.803390
C	-1.821736	-3.120512	-4.307059
H	-2.072445	-4.767810	-2.897100
H	-0.419963	-4.535923	-3.438128
H	-2.919556	-3.024774	-4.288798
H	-1.576447	-3.594507	-5.266295
H	-0.099330	-1.836960	-4.205782
H	-1.442175	-1.124631	-5.100247

References

1. Rottschäfer, D.; Sharma, M.; Neumann, B.; Stammler, H.-G.; Andrada, D. M.; Ghadwal, R. S., A Modular Access to Divinyldiphosphenes with a Strikingly Small HOMO-LUMO Energy Gap. *Chem. Eur. J.* 2019, **25**, 8127–8134.
2. Lavallo, V.; Canac, Y.; Präsang, C.; Donnadieu, B.; Bertrand, G., Stable Cyclic (Alkyl)(Amino)Carbenes as Rigid or Flexible, Bulky, Electron-Rich Ligands for Transition-Metal Catalysts: A Quaternary Carbon Atom Makes the Difference. *Angew. Chem. Int. Ed.* 2005, **44**, 5705-5709.
3. Fulmer, G. R., Miller, A. J. M., Sherden, N. H., Gottlieb, H. E., Nudelman, A., Stoltz, B. M., Bercaw, J. E., Goldberg, K. I., NMR Chemical Shifts of Trace Impurities: Common Laboratory Solvents, Organics, and Gases in Deuterated Solvents Relevant to the Organometallic Chemist, *Organometallics* 2010, **29**, 2176–2179.
4. Dolomanov, O. V., Bourhis, L. J., Gildea, R. J., Howard, J. A. K., Puschmann, H., OLEX2: a complete structure solution, refinement and analysis program, *J. Appl. Cryst.* 2009, **42**, 339–341.
5. Sheldrick, G. M., A short history of SHELX, *Acta Cryst.* 2008, **A64**, 112–122.
6. Sheldrick, G. M., Crystal structure refinement with SHELXL, *Acta Cryst.* 2015, **C71**, 3–8.
7. Frisch, M. J., Trucks, G. W., Schlegel, H. B., Scuseria, G. E., Robb, M. A., Cheeseman, J. R., Scalmani, G., Barone, V., Mennucci, B., Petersson, G. A., Nakatsuji, H., Caricato, M., Li, X., Hratchian, H. P., Izmaylov, A. F., Bloino, J., Zheng, G., Sonnenberg, J. L., Hada, M., Ehara, M., Toyota, K., Fukuda, R., Hasegawa, J., Ishida, M., Nakajima, T., Honda, Y., Kitao, O., Nakai, H., Vreven, T., Montgomery, J. J. A., Peralta, J. E., Ogliaro, F., Bearpark, M., Heyd, J. J., Brothers, E., Kudin, K. N., Staroverov, V. N., Kobayashi, R., Normand, J., Raghavachari, K., Rendell, A., Burant, J. C., Iyengar, S. S., Tomasi, J., Cossi, M., Rega, N., Millam, J. M., Klene, M., Knox, J. E., Cross, J. B., Bakken, V., Adamo, C., Jaramillo, J., Gomperts, R., Stratmann, R. E., Yazyev, O., Austin, A. J., Cammi, R., Pomelli, C., Ochterski, J. W., Martin, R. L., Morokuma, K., Zakrzewski, V. G., Voth, G. A., Salvador, P., Dannenberg, J. J., Dapprich, S., Daniels, A. D., Farkas, O., Foresman, J. B., Ortiz, J. V., Cioslowski, J., Fox, D. J., Gaussian 16, Revision A.03. Gaussian, Inc., Wallingford CT, 2016.
8. Zhao, Y., Truhlar, D. G., The M06 suite of density functionals for main group thermochemistry, thermochemical kinetics, noncovalent interactions, excited states, and transition elements: two new functionals and systematic testing of four M06-class functionals and 12 other functionals, *Theor. Chem. Acc.* 2008, **120**, 215–241.
9. Weigend, F., Ahlrichs, R., Balanced basis sets of split valence, triple zeta valence and quadruple zeta valence quality for H to Rn: Design and assessment of accuracy, *Phys. Chem. Chem. Phys.* 2005, **7**, 3297–3305.

10. McIver, J. W., Komornic. A, Structure of transition states in organic reactions. General theory and an application to the cyclobutene-butadiene isomerization using a semiempirical molecular orbital method, *J. Am. Chem. Soc.* 1972, **94**, 2625–2633.
11. Wiberg, K. B., Application of the pople-santry-segal CNDO method to the cyclopropylcarbinyl and cyclobutyl cation and to bicyclobutane, *Tetrahedron* 1968, **24**, 1083–1096.
12. (a) Reed, A. E., Weinhold, F., Natural localized molecular orbitals, *J. Chem. Phys.* 1985, **83**, 1736–1740; (b) Reed, A. E., Weinstock, R. B., Weinhold, F., Natural population analysis, *J. Chem. Phys.* 1985, **83**, 735–746.
13. Glendening, E. D., Reed, A. E., Carpenter, J. E., Weinhold, F., NBO Version 3.1.
14. Neese, F., Software update: the ORCA program system, version 4.0, *WIREs Comput. Mol. Sci.* 2017, **8**, e1327.
15. Barone, V., Cossi, M., Quantum calculation of molecular energies and energy gradients in solution by a conductor solvent model, *J. Phys. Chem. A* 1998, 102, 1995.
